# Site-directed allostery perturbation to probe the negative regulation of hypoxia inducible factor-1α†

**DOI:** 10.1039/d4cb00066h

**Published:** 2024-05-30

**Authors:** Vencel L. Petrovicz, István Pasztuhov, Tamás A. Martinek, Zsófia Hegedüs

**Affiliations:** a University of Szeged, Department of Medical Chemistry 8 Dóm tér Szeged 6720 Hungary martinek.tamas@med.u-szeged.hu hegedus.zsofia@med.u-szeged.hu; b HUN-REN SZTE Biomimetic Systems Research Group 8 Dóm tér Szeged 6720 Hungary

## Abstract

The interaction between the intrinsically disordered transcription factor HIF-1α and the coactivator proteins p300/CBP is essential in the fast response to low oxygenation. The negative feedback regulator, CITED2, switches off the hypoxic response through a very efficient irreversible mechanism. The negative cooperativity with HIF-1α relies on the formation of a ternary intermediate that leads to allosteric structural changes in p300/CBP, in which the cooperative folding/binding of the CITED2 sequence motifs plays a key role. Understanding the contribution of a binding motif to the structural changes in relation to competition efficiency provides invaluable insights into the molecular mechanism. Our strategy is to site-directedly perturb the p300–CITED2 complex's structure without significantly affecting binding thermodynamics. In this way, the contribution of a sequence motif to the negative cooperativity with HIF-1α would mainly depend on the induced structural changes, and to a lesser extent on binding affinity. Using biophysical assays and NMR measurements, we show here that the interplay between the N-terminal tail and the rest of the binding motifs of CITED2 is crucial for the unidirectional displacement of HIF-1α. We introduce an advantageous approach for evaluating the roles of the different sequence parts with the help of motif-by-motif backbone perturbations.

## Abstract 2

The structural adaptation of p300 to the backbone-modified CITED2 sequences provides insight into the molecular mechanism that governs the allosteric regulation of HIF-1α.

## Graphical abstract

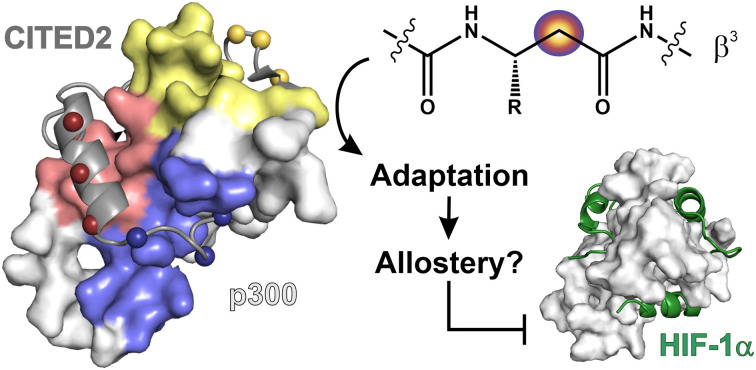

## Introduction

Intrinsically disordered proteins (IDPs) play a central role in modulating cellular processes and often regulate mechanisms that react to altered conditions.^1–4^ Gene transcriptions that switch on in response to decreased oxygenation are governed by the hypoxia-inducible factor (HIF-1),^5–7^ which is implicated in several cancer-related conditions.^8–11^ In hypoxia, the α subunit of HIF-1 (HIF-1α) translocates to the nucleus and interacts with the HIF-1β subunit, forming HIF-1.^12^ This complex recruits the multidomain hub proteins p300 and its paralog CREB binding protein (CBP), the coactivators required for the transcriptional activity of HIF-1.^13–16^ As a result, the expression of hundreds of genes associated with mechanisms adapting to oxygen-deprived conditions is upregulated.^17–19^ In a negative feedback loop, the CREB-binding protein/p300-interacting transactivator with ED-rich tail (CITED2) terminates the hypoxic response through the competition with HIF-1α for binding to p300/CBP.^20,21^ Even though CITED2 and HIF-1α have similar binding affinities to p300/CBP, the competition is so efficient that CITED2 completely displaces HIF-1α in a switch-like, irreversible mechanism, allowing the precise regulation of the hypoxic response.^22^ Several experimental^22–25^ and molecular modelling studies^26–29^ investigated the underlying structural mechanism of this competition. These works agreed on basic concepts but highlighted different key determinants or provided alternative explanations for the virtually irreversible competition.

**Fig. 1:**
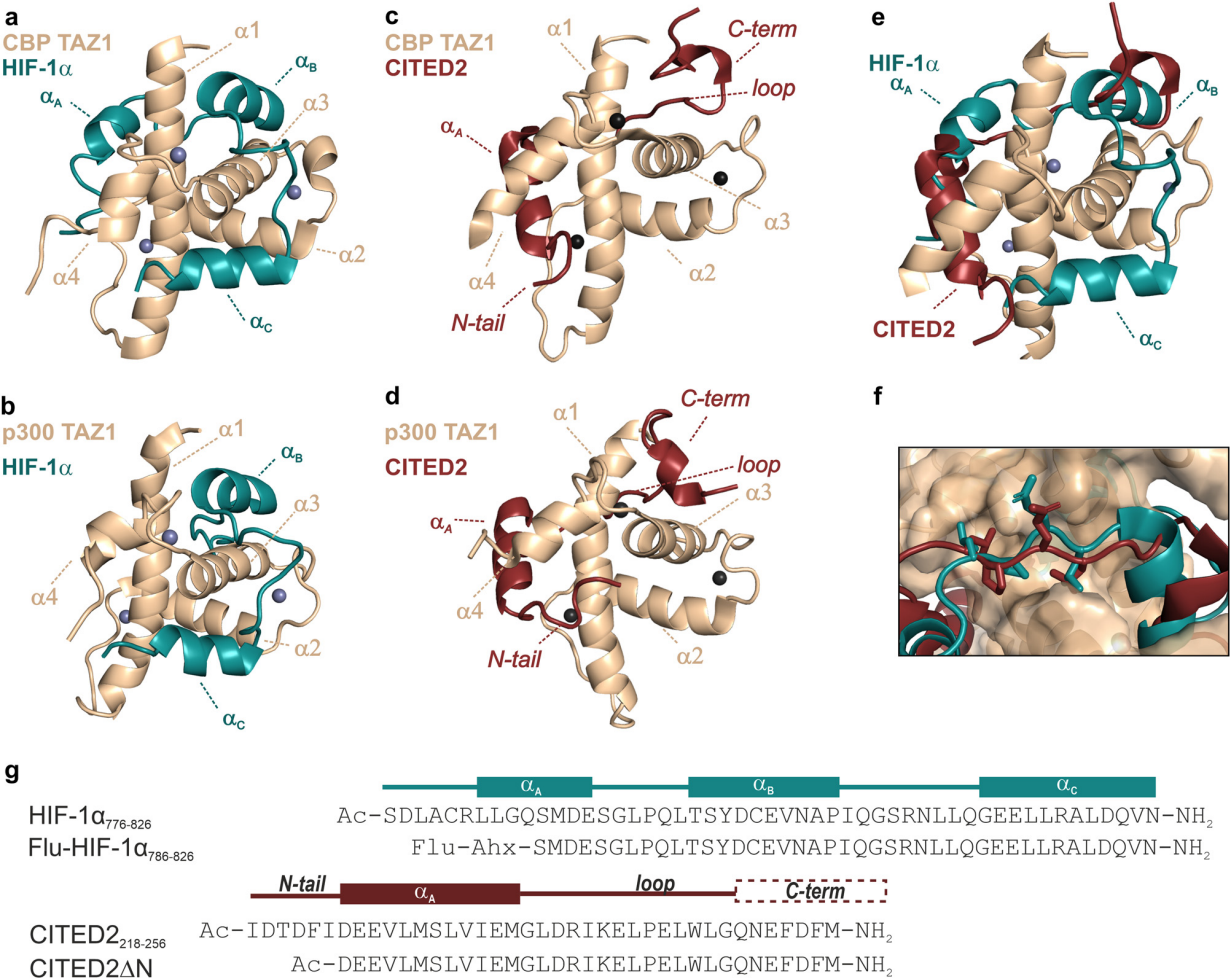
Structure of p300/CBP bound to HIF-1α and CITED2. (a) Structure of HIF-1α CTAD (blue) bound to CBP TAZ1 domain (wheat) (PDB: 1L8C)^30^ and (b) to p300 TAZ1 domain (PDB: 1L3E).^31^ The helices of the TAZ1 domain are annotated α1–α4 from N to C terminus, and the spheres represent Zn atoms. HIF-1α helices are annotated α_A_–α_C_ from N to C terminus; α_A_ was not observed for the p300-bound structure. Structures of CITED2 CTAD (red) bound to (c) CBP TAZ1 domain (wheat, PDB: 1R8U)^21^ and (d) to p300 TAZ1 domain (PDB:1P4Q).^20^ CITED2 binding motifs are annotated as N-tail, α_A_, loop, and C-term. (e) Overlayed structures of CITED2 and HIF-1α bound to TAZ1. Only one protein domain is shown for clarity. (f) Overlayed structures of p300–HIF-1α and p300–CITED2 complex showing the conserved LPE/QL motif. (g) Sequences of HIF-1α (residues 776–826 used in ITC, NMR measurements and as the competitor in FA titrations; residues 786–826 was used as the fluorescent tracer in FA titrations) and CITED2 peptides used in this study, with their binding motifs indicated above. Flu: 5,6-carboxyfluorescein, Ahx: aminohexanecarboxylic acid.

CITED2 and HIF-1α bind with their disordered C-terminal activation domain (CTAD) to the TAZ1 (or CH1) domain of p300/CBP^20,21,30,31^ and form folded structures comprising several binding motifs (Fig. 1). Both display high-affinity interactions with p300/CBP,^22,25,30^ show slow dissociation kinetics,^22,32^ and compete through a partially overlapping binding site (Fig. 1e). The competition mechanism involves the formation of a transient ternary complex in which the CITED2 α_A_ helix and the C-terminal helix/helices (α_B_ and α_C_) of HIF-1α are simultaneously bound to p300/CBP. The cooperative folding/binding of the CITED2 binding motifs drives the displacement of HIF-1α and results in an allosteric conformational change that favours the CITED2-bound state, rendering the competition virtually irreversible.^22–29^

Several factors play an important role in this complex inhibitory mechanism. These include differences in binding thermodynamics,^25^ displacement kinetics,^22,32^ and intramolecular cooperativity between binding motifs.^22,23,29^ Changes in p300/CBP dynamics^33^ and electrostatic forces^28,29^ also contribute to effective inhibition. The irreversibility of the competition depends highly on the transient ternary intermediate that results in allosteric structural changes and negative cooperativity between the two ligands.^22–24,26,29^ The binding of the CITED2 α_A_ helix alters the conformation of the α4 helix of p300/CBP and destabilises the binding of HIF-1α α_C_.^24^ The conserved LPEL motif replacing HIF-1α LPQL is a key step in the displacement process;^22,26^ mutations in this region significantly decrease competition efficiency.^23,34^

The binding of α_A_ and LPEL is not sufficient for effective inhibition, the absence of the C-terminal hydrophobic region diminishes the competition efficiency of CITED2, suggesting that the C-terminus induces unidirectionality.^23^ The removal of the C-terminus, however, also results in a significantly lower binding affinity (2 orders of magnitude in *K*_D_, Table S1, ESI†),^23,25^ which can affect the detected competition efficiency and mask the role of these residues in mediating allostery. Furthermore, our previous ITC data showed that CITED2_224–259_, comprising all three binding motifs (α_A_, loop, and C-terminus) is not an efficient competitor, suggesting that a fourth binding motif, the N-terminal tail of CITED2 (residues 218–223) plays an important role,^25^ but the exact function of the N-terminal tail and how it cooperates with the rest of the CITED2 binding motifs is currently unclear.

Because the formation of the transient ternary complex is key to the negative cooperativity between the ligands, probing the extent to which each binding motif contributes to the intermediate formation would enhance our understanding of the underlying molecular mechanism. Furthermore, identifying binding sites that can tolerate structural perturbations with conserved competition mechanisms would provide useful insights into the mimicry and/or inhibition of the system. To this end, we set out to understand the role of the N-terminal tail of CITED2 in the competition mechanism and probe its interplay with the rest of the binding motifs leading to the formation of the ternary intermediate and the negative cooperativity with HIF-1α.

Our approach is to induce site-directed structural changes in p300 by altering the bound conformation of CITED2 and linking those changes to the competition mechanism. Because the effective competition between CITED2 and HIF-1α depends both on high-affinity binding and allostery, it is important to use only modifications that minimize the effect of direct binding. In this way, the observed competition efficiency of a modified ligand is mostly dependent on the induced structural change, rather than on its binding affinity. Consequently, we avoid such modifications that substantially decrease binding affinity (*i.e.* sequence truncations) and maintain the native-like properties (intrinsic disorder, charge, side-chains, and binding thermodynamics) of CITED2 variants. The plasticity of p300/CBP allows their adaptation to various disordered ligands and participation in fuzzy interactions.^35^ We hypothesised that a similar adaptation would occur to the local conformational perturbations of the bound CITED2, resulting in close to native binding parameters, which we achieve through motif-by-motif backbone modifications. A small number of α to β^3^-amino acid replacements in CITED2 allowed us to produce native-like CITED2 variants and induce binding-site dependent structural changes in p300. Using a combination of direct binding, competition assays, and NMR measurements, here we provide further details on the underlying molecular mechanism of the p300/HIF-1α competition.

## Results

### The N-terminal residues of CITED2 drive the unidirectional replacement of HIF-1α

**Fig. 2:**
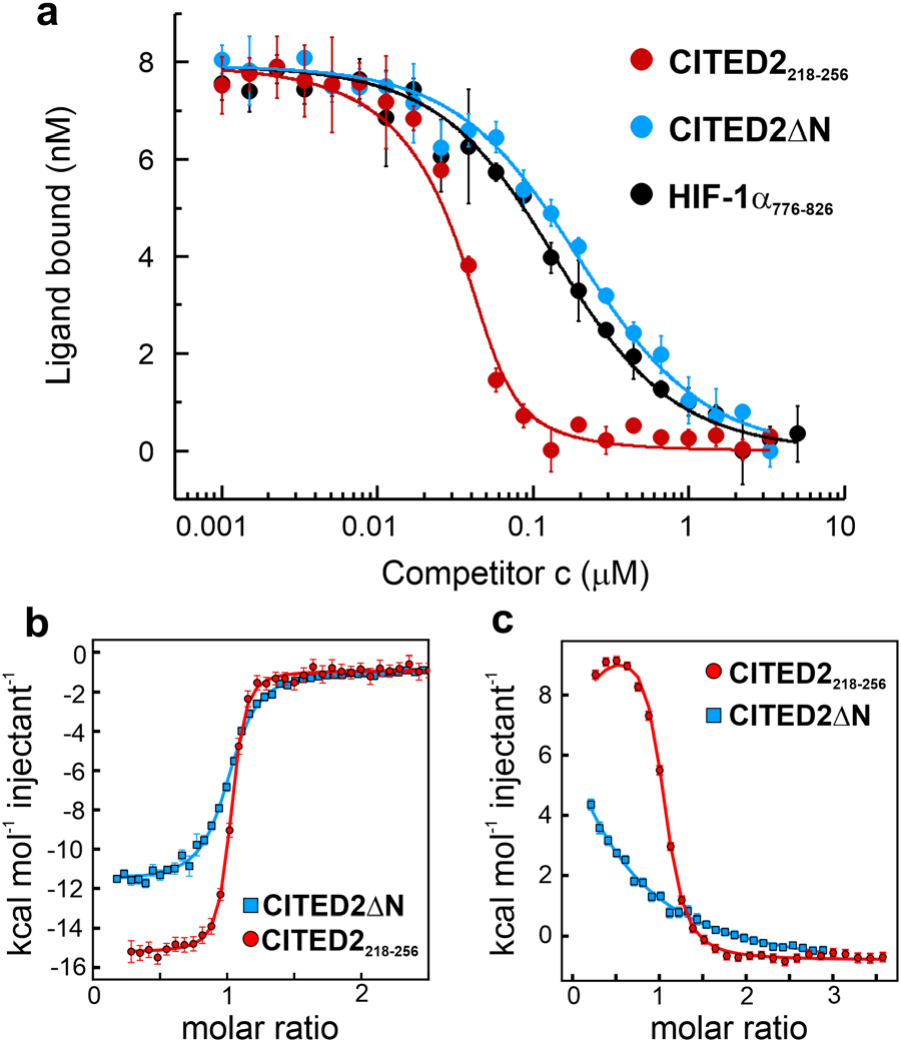
Binding and competition of different CITED2 sequences. (a) Fluorescence anisotropy (FA) competition of unlabelled CITED2_218–256_, CITED2ΔN and HIF-1α_776–826_ against fluorescein-labelled HIF-1α_786–826_ (Flu-HIF-1α) in complex with p300 using 50 nM protein and 25 nM tracer concentration, curves were fitted to a competition model using a fixed *K*_D_ value for Flu-HIF-1α (*K*_D_ = 64 nM) determined beforehand (Fig. S3, ESI†). (b) ITC thermogram for CITED2_218–256_ and CITED2ΔN binding to p300 (c) ITC thermogram for the titration of CITED2_218–256_ and CITED2ΔN to the preformed p300–HIF-1α_776–826_ complex. ITC data were fitted globally to a model including ternary intermediate formation (Fig. S20, ESI†) using constrained *K*_D_ and Δ*H* for HIF-1α (determined beforehand Fig. S2, ESI†). Fitted parameters are listed in Table 1.

The structure of the p300 and CBP TAZ1 domains is similar, but their reported complexes with CITED2 have differences in the more flexible N- and C-terminal regions (Fig. 1c–d). Considering both structures, we synthesised the shortest possible CITED2 sequence (CITED2_218–256_, Fig. 1g) comprising residues reported to be in contact with p300/CBP.^20,21^ CITED2_218–256_ therefore contains the following binding motifs: an extended N-terminal region (N-tail, residues 218–223), an α-helix (α_A_, residues 224–235) that is connected through a loop (residues 236–253) to an aromatic/hydrophobic amino acid rich C-terminus (residues 253–256). To probe the role of the N-terminal tail we compared the competition efficiency CITED2_218–256_ to a shorter sequence lacking the N-terminus (CITED2_224–256_, which we term CITED2ΔN Fig. 1g). Fluorescence anisotropy (FA) measurements indicated that CITED2_218–256_ is more potent than CITED2ΔN in competing for binding to p300_330–420_ (referred to as p300) with fluorescently labelled HIF-1α_786–826_ (Flu-HIF-1α, Fig. 2a). The fitted apparent competition *K*_D_ value of CITED2ΔN is similar to the *K*_D,app_ of HIF-1α displacing itself (86 ± 5 and 57 ± 4 nM, respectively), indicating the absence of negative cooperativity. CITED2_218–256_, however, displays an inhibition with significantly higher efficacy (*K*_D,app_ = 2.0 ± 0.9 nM). This is in line with previous observations, but the *K*_D,app_ deviates from reported values^22^ (Table S1, ESI†), which may be due to different experimental setups or protein/competitor sequences used.

To characterise the negative cooperativity between the ligands and the stability of the ternary intermediate, the following ITC titrations were performed: direct titration of HIF-1α and CITED2 variants to p300 and competition titration of CITED2 variants to a preformed p300–HIF-1α complex. The data were then globally fitted to a model that takes into account the formation of a ternary intermediate (Fig. S20, ESI†).^36–38^ From this model we extracted the direct binding affinities *K*_D_ and Δ*H* for CITED2 variants and the parameters Δ*g* and Δ*h*, which represent the additional Gibbs energy and enthalpy due to cooperative interactions when CITED2 is bound to the p300–HIF-1α complex relative to CITED2 binding to free p300. We observed a similar enthalpically favoured high affinity direct interaction with p300 with *K*_D_ = 10.6 nM for CITED2_218–256_ and 50.5 nM for CITED2ΔN (Fig. 2b, Fig. S1 (ESI†), Table 1). In line with the anisotropy data the two CITED2 sequences showed very different competition efficiencies (Fig. 2c and Table 1). The small positive Δ*g* (1.2 kcal mol^−1^) and the Δ*h* of 26 kcal mol^−1^ fitted for the competition of CITED2_218–256_ indicated an entropy-driven negative cooperativity with HIF-1α through the formation of a transient ternary intermediate. The competition of CITED2ΔN against p300–HIF-1α resulted in Δ*g* value greater than 4 kcal mol^−1^ (and Δ*h* = 0, Table 1.) indicating that the ternary complex formation is unlikely, resulting in the loss of the allosteric communication between the ligands.

**Table 1:** Thermodynamic parameters for direct binding and competition with p300–HIF-1α. ITC data were fitted globally to a model that included ternary intermediate formation (Fig. S20, ESI) using constrained *K*_D_ and Δ*H* for HIF-1α (Fig. S2, ESI). Δ*g* (and Δ*h*) represent the additional Gibbs energy (and enthalpy) due to cooperative interactions when CITED2 is bound to the p300–HIF-1α complex relative to CITED2 binding to free p300. Positive Δ*g* and Δ*h* values indicate negative cooperativity with an unfavourable contribution to enthalpy. 68% confidence intervals of the fitting are included in brackets

	Direct binding to p300			Cooperativity parameters, CITED2 binding to p300–HIF-1α		
	*K*_d_ (nM)	Δ*H* (kcal mol^−1^)	Δ*S* (cal mol^−1^ K^−1^)	Δ*g* (kcal mol^−1^)	Δ*h* (kcal mol^−1^)	Δ*s* (cal mol^−1^ K^−1^)
CITED2_218–256_	11 (8–13)	−14.3 (−14.1 to −14.6)	−10.0	1.1 (0.9–1.3)	26.3 (25.9–27.0)	82.7
CITED2ΔN	51 (45–57)	−10.6 (−10.8 to −10.5)	−1.0	>4	0	n.a.
1	19 (16–23)	−12.3 (−12.5 to −12.1)	−4.6	2.6 (2.3–2.9)	26.2 (24.4–29)	76.7
2	16 (13–20)	−11.1 (−10.9 to −11.3)	−0.4	1.4 (1.2–1.6)	24.3 (23.4–26.1)	74.1
3a	675 (595–814)	−4.6 (−4.8 to −4.4)	13.3	>4	0	n.a.
3b	98 (83–108)	−11.3 (−11.6 to −11.1)	−4.6	>4	0	n.a.
3c	29 (26–33)	−10.9 (−11.0 to −10.8)	−0.9	>4	0	n.a.
4	17 (12–23)	−10.3 (−10.6 to −10.1)	2.1	1.1 (0.9–1.4)	23.2 (22.1–24.14)	71.6

**Fig. 3:**
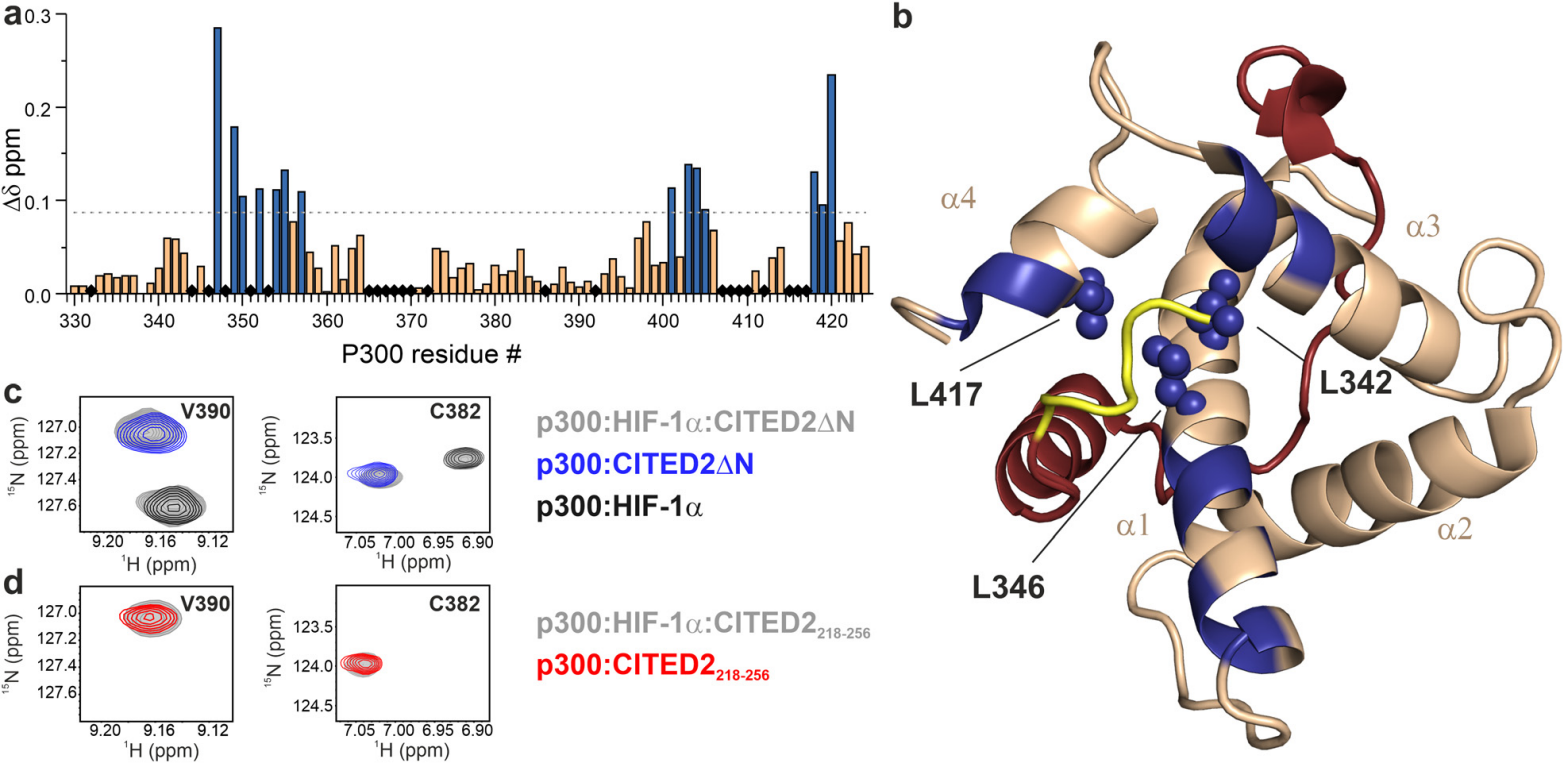
Structural comparison of CITED2_218–256_ and CITED2ΔN bound to p300 using NMR. (a) Weighted average ^1^H, ^15^N chemical shift difference between the p300–CITED2ΔN and p300–CITED2_218–256_ complex. Δ*δ* = [(Δ*δ*_H_)^2^ + (Δ*δ*_N_/5)^2^]^1/2^, residues above the significance level (Δ*δ* > 0.9 Δ*δ*_average_ + *σ*) highlighted blue. Black diamonds indicate unassigned or proline residues. (b) Residues with significant chemical shift differences mapped onto the p300–CITED2 structure and highlighted blue. Blue spheres represent core residues with significantly shifted CH_3_ resonances. See Fig. S5 (ESI†) for CH_3_ Δ*δ* values. (PDB: 1P4Q, p300 in wheat, CITED2 residues 224–259 in red, N-terminal residues 218–223 represented in yellow). (c) Overlayed ^1^H–^15^N resonances of representative p300 residues of samples containing p300:HIF-1α:CITED2ΔN (grey), p300:CITED2ΔN (blue) and p300:HIF-1α (black). (d) Overlayed ^1^H–^15^N resonances for the same p300 residues of samples containing p300:HIF-1α:CITED2_218–256_ (grey), p300:CITED2_218–256_ (red).

The effect of the N-terminal CITED2 residues on the conformation of p300 was characterised by solution NMR using ^13^C-, ^15^N-labelled p300_330–424_ (referred to as p300) in complex with the different CITED2 peptides. The calculated chemical shift perturbations using the assigned ^1^H and ^15^N resonances show that the two peptides induce different structural changes (Fig. 3 and Fig. S4, ESI†). The absence of the N-terminal CITED2 residues had a major effect on the amide resonances of p300 α1 residues 347–357, α3 residues 401–407, and α4 residues 418–420 (Fig. 3a and b). The observed changes were spatially close to the expected binding site of the N-terminal residues, suggesting that these residues interact with the protein surface. Since the core of p300 is tightly packed with hydrophobic amino acids, the CH_3_ chemical shifts of these residues can indicate the overall structural change. Significant differences in CH_3_ chemical shifts were observed for residues L342, L346 and L417 (Fig. S5, ESI†). These are located at the α1/α4 interface of p300 (Fig. 3b), suggesting that the presence of CITED2 N-terminal residues alters the relative orientation of these helices.

To investigate the reversibility of the competition, samples containing p300 with equimolar amounts of HIF-1α and CITED2_218–256_ or CITED2ΔN were prepared. For the p300:HIF-1α:CITED2ΔN sample, we observed chemical shifts that correspond to the binary complexes of p300–HIF-1α and p300–CITED2ΔN (Fig. 3c and Fig. S6, ESI†), indicating that both complexes are present in the solution. For p300:HIF-1α:CITED2_218–256_, only resonances corresponding to the CITED2-bound state were detected (Fig. 3d and Fig. S6, ESI†). These data strongly supported that the presence of the N-terminal residues is not only required for the negative cooperativity through the transient ternary complex formation, but also results in the conformational lock that renders the competition unidirectional. To rule out the effect of the slightly decreased affinity of CITED2ΔN on the competition results we repeated the FA and NMR experiments with CITED2_224–259_ which has a longer C terminus and a *K*_D_ similar to CITED2_218–256_^25^ (Table S1, ESI†). This modification, however, did not influence competition efficiency or unidirectionality (Fig. S7, ESI†), further confirming that the absence of the N-terminal tail is detrimental to the negative cooperativity with HIF-1α.

### Backbone perturbation strategy to probe the interplay between binding motifs

**Fig. 4:**
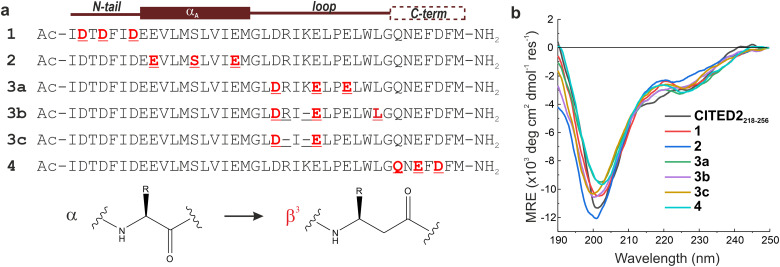
Sequences and overall structure of the β-amino acid modified CITED2 variants. (a) Sequences of modified CITED2 variants with binding motifs indicated above. α → β^3^ substitutions are highlighted in red, and deletions are represented by a line. (b) CD curves for CITED2 and variants in 20 mM Na-phosphate, 1 mM DTT, pH 7.4 at 20 μM concentration at room temperature.

The above analysis highlighted a determining role of the N-terminus; however, the literature results clearly indicate that a fine interplay between CITED2 segments are responsible for the negative regulation of HIF-1α. To probe cooperativity between the N-terminus and the rest of the binding motifs (α_A_ helix, loop and C-terminus), our goal was to induce site-directed structural changes in p300 by perturbing the bound conformation of CITED2 motif-by-motif. The induced structural changes are then correlated with the ability of the modified CITED2 to inhibit p300–HIF-1α. To separate the effect of direct binding affinity on the competition efficiency we set out to prepare native-like CITED2 variants, maintain the intrinsic disorder of the ligand, the original side-chain functionality, and the enthalpy-driven high-affinity interaction with p300. A backbone modification strategy fulfils all these requirements, for which we selected β^3^-amino acid replacements. These amino acids are frequently incorporated into helical secondary structures, and tolerated in loop regions, often resulting in overall fold and affinity comparable to the parent sequence.^39–43^ The extra methylene group in the backbone influences the local conformational preferences, thus structural adaptation of the target protein can be expected. Based on the above considerations, six CITED2 variants were synthesized (1–4, Fig. 4a) by modularly incorporating two to three β^3^-residues. To keep the perturbation effects of the modifications at a controlled level, our aim was to replace amino acids that have little contact with p300 in the bound state. This was supported by computational alanine scanning using BAlaS,^44,45^ and solvent-exposed amino acids whose side chains have only a small contribution to the binding free energy (<4 kJ mol^−1^) were selected for replacement or removal (Fig. S8, ESI†). To tune the distance between the αA helix and the C-terminus, three loop-region variants were prepared (3a–c), including deletions of amino acids to compensate for the backbone elongation caused by the additional methylene group of the β^3^-amino acids. To ensure that the modifications do not significantly affect the overall conformation of the CITED2 variants, circular dichroism (CD) measurements were carried out. CD spectra recorded for the free peptides 1–4 showed random coil conformation, with a slight helical content indicated by the minimum at 220 nm (Fig. 4b). This pointed toward that the presence of β^3^-amino acids did not induce unwanted folding, and the modified peptides retained their disordered nature.

**Fig. 5:**
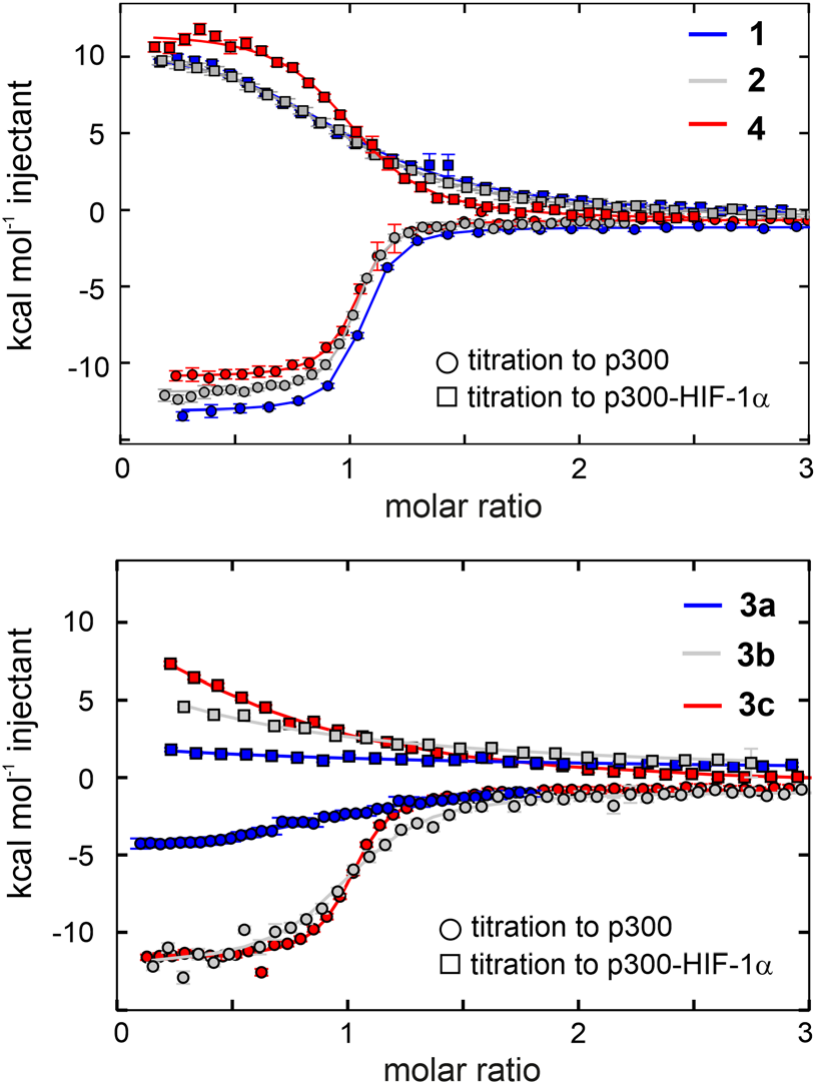
Direct binding to p300 and competition with p300-HIF-1α of the β-amino acid modified CITED2 variants. Direct binding (circles) and competition (squares) titrations were fitted globally to a model that involved the formation of ternary intermediate (Fig. S20, ESI†). Titrations were carried out in 40 mM sodium phosphate, pH 7.5 100 mM NaCl, 1 mM DTT buffer using 5 μM protein in the cell and 60 μM ligand in the syringe at 35 °C. Error bars represent raw data integration errors. Raw data and fitted thermograms are included in the ESI† (Fig. S9–S10).

The binding and competition efficiency of the CITED2 variants was examined using the same ITC method described above, from which the direct binding and cooperative parameters were derived (Fig. 5, Table 1; Fig. S9–S10, ESI†). Regarding their direct interaction with p300 sequences 1, 2, and 4 displayed a similar enthalpy-driven interaction with affinities (*K*_D_ in the range of 16–19 nM) comparable to the native CITED2 sequence (Table 1.). Modifications in the loop, however, resulted in more pronounced effects. Replacement in the conserved LPEL motif (3a) resulted in a 50-fold increase in *K*_D_ with an excessively different thermodynamic profile.

β-amino acid replacements were more tolerated when the conserved region was not directly involved. The thermodynamic signatures of the binding of 3b and 3c to p300 resembled more to the native CITED2 with *K*_D_ values 98 nM and 29 nM, respectively (Fig. 5 and Table 1). Overall, the less favourable binding enthalpy of the CITED2 variants pointed to loss of non-covalent interactions, which is compensated by decreased entropic cost of binding. This change may be attributed to the presence of the additional methylene groups, and can be the result of multiple contributing factors, such as a more favourable desolvation during folding,^46^ increased local flexibility of the bound structure^25^ or favourable displacement of surface waters.^47^ It should be noted that global conformational changes of p300 can also affect the detected thermodynamic profiles and cannot be ruled out based on these data.

The global analysis revealed that compounds 1, 2, and 4 maintained their ability to form the transient ternary intermediate indicated by the small positive Δ*g* values and exhibited negative cooperativity with HIF-1α (Table 1). The lower Δ*s* values of these variants point to decreased entropic stabilisation of the ternary intermediate, which can be the result of their less unfavourable direct binding entropy. Modification of the tail region (1) resulted in slightly higher Δ*g* (2.6 kcal mol^−1^) indicating that the transient intermediate is less stable, and this region is more sensitive to alterations, further underlining the role of the N-terminal tail in mediating effective competition. CITED2 variants containing loop modifications (3a–c) completely lost their ability to cooperate negatively with HIF-1α, even when the direct binding affinity was high (3c). Similar results were obtained using fluorescence anisotropy (Fig. S11 and Table S2, ESI†) where CITED2 variants 2 and 4 exhibited inhibitory efficiency comparable to the native sequence.

**Fig. 6:**
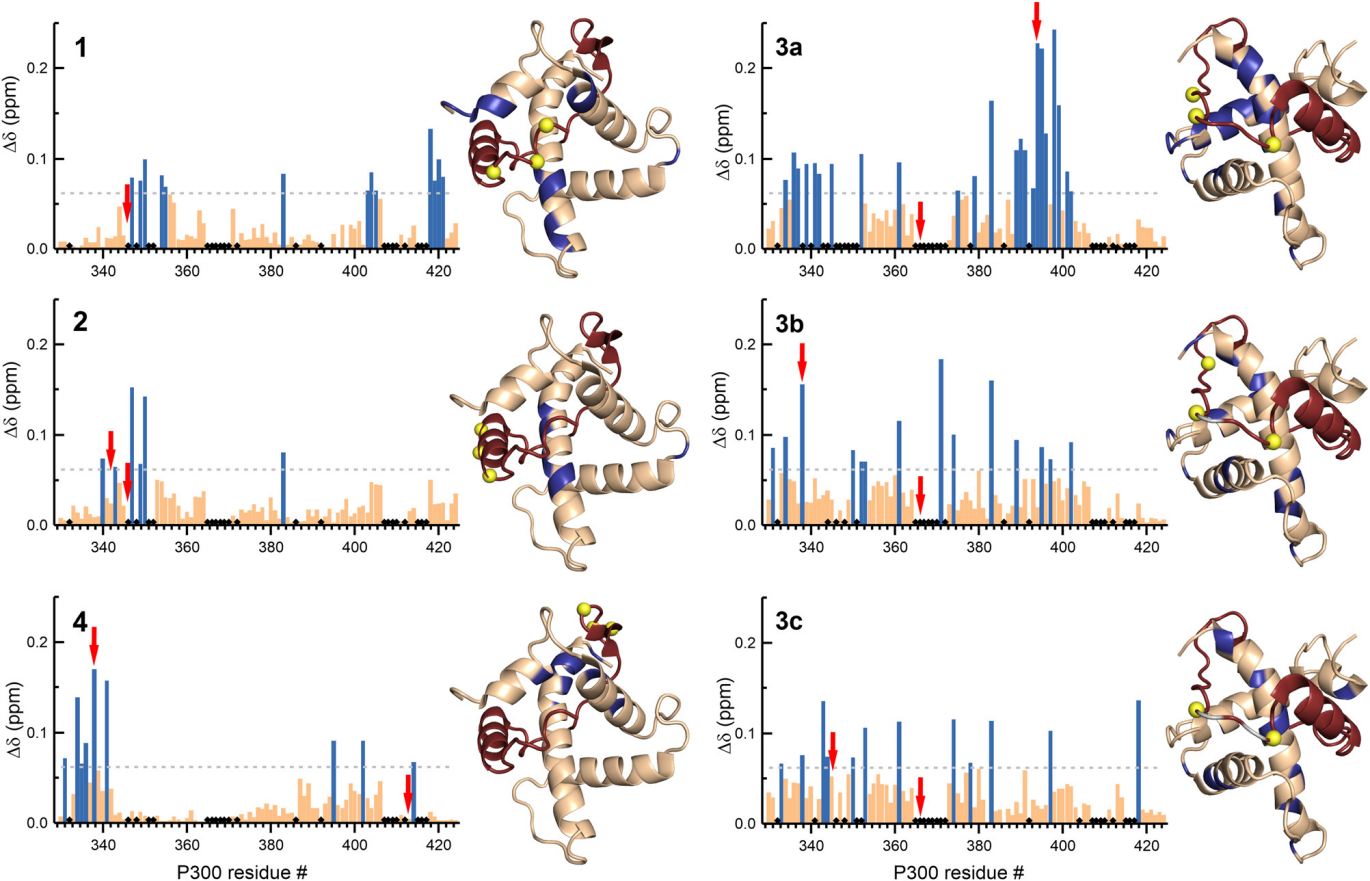
NMR chemical shift changes of p300 upon binding to the β-amino acid modified CITED2 variants. Weighted average ^1^H–^15^N chemical shift differences between the p300–CITED2 variants and p300–CITED2_218–256_ complexes. Δ*δ* = [(Δ*δ*_H_)^2^ + (Δ*δ*_N_/5)^2^]^1/2^. The significance level was determined using the average Δ*δ* and standard deviation for all the CITED2 variants, residues above the significance level (Δ*δ* > 0.9 Δ*δ*_average_ + σ) highlighted blue in the bar graph and mapped onto the p300–CITED2 structure, CITED2 residues in red, β^3^-amino acid modifications sites represented as yellow spheres, removed amino acids highlighted grey. Black diamonds on the bar graphs indicate unassigned or proline residues. Red arrows indicate residues for which significant methyl proton chemical shift differences were detected (Fig. S18–S19, ESI†).

To elucidate the effect of the β-amino acid modifications on the structure of p300, we recorded ^1^H–^15^N HSQC spectra using ^13^C-, ^15^N-labelled p300 in complex with all the peptides. The weighted average chemical shift differences were calculated relative to the native p300–CITED2_218–256_ complex (Fig. 6, Fig. S12–S17, ESI†). Generally, the observed chemical shift differences were low (Δ*δ* < 0.2 ppm), indicating that the overall folds of the complexes are similar, but the location of the observed changes was highly dependent on the modification site. β-amino acid replacements in the N-terminal tail of CITED2 (1) resulted in the change in the terminal regions of α1, α3, and α4 of p300, which was similar to what was observed for the p300–CITED2ΔN complex, further supporting the importance of the N-terminus in binding. The modified α_A_ helix (2) and C-terminal hydrophobic region (4) also had only local effects on the structure of p300, corresponding to the interaction sites of these binding motifs. Modifications in the loop region (3a–c), however, resulted in a more distributed effect, and significant chemical shift differences were observed for amino acids distant from the loop interaction site (Fig. 6). This suggested that the loop region is critical for connecting the recognition elements, and local modification of the loop might influence the placement of the other binding motifs.

The CH_3_ resonance changes corresponded well with the amide proton chemical shifts (Fig. 6 red arrows, Fig. S18–S19, ESI†). Methyl protons of L346 and L417 at the interface of α1 and α4 are mainly affected by the binding of 1, while L342 shifted the most upon interaction with 3a–c, resulting in chemical shift values close to the CITED2ΔN.

Overall, structural changes associated with modifications in the α_A_ helix and C-terminus of CITED2 can be tolerated, and the negative cooperativity with HIF-1α is retained. The N-terminus is more sensitive to backbone modifications, but to some extent retains the ability to act through the allosteric process. On the other hand, modifications in the loop of CITED2 are detrimental to the competition efficiency, which is accompanied by distributed structural changes, indicating strong cooperativity between these sequence motifs.

## Discussion

Understanding an effective competition mechanism to which several factors contribute, such as the high binding affinity of the competing ligands, the cooperativity between the binding motifs, and allosteric structural changes, can be challenging. Several studies investigated the molecular details by which CITED2 disrupts the p300/CBP–HIF-1α complex,^22–29,33^ often highlighting different key determinants for the unidirectional competition. Here, we clarify the role of a crucial sequence part of CITED2 that is necessary for negative cooperativity with HIF-1α. We also introduce a strategy with which the interplay between binding motifs and their individual contribution to allostery can be efficiently probed.

The two sequence variants, CITED2_218–256_ and CITED2_224–256_ (CITED2ΔN) display an enthalpy-driven high-affinity interaction with p300, but their ability to displace HIF-1α significantly differs (Fig. 2 and Table 1). The presence of N-terminal residues (CITED2 218–223) is required for negative cooperativity through the formation of transient ternary complex (Fig. 2). Our NMR data indicate that these residues interact with p300 and induce conformational changes that affect the α1/α4 interface (Fig. 3). This is in line with the previously reported mechanism but reveals a different effector. It has been shown that the conformational change of the α4 helix of p300/CBP mediates the negative cooperativity between CITED2 α_A_ and HIF-1α α_C_.^24^ Still, our data indicate that without the N-terminal tail, the competition is reversible (Fig. 3c). Therefore, N-terminal residues are essential to lock the conformation into the CITED2-bound state. Although it has not been explicitly stated, an indication of such a mechanism could be seen from the NMR data of a fusion peptide comprising CITED2 residues 216–246 connected to HIF-1α residues 796–826.^24^ An L822 to alanine mutation in the fusion peptide, which impairs the binding of the HIF-1α α_C_ helix, resulted in significant chemical shift changes of residues corresponding to the CITED2 N-terminal tail, suggesting that these residues are involved in the displacement of the HIF-1α α_C_ helix.

To probe the interplay between the N-terminal tail with all the binding motifs, we introduce a strategy that maintains the native properties of CITED2 but allows binding site-targeted investigation of individual sequence parts. Using β-amino acid replacements in a native ligand is a well-known approach for designing peptidomimetics with improved properties.^40,42,43,48^ Helical and loop structures containing β^3^-amino acids closely resemble the native fold and reproduce the binding contacts with the target protein.^39,41,49,50^ We expected that native-like CITED2 variants could be obtained with α to β^3^ amino acid replacements, to which p300 would respond with binding-site dependent structural adaptation. Using a motif-by-motif screen, we prepared CITED2 variants that bind with high affinity to p300 and show similar thermodynamic fingerprints (Fig. 5) that helped us to probe the correlation between structural adaptation and competition efficiency.

Backbone modifications of the α_A_ helix (2) and the hydrophobic C terminus (4) of CITED2 cause local structural changes in p300 that are accompanied by retained negative cooperativity with HIF-1α (Fig. 5 and 6, Table 1). This indicates that these motifs are not directly involved in the structural change that renders the competition unidirectional. Taking into account that the contribution of the N-terminal tail to p300 binding affinity is not significant (Table 1), it suggests that the role of the simultaneously bound α_A_ and C terminus is to provide high affinity, thus a strong tether for the tail to bind. This is in line with the reported sequence truncation data. Modifications that decrease the p300/CBP binding affinity of CITED2, such as removing the hydrophobic C-terminus,^23^ can lead to a weakened tether for the N-terminal tail; the conformational lock is lost, and the competition becomes reversible. Molecular dynamic simulations suggested that the effector in the allosteric process is the highly conserved charged residues of the CITED2 helix immediately after the N-terminal tail.^29^ In conjunction with our data, the role of this charged patch is more likely to contribute to the strong tether for the N-terminal tail.

The N-terminal tail is more sensitive to backbone modifications. The location of structural changes in p300 upon binding to the α/β-modified N-terminus matches with those that we observed for CITED2ΔN, but the changes are less pronounced (Fig. 6, 3b). This is in good agreement with the ability of compound 1 to partially retain its function in forming the transient intermediate and acting as the allosteric effector. This also holds promise that with careful design the function of this key sequence part can be mimicked.

On the other hand, the loop region of CITED2 is highly sensitive to modifications leading to decreased p300-binding affinity (3a–b), and loss of competition efficiency even when binding parameters closer to the native CITED2 are retained (3c, Fig. 5). Several studies highlighted that CITED2 binding motifs act synergistically in which the loop region is functionally important.^23,26^ Our data indicate a similar role; the loss in competition efficiency accompanied by the distributed structural changes in p300 point toward that all binding motifs are affected by the modified loop that leads to weak cooperativity or the misplacement of important binding motifs.

In conjunction with previous studies, our data provide a more detailed picture of the molecular mechanism of the competition between CITED2 and HIF-1α. CITED2_218–256_ is the minimal sequence required for the irreversible competition with HIF-1α, in which the concerted action of the binding motifs that leads to the conformational lock provided by the N-terminal tail is crucial. Several groups have shown that inhibiting p300/CBP–HIF-1α is a promising strategy to influence pathological conditions related to hypoxia.^8,11,34,51–54^ An inhibitory ligand that exploits this unidirectional competition with HIF-1α would be highly advantageous. Our data provide insights for modification sites developing such ligand.

Sensitive regulation of the hypoxic response essentially relies on the intrinsic disorder of CITED2 and HIF-1α; since IDPs are prevalent in eukaryotic cells, determining crucial factors that contribute to their regulatory mechanisms would provide insight into their function. IDPs can form specific interactions with multiple protein partners, often through independent binding motifs.^1–3,55^ The precise regulation mediated by IDPs is often facilitated by their ability to engage in transient complexes and compete with partially shared interaction sites, resulting in allosteric changes.^56–59^ Such complexity often hinders understanding of the underlying processes in molecular detail. The site-directed conformational perturbation strategy presented in this work provides a general approach to gain mechanistic insights for IDP-mediated interactions with multiple, cooperatively acting binding motifs and inform inhibitor design for interactions of therapeutic relevance.

## Author contributions

V. L. P., Z. H., and T. A. M. conceived the project and designed the experiments. V. L. P., I. P., and Z. H. performed experiments and data analysis. Z. H. wrote the manuscript with contributions from all authors.

## Conflicts of interest

There are no conflicts to declare.

## Supplementary Material

CB-005-D4CB00066H-s001
