# Supplementary material for: Site-directed allostery perturbation to probe the negative regulation of hypoxia inducible factor-1α
Source: RSC Chem Biol. 2024 May 30;5(8):711–20. doi: 10.1039/d4cb00066h (PMC11289882; doi:10.1039/d4cb00066h)
Supplement: CB-005-D4CB00066H-s001 [file CB-005-D4CB00066H-s001.pdf]

Electronic Supplementary Material (ESI) for RSC Chemical Biology.
This journal is © The Royal Society of Chemistry 2024

Supporting Information for

Site-directed allostery perturbation to probe the negative regulation of hypoxia inducible factor-1α

Vencel L. Petrovicz[1], István Pasztuhov[1], Tamás A. Martinek[1,2*], Zsófia Hegedüs[1*]

[1]University of Szeged, Department of Medical Chemistry, 8 Dóm tér, Szeged, 6720 Hungary

[2]HUN-REN SZTE Biomimetic Systems Research Group, 8 Dóm tér, Szeged, 6720 Hungary

Corresponding authors: Tamás A. Martinek, Zsófia Hegedüs

martinek.tamas@med.u-szeged.hu; hegedus.zsofia@med.u-szeged.hu

Table S1. Binding affinities of HIF-1α and CITED2 variants. 3
Fig. S1 ITC titrations of CITED2 variants 4
Fig. S2 ITC titrations of HIF-1α$_{776-826}$ binding to p300 4
Fig. S3 Fluorescence anisotropy competition of HIF-1α with Flu-HIF-1α 5
Fig. S4 NMR spectra comparing p300-bound CITED2 sequences 6
Fig. S5 p300 CH_3 chemical shift changes in complex with different CITED2 peptides 7
Fig. S6 ^{1}H-^{15}N HSQC spectra of p300 with equimolar mixtures of HIF-1α and CITED2 8
Fig. S7 Comparison of CITED2$_{224-259}$ with CITED2$_{218-256}$ 9
Fig. S8 Selected replacement positions in CITED2 using computational alanine scan........ 10
Fig. S9 ITC thermograms for **1-4** binding to p300........ 11
Fig. S10 Competition ITC titrations for compounds **1-4**........ 12
Fig. S11 Fluorescence anisotropy competition titrations of **1-4**. 13
Table S2. Apparent competition K_D values fitted for the competition fluorescence anisotropy titrations 13
Fig. S12 ^{1}H-^{15}N HSQC spectra of CITED2$_{218-256}$ and **1** in complex with p300........ 14
Fig. S13 ^{1}H-^{15}N HSQC spectra of CITED2$_{218-256}$ and **2** in complex with p300. 15
Fig. S14 ^{1}H-^{15}N HSQC spectra of CITED2$_{218-256}$ and **3a** in complex with p300........ 16
Fig. S15 ^{1}H-^{15}N HSQC spectra of CITED2$_{218-256}$ and **3b** in complex with p300. 17
Fig. S16 ^{1}H-^{15}N HSQC spectra of CITED2$_{218-256}$ and **3c** in complex with p300........ 18
Fig. S17 ^{1}H-^{15}N HSQC spectra of CITED2$_{218-256}$ and **4** in complex with p300. 19
Fig. S18 Weighted average chemical shift differences of p300 CH_3 resonances........ 20
Fig. S19 p300 CH_3 chemical shifts affected by CITED2 modifications 21
Methods 22
Protein expression and purification **22**
Peptide synthesis and purification **22**
Circular dichroism **24**
Isothermal titration calorimetry **24**
Fig. S20 Parametrized diagram of the model used to fit cooperative parameters for the CITED2, HIF-1α, p300 system. 25
Fluorescence anisotropy **26**
NMR measurements........ **27**
Characterization data 29
Fig. S21 p300 protein expression 29
HPLC chromatograms and mass spectra of the synthesized peptides 30
Table S3. Amide proton chemical shifts of p300 in complex with CITED2 variants 41
Table S4. Assigned CH_3 chemical shifts of p300 in complex with the different CITED2 variants 44
References 45

Table S1. Binding affinities of HIF-1α and CITED2 variants.
Previously reported p300/CBP binding affinity and apparent competition K_D values for different CITED2 and HIF-1α sequences.

	Direct binding to p300/CBP	Competition with HIF-1α	Reference
HIF-1α$_{776-826}$	10 ± 2 nM [1,2a]	10 ± 1 nM[2a]	1
HIF-1α$_{776-826}$	52 (49–56) nM[3]	n.a.	2
HIF-1α$_{786-826}$	43 (38–48) nM[3]	n.a.	2
CITED2$_{216-269}$	9 ± 2 nM[1]<br>10 ± 1 nM[2b]	0.2 ± 0.1 nM[2a]	1
CITED2$_{216-269}$	26 (21–33) nM[3]	1.1 (0.9–1.4) nM[4]	2
CITED2$_{224-259}$	9 (7–12) nM[3]	12 (11–13) nM[4]	2
CITED2$_{216-246}$	5 ± 1 μM[2b]	210 ± 20 nM[2a]	3
CITED2$_{216-248}$	303 (230–397) nM[3]	47 (40–56) nM[4]	2
CITED2$_{216-246}$ with LPEL motif mutated to APAA	2.5 ± 0.2[2b] μM	50 ± 10 nM[2a]	4

[1] K_D Bio-layer interferometry
[2a] $K_{D,\ apparent}$ Fluorescence anisotropy titration against CBP bound Alexa594-HIF-1α or [2b]Alexa594-CITED2
[3] K_D ITC, 68% confidence intervals in brackets
[4] $K_{D,\ apparent}$ ITC titration against p300-HIF-1α complex, 68% confidence intervals in brackets

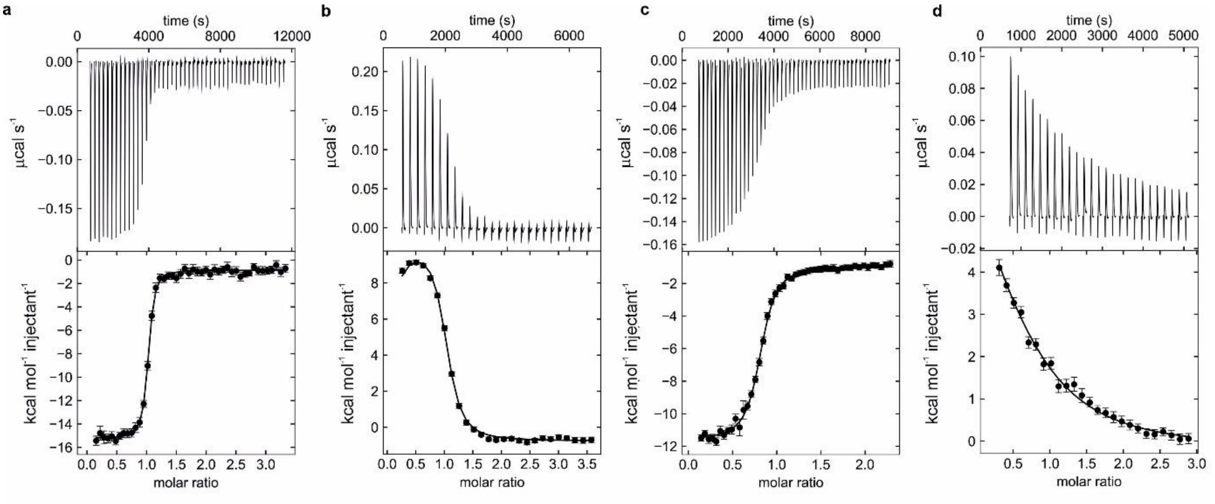

Fig. S1 ITC titrations of CITED2 variants

a Raw ITC data and fitted thermogram for direct binding of CITED2$_{218-256}$ to p300$_{330-420}$ **b** Competition ITC titration of CITED2$_{218-256}$ to preformed p300–HIF-1α$_{776-826}$ complex. **c** Raw ITC data and fitted thermogram for direct binding of CITED2ΔN to p300$_{330-420}$ **d** Competition ITC titration of CITED2ΔN to preformed p300–HIF-1α$_{776-826}$ complex. The data were fitted to a competition model including parameters for ternary intermediate formation depicted in Fig. S20 using restrained K_D = 50 ± 8 nM and ΔH = -23.3 ± 2.6 kcal/mol for HIF-1α (determined beforehand, Fig. S2). Error bars represent estimated integration errors. Titrations were performed at 35°C, in 40 mM Na-phosphate, 100 mM NaCl, 1 mM DTT pH 7.4 buffer.

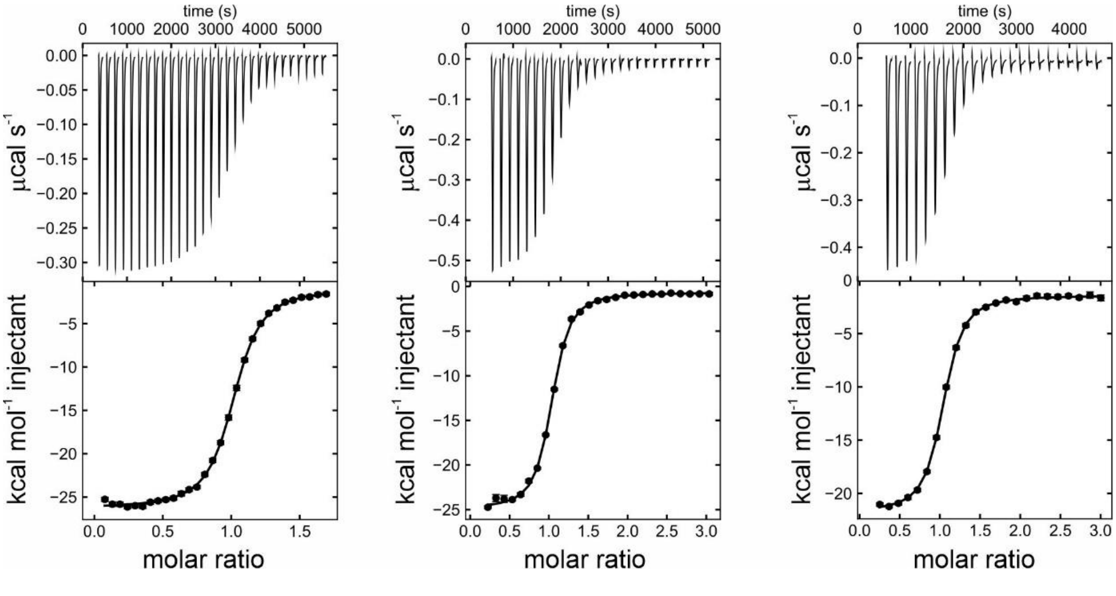

Fig. S2 ITC titrations of HIF-1α$_{776-826}$ binding to p300

Raw ITC data and fitted thermograms for HIF-1α$_{776-826}$ binding to p300$_{330-420}$, in 40 mM Na-phosphate, 100 mM NaCl, 1 mM DTT pH 7.4 buffer using 5 µM protein in cell and 60 µM peptide in the syringe at 35°C. Average K_D of the three independent measurements: 50 ± 8 nM, average ΔH = -23.3 ± 2.6 kcal/mol.

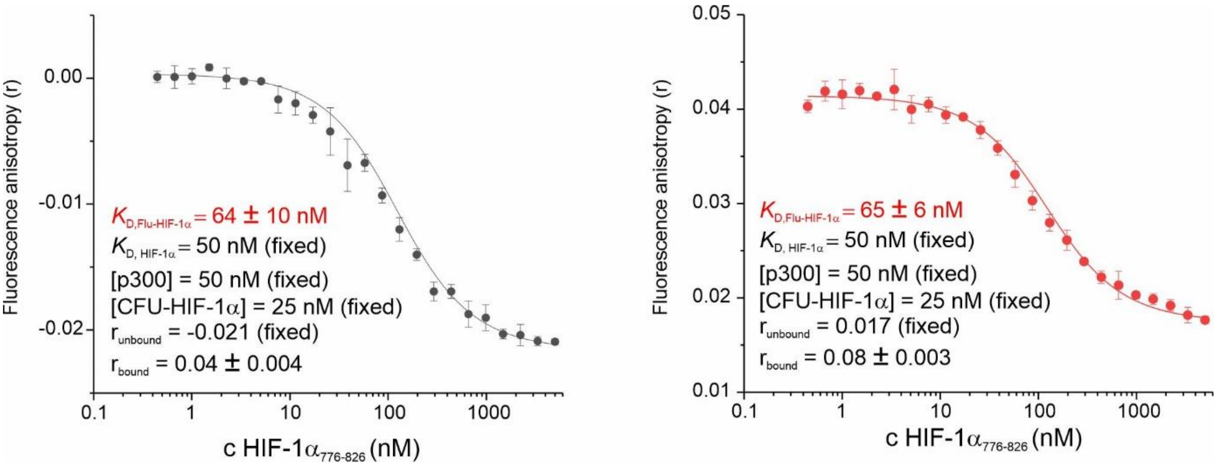

Fig. S3 Fluorescence anisotropy competition of HIF-1α with Flu-HIF-1α

Fluorescence anisotropy (FA) competition titrations were used to determine K_D for the fluorescein-labeled HIF-1α$_{786-826}$ (Flu-HIF-1α). Measurements were carried out in 40 mM Na-phosphate, 100 mM NaCl, 1 mM DTT, 0.01% Triton-X, pH 7.4 buffer, using 50 nM p300$_{330-420}$, 25 nM Flu-HIF-1α as tracer and HIF-1α$_{776-826}$ as the competitor. Plates were read after a 10-minute incubation at 35°C. Curves were fitted to a competition model with the listed parameters using equations 10-19 (see Methods) in Origin Pro. K_D for the competitor HIF-1α$_{776-826}$ (50 ± 8 nM) was determined by ITC (Fig. S2).

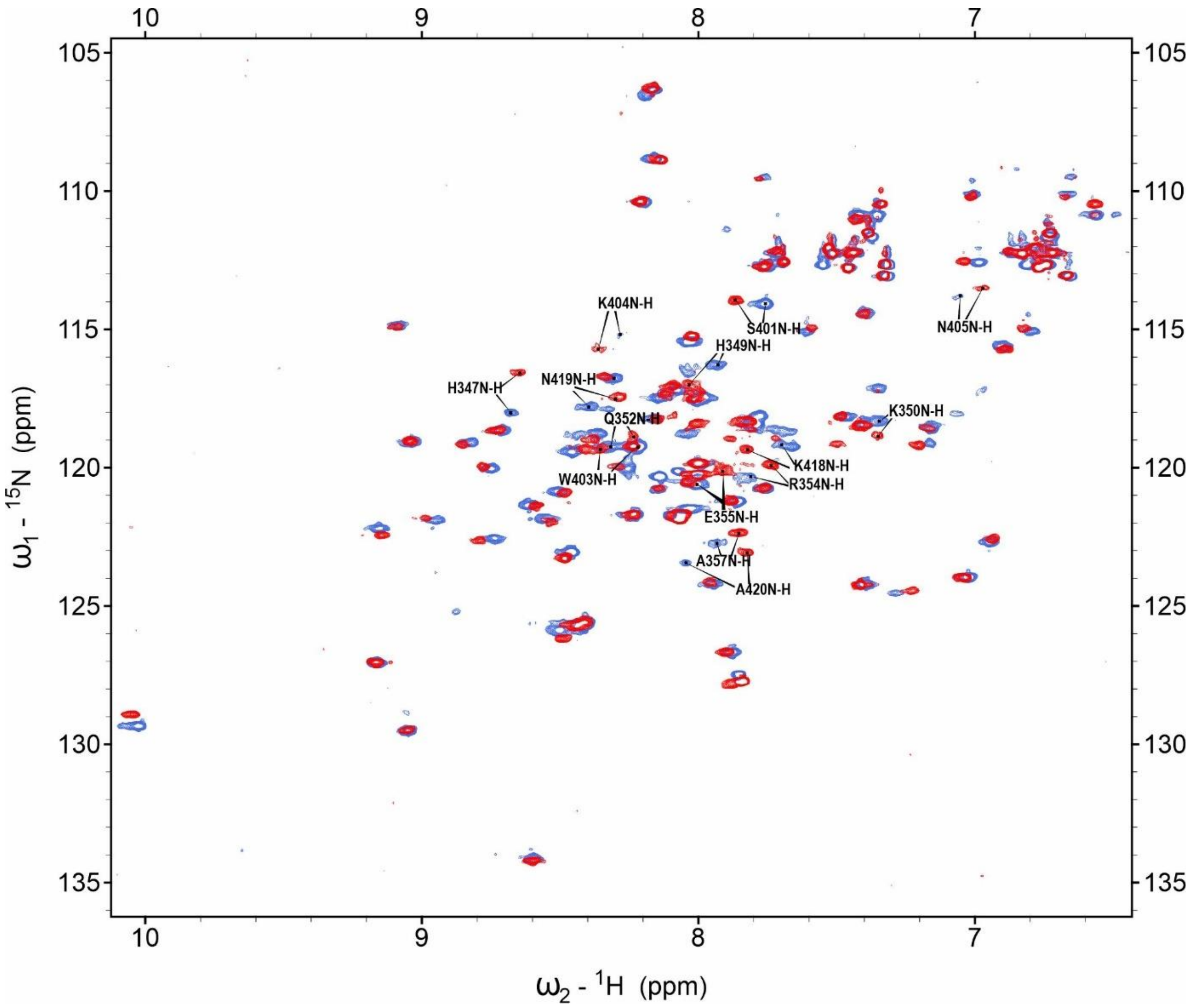

Fig. S4 NMR spectra comparing p300-bound CITED2 sequences

Overlayed ^{1}H-^{15}N HSQC spectra of **CITED2$_{218\text{-}256}$** (red) and **CITED2ΔN** (blue) in complex with p300. Spectra were recorded at 298K in 10mM Tris pH 6.9, 50 mM NaCl, 1 mM DTT, 0.02% NaN_3, and 10% D_2O. Weighted average chemical shift changes were calculated using the following formula: $\Delta\delta = [(\Delta\delta_H)^2 + (\Delta\delta_N/5)^2]^{1/2}$; $\Delta\delta_{average}$ = 0.040 ppm, σ = 0.051 ppm; p300 residues that shifted significantly ($\Delta\delta > 0.9\ \Delta\delta_{average} + \sigma$) are labeled.

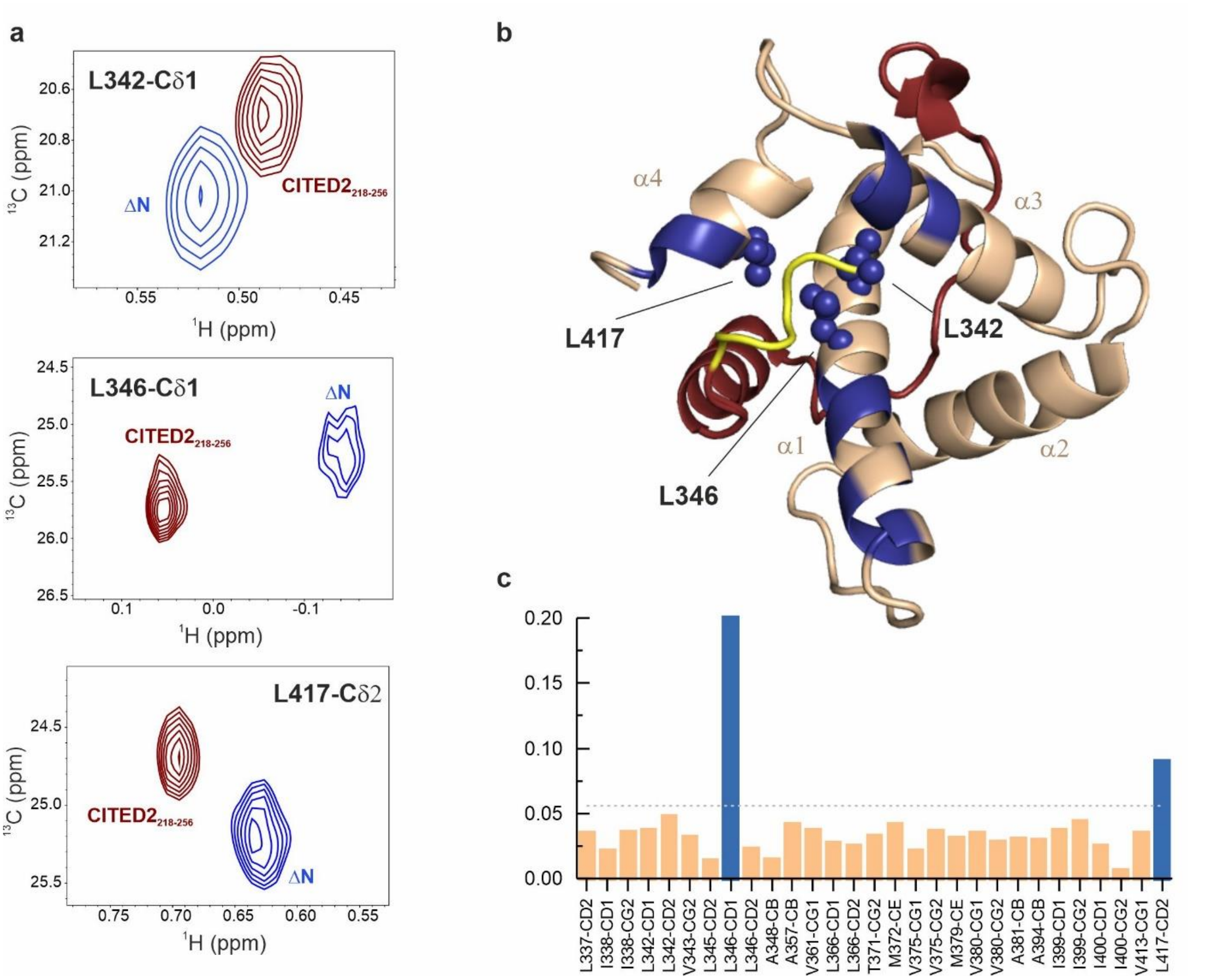

Fig. S5 p300 CH_3 chemical shift changes in complex with different CITED2 peptides

a Overlayed ^1H-^{13}C HSQC spectra of representative p300 CH_3 resonances in complex with CITED2ΔN (blue) and $CITED2_{218\text{-}256}$ (red) **b** Significant amide and methyl chemical shift differences between CITED2ΔN–p300 and p300–$CITED2_{218\text{-}256}$ complex mapped onto the CITED2–p300 structure (p300 wheat, CITED2 red, amino acids with significant methyl proton shifts represented as spheres, N-terminal residues 218-224 represented yellow). **c** Weighted average p300 CH_3 chemical shifts for the CITED2ΔN–p300 complex relative to the chemical shifts of the p300–$CITED2_{218\text{-}256}$ complex ($\Delta\delta = [(\Delta\delta_H)^2 + (\Delta\delta_C/5)^2]^{1/2}$). p300 residues that shifted significantly ($\Delta\delta > 0.9\ \Delta\delta_{average} + \sigma$) are highlighted blue.

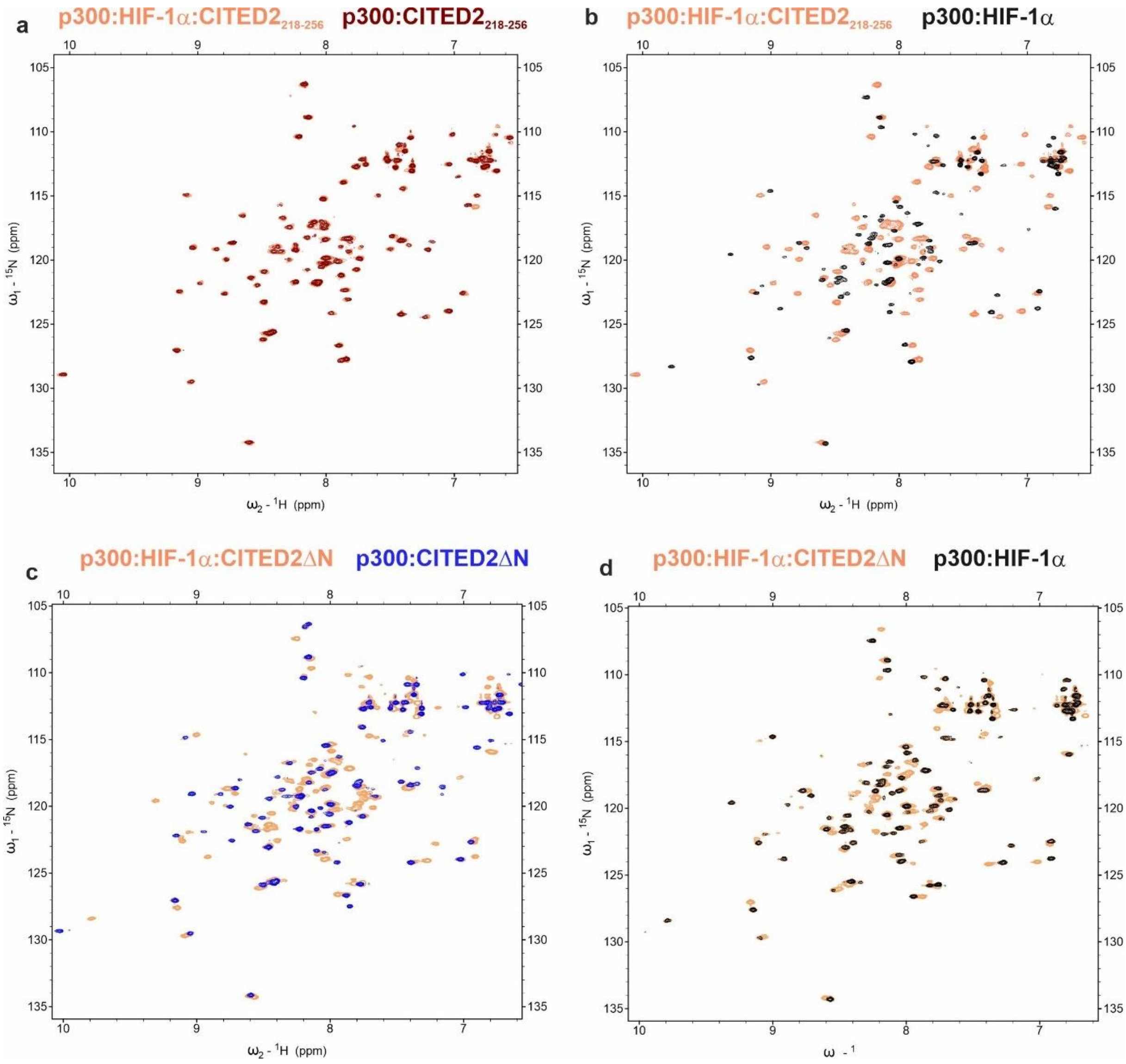

Fig. S6 ^{1}H-^{15}N HSQC spectra of p300 with equimolar mixtures of HIF-1α and CITED2

a-b ^{1}H-^{15}N HSQC spectra of p300 containing equimolar CITED2$_{218-256}$ and HIF-1α$_{776-826}$ (coral) overlayed with the HSQC spectra of p300–CITED2$_{218-256}$ complex (**a**, red) or p300–HIF-1α$_{776-826}$ complex (**b**, black). **c-d** ^{1}H-^{15}N HSQC spectra of p300 containing equimolar CITED2ΔN and HIF-1α$_{776-826}$ (coral) overlayed with the HSQC spectra of p300–CITED2ΔN complex (**c**, red) or p300–HIF-1α$_{776-826}$ complex (**d**, black).

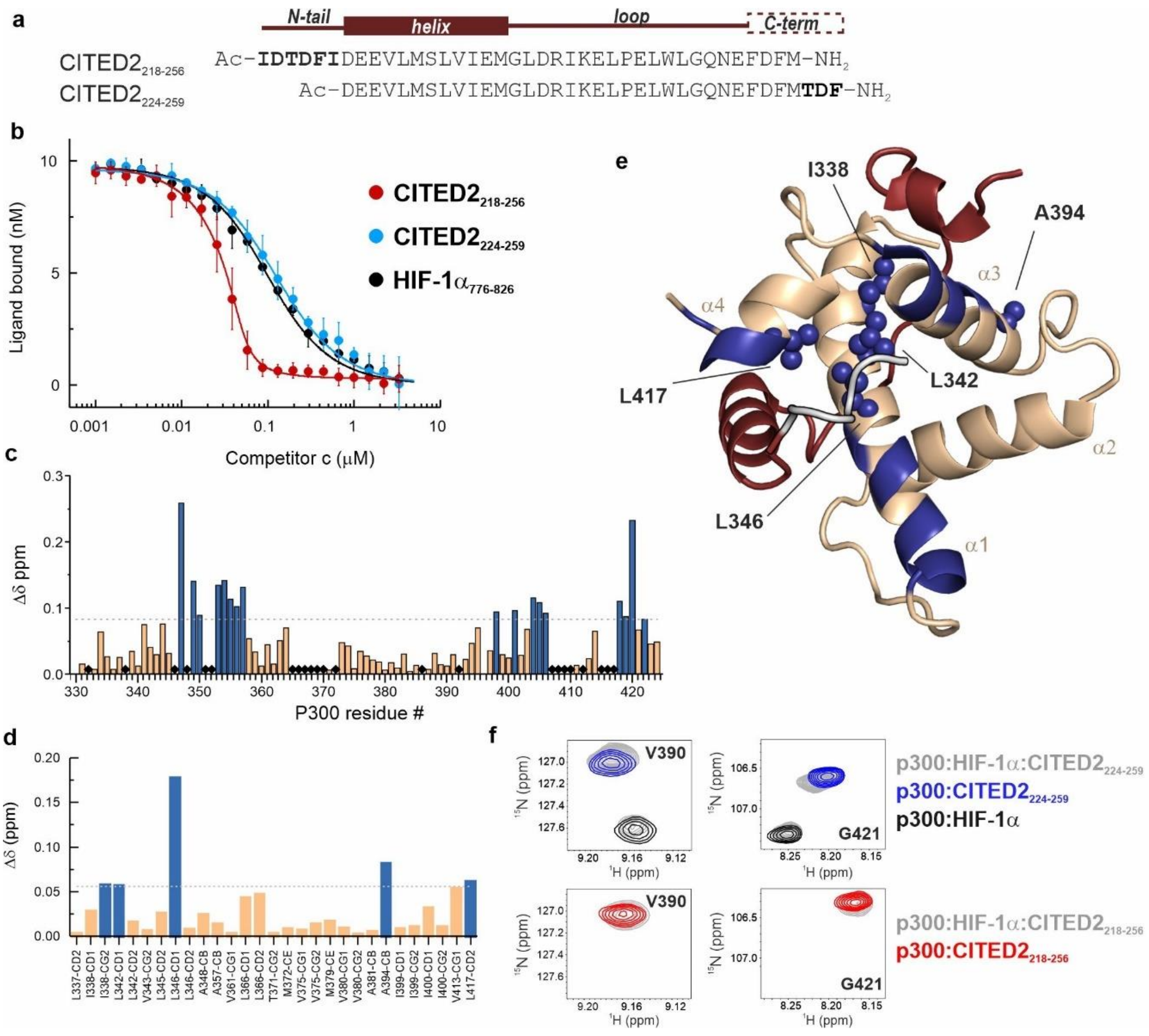

Fig. S7 Comparison of CITED2$_{224\text{-}259}$ with CITED2$_{218\text{-}256}$ **a** Sequences of CITED2 peptides with their binding motifs indicated above. **b** Fluorescence anisotropy competition titrations against fluorescein-labeled HIF-1α$_{786\text{-}826}$ (Flu-HIF-1α) in complex with p300 using 50 nM protein and 25 nM tracer concentration, curves were fitted to a competition model using fixed K_D value for Flu-HIF-1α (K_D = 64 nM) determined beforehand (Fig. S3). **c** Weighted average ^{1}H, ^{15}N chemical shift difference between the p300–CITED2$_{224\text{-}259}$ and p300–CITED2$_{218\text{-}256}$ complex. $\Delta\delta = [(\Delta\delta_H)^2 + (\Delta\delta_N/5)^2]^{1/2}$, residues above the significance level ($\Delta\delta > 0.9\ \Delta\delta_{average} + \sigma$) highlighted blue. Black diamonds indicate unassigned or proline residues. **d** Weighted average p300 CH_3 chemical shifts bound to CITED2$_{224\text{-}259}$ relative to the chemical shifts of the p300–CITED2$_{218\text{-}256}$ complex ($\Delta\delta = [(\Delta\delta_H)^2 + (\Delta\delta_C/5)^2]^{1/2}$). p300 residues that shifted significantly ($\Delta\delta > 0.9\ \Delta\delta_{average} + \sigma$) are highlighted blue. **e** Significant amide and methyl chemical shift changes (blue) mapped onto the CITED2–p300 structure (p300 wheat, CITED2 red, amino acids with significant methyl proton shifts represented as spheres, N terminal residues 218-224 represented grey). **f** Overlayed ^{1}H-^{15}N resonances of representative p300 residues of samples containing p300:HIF-1α:CITED2$_{224\text{-}259}$ (grey), p300:CITED2$_{224\text{-}259}$ (blue) and p300:HIF-1α (black). Overlayed ^{1}H-^{15}N resonances for the same p300 residues of samples containing p300:HIF-1α:CITED2$_{218\text{-}256}$ (grey), p300:CITED2$_{218\text{-}256}$ (red).

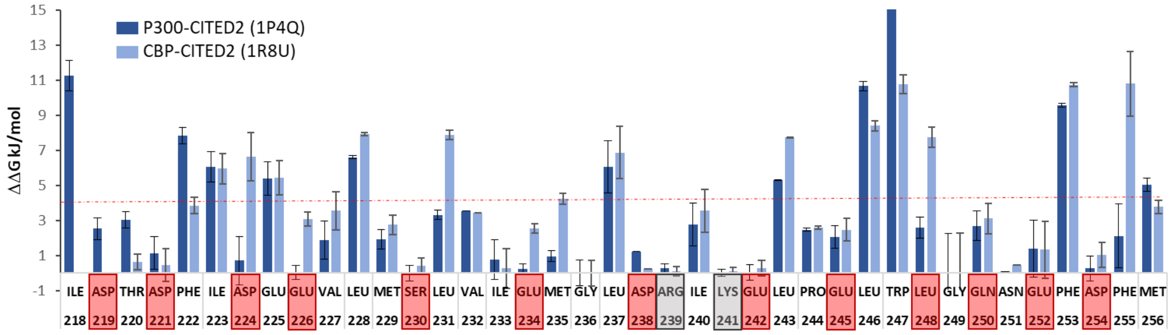

Fig. S8 Selected replacement positions in CITED2 using computational alanine scan.

Computational alanine scan results of p300–CITED2 and CBP–CITED2 complex using BAlaS.[5] ΔΔG values above 4 kJ/mol (indicated by a red dashed line) are considered significant. Error bars represent the standard deviation of the ΔΔG values computed for each structure in the NMR ensemble. Selected β^3-amino acid replacement positions in the CITED2 sequence are indicated by red squares; amino acid deletion positions are indicated by grey squares.

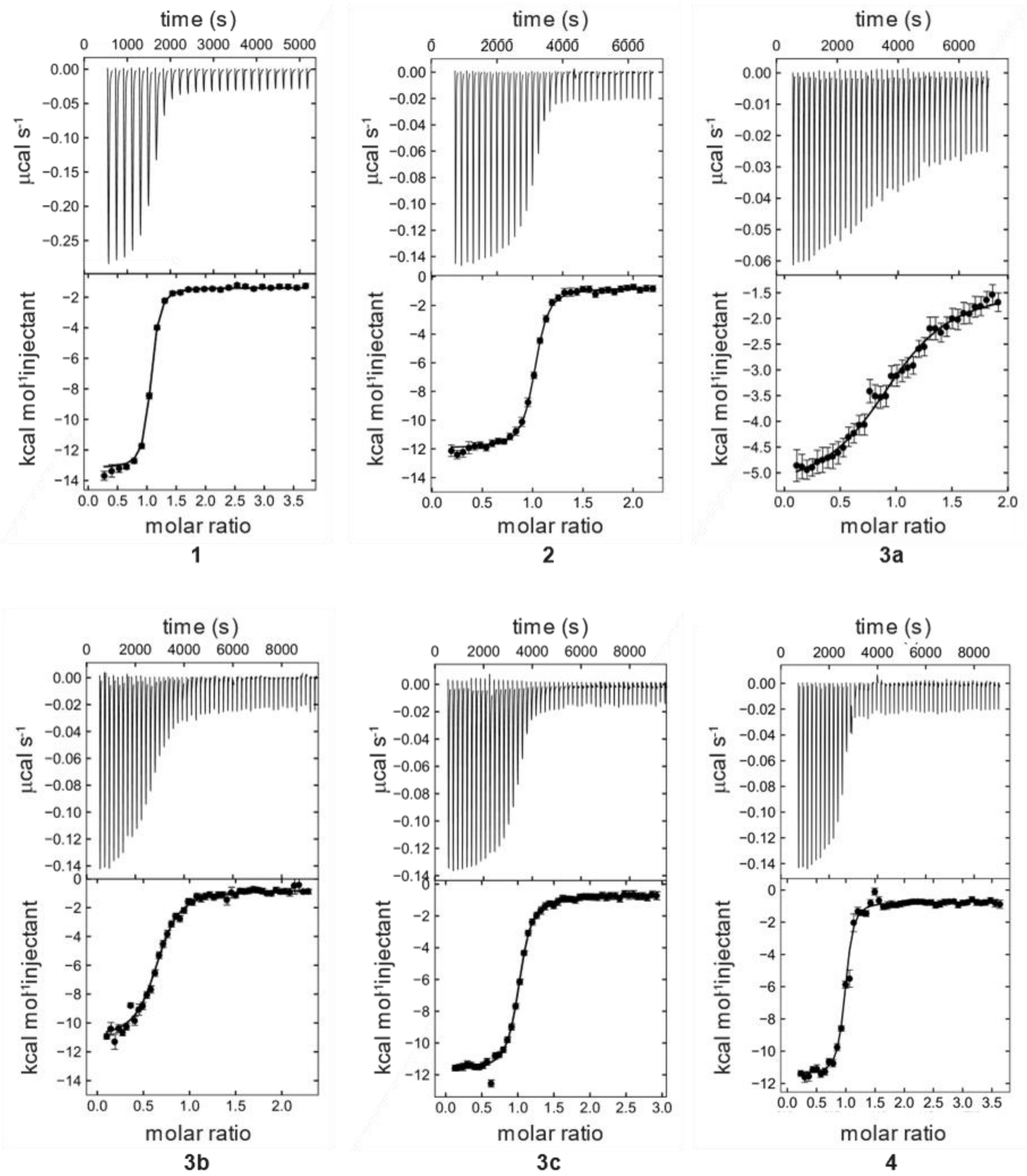

Fig. S9 ITC thermograms for 1-4 binding to p300

Raw ITC data and fitted thermograms for **1-4** titrated to p300$_{330-420}$ (35 °C; 40 mM Na-phosphate; 150 mM NaCl; 1 mM DTT; pH 7.4) using 5 μM p300 in the cell and 60 μM CITED2 variant in the syringe; error bars represent estimated integration errors. Data fitted globally together with the competition titrations (Fig S10.) to a competition model including the ternary intermediate formation (Fig. S20.) using constrained K_D and ΔH for HIF-1α (determined beforehand Fig. S2).

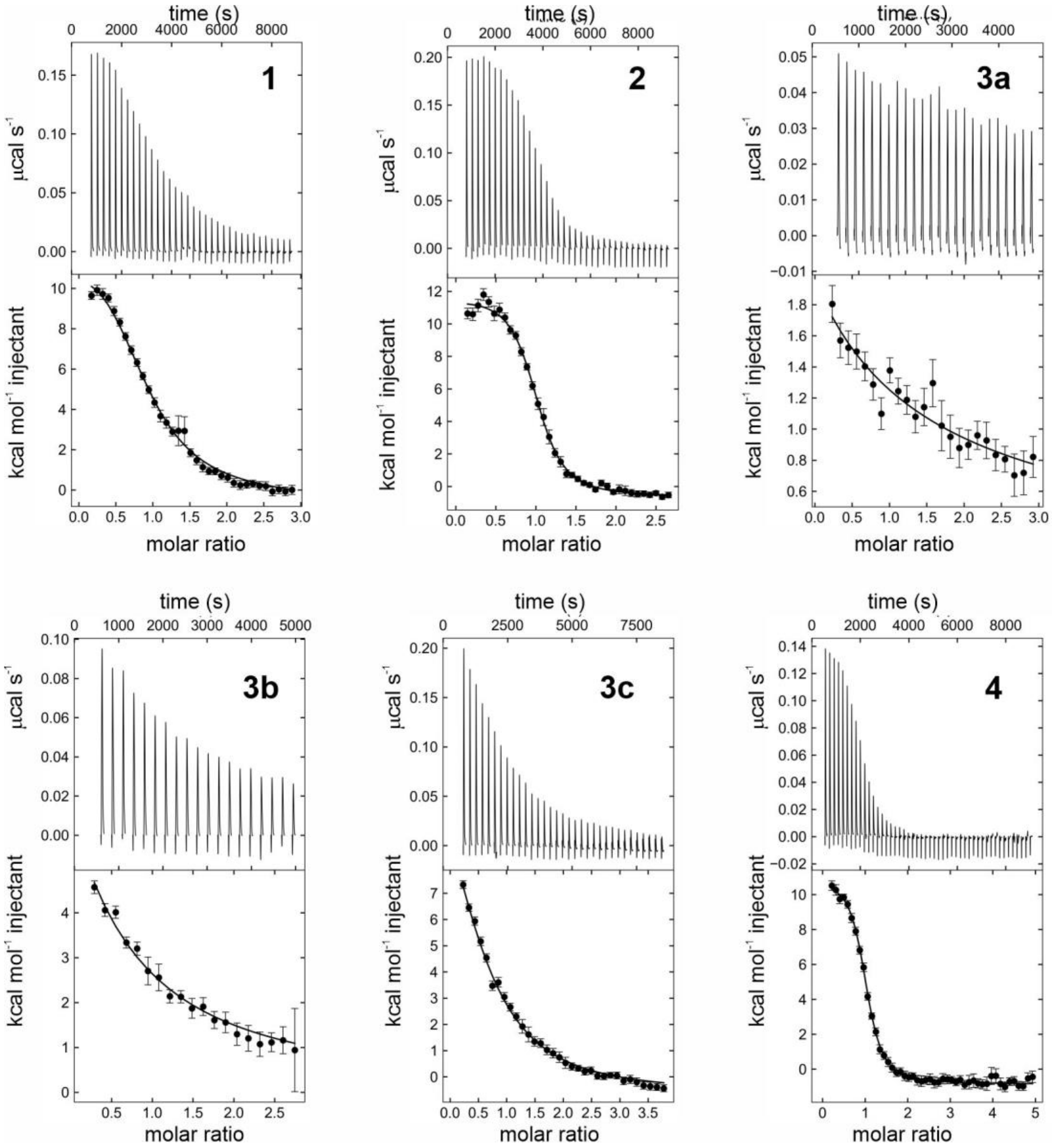

Fig. S10 **Competition ITC titrations for compounds 1-4**

Competition ITC experiments with raw data (upper) and fitted thermogram (lower). CITED2 variants were titrated to preformed p300–HIF-1α complex at 35 °C in 40 mM Na-phosphate; 150 mM NaCl; 1 mM DTT; pH 7.4. Data fitted globally together with the direct titrations (Fig S9.) to a competition model including the ternary intermediate formation (Fig. S20.) using constrained K_D and ΔH for HIF-1α (determined beforehand Fig. S2).

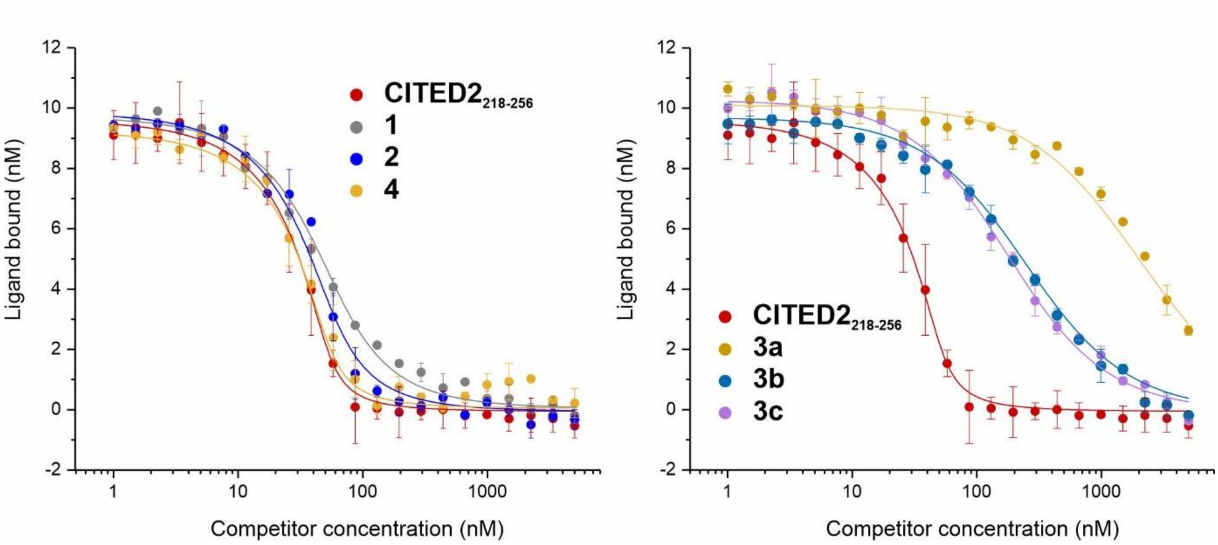

Fig. S11 Fluorescence anisotropy competition titrations of 1-4.
Measurements were carried out in 40 mM Na-phosphate, 100 mM NaCl, 1 mM DTT, 0.01% Triton-X, pH 7.4 buffer. CITED2 and variants were serially diluted on the plate using 2/3 dilution series, with 5 μM as the highest concentration to which 50 nM $p300_{330\text{-}420}$ and 25 nM Flu-HIF-1α were added. Plates were read after a 10-minute incubation at 35°C. Curves were fitted to a competition model using equations 10-19 (See Methods) in Origin Pro using fixed parameters for protein, tracer concentration, and K_D = 64 nM for Flu-HIF-1α (determined beforehand, Fig. S2).

Table S2. Apparent competition K_D values fitted for the competition fluorescence anisotropy titrations (Fig. S11.) Curves were fitted using equations 10-19 (See Methods) in Origin Pro using fixed parameters for protein, tracer concentration, and K_D = 64 nM for Flu-HIF-1α (determined beforehand, Fig. S2).

Compound	$K_{d,app}$ (nM) FA competition against p300–Flu-HIF-1α
**$CITED2_{218\text{-}256}$**	2.0 ± 0.9[a]
**1**	9.6 ± 2.4
**2**	4.9 ± 0.8
**3a**	> 600
**3b**	112 ± 13
**3c**	76 ± 6
**4**	1.7 ± 0.7

[a] Average and standard deviation of two individual titrations (Fig. 2 and Fig. S11)

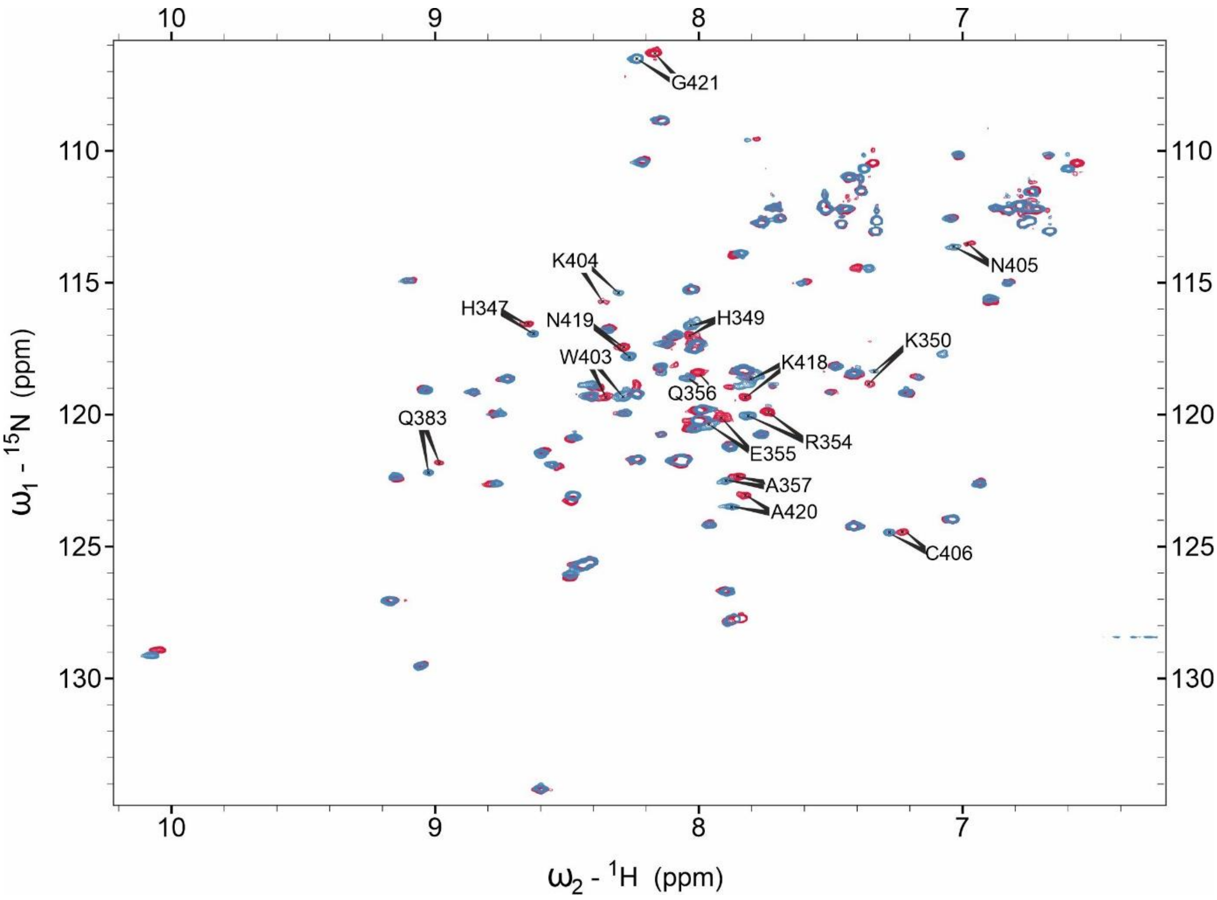

Fig. S12 **^{1}H-^{15}N HSQC spectra of CITED2$_{218\text{-}256}$ and 1 in complex with p300.**

Overlayed ^{1}H-^{15}N HSQC spectra of **CITED2$_{218\text{-}256}$** (red) and **1** (blue) in complex with p300. Spectra were recorded at 298K in 10mM Tris pH 6.9, 50 mM NaCl, 1 mM DTT, 0.02% NaN_3, and 10% D_2O. $\Delta\delta = [(\Delta\delta_H)^2 + (\Delta\delta_N/5)^2]^{1/2}$; $\Delta\delta_{average}$ = 0.021 ppm, σ = 0.029 ppm; p300 residues that shifted significantly ($\Delta\delta > 0.9\ \Delta\delta_{average} + \sigma$) are labelled.

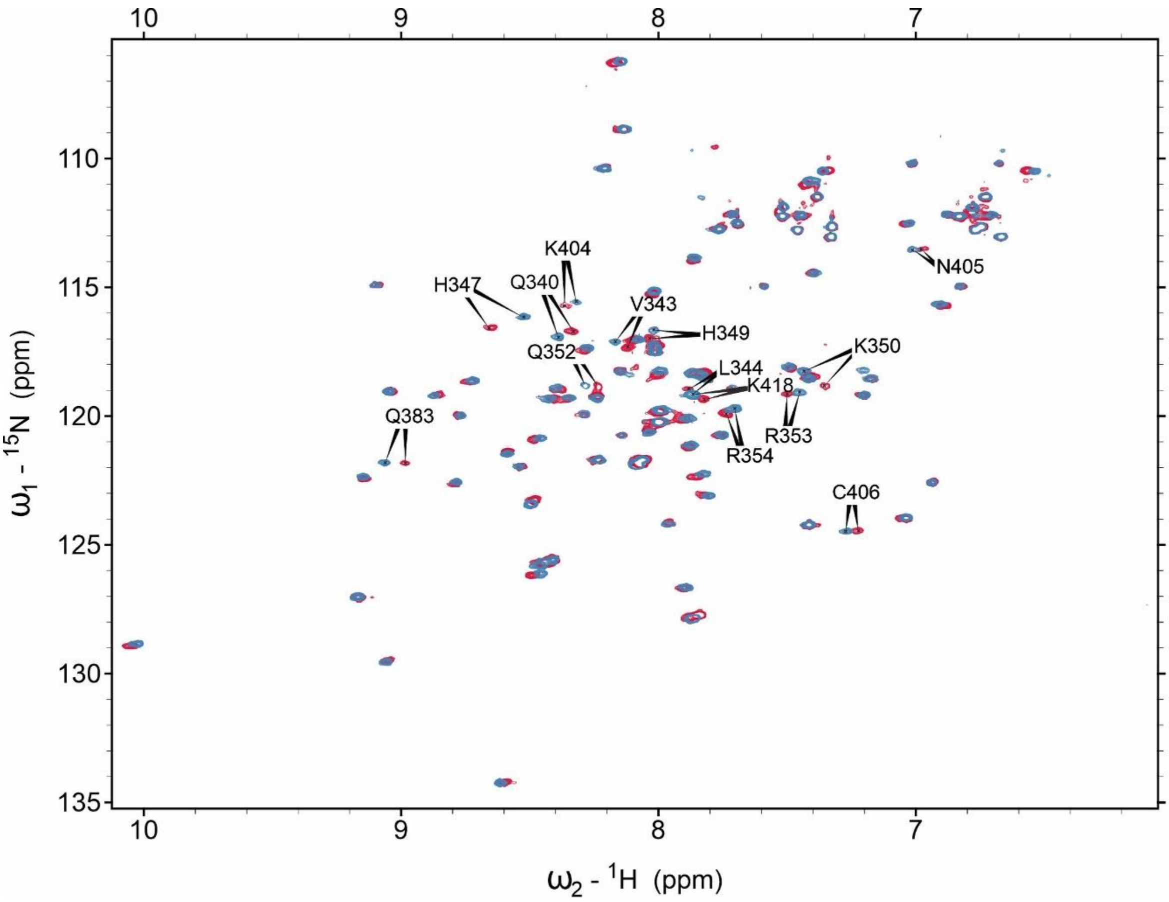

Fig. S13 **^{1}H-^{15}N HSQC spectra of CITED2$_{218\text{-}256}$ and 2 in complex with p300.**

Overlayed ^{1}H-^{15}N HSQC spectra of **CITED2$_{218\text{-}256}$** (red) and **2** (blue) in complex with p300. Spectra were recorded at 298K in 10mM Tris pH 6.9, 50 mM NaCl, 1 mM DTT, 0.02% NaN_3, 10% D2O. $\Delta\delta = [(\Delta\delta H)2 + (\Delta\delta_N/5)^2]^{1/2}$; $\Delta\delta_{average}$ = 0.021 ppm, σ = 0.026 ppm; p300 residues that shifted significantly ($\Delta\delta > 0.9\ \Delta\delta_{average} + \sigma$) are labelled.

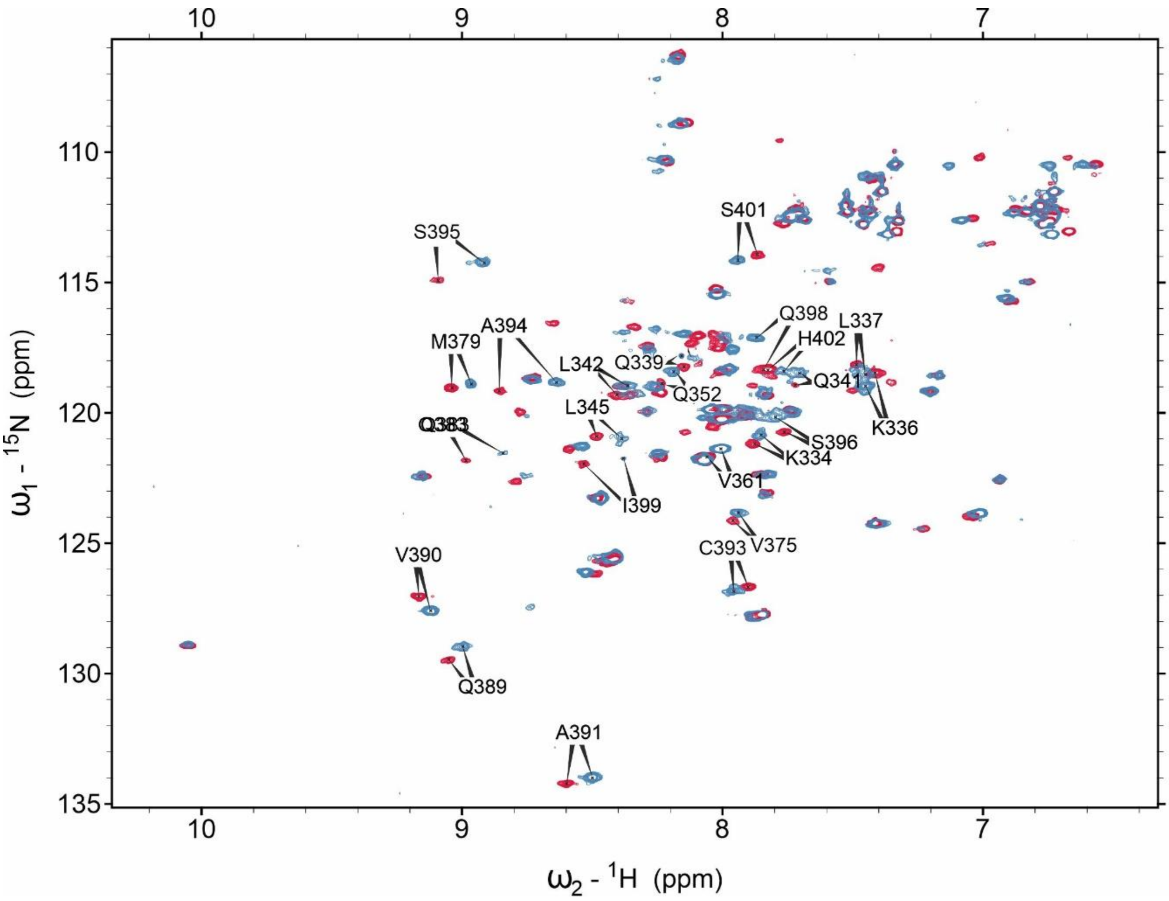

Fig. S14 **^{1}H-^{15}N HSQC spectra of CITED2$_{218\text{-}256}$ and 3a in complex with p300.**
Overlayed ^{1}H-^{15}N HSQC spectra of **CITED2$_{218\text{-}256}$** (red) and **3a** (blue) in complex with p300. Spectra were recorded at 298K in 10mM Tris pH 6.9, 50 mM NaCl, 1 mM DTT, 0.02% NaN_3, 10% D_2O. $\Delta\delta = [(\Delta\delta_H)^2 + (\Delta\delta_N/5)^2]^{1/2}$; $\Delta\delta_{average}$ = 0.041 ppm, σ = 0.052 ppm; p300 residues that shifted significantly ($\Delta\delta > 0.9\ \Delta\delta_{average} + \sigma$) are labelled.

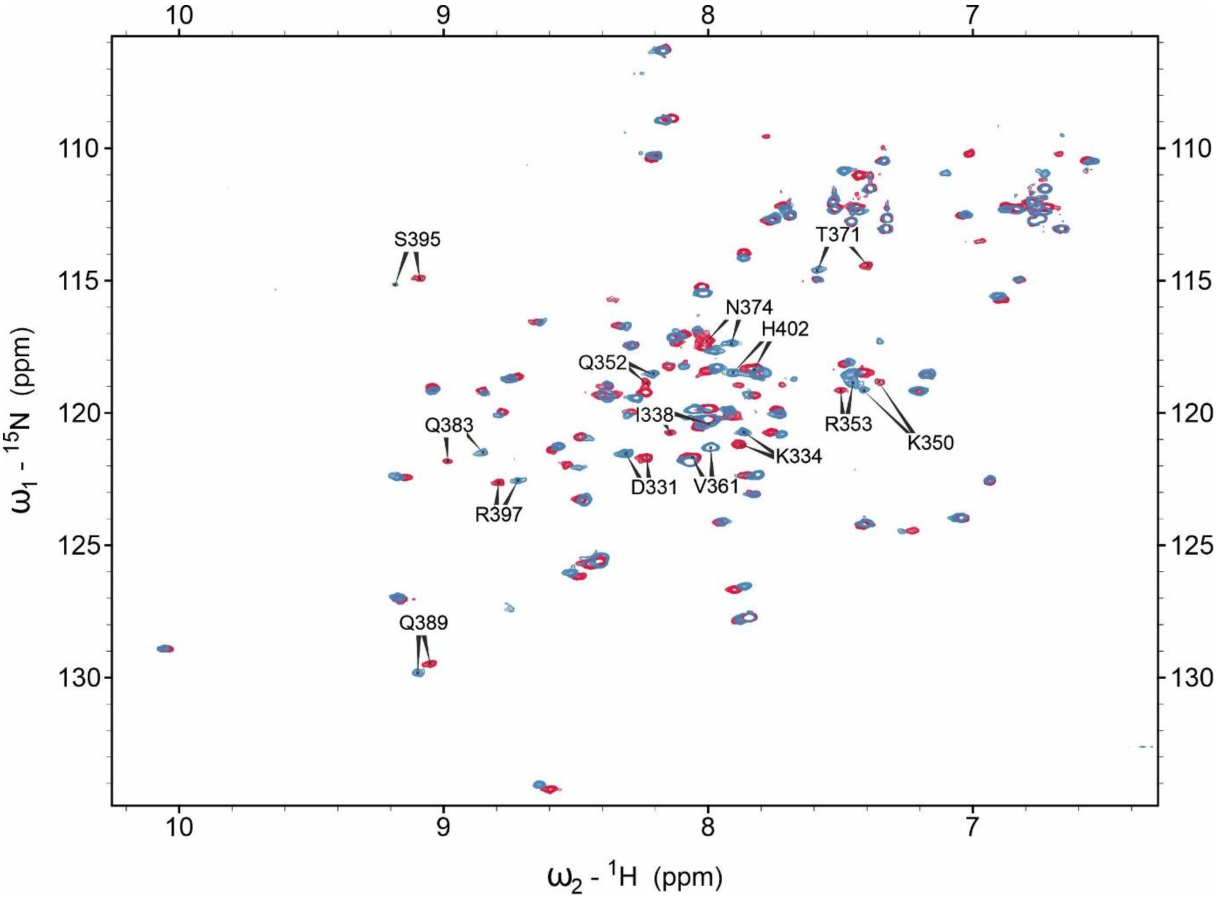

Fig. S15 **^{1}H-^{15}N HSQC spectra of CITED2$_{218\text{-}256}$ and 3b in complex with p300.**

Overlayed ^{1}H-^{15}N HSQC spectra of **CITED2$_{218\text{-}256}$** (red) and **3b** (blue) in complex with p300. Spectra were recorded at 298K in 10mM Tris pH 6.9, 50 mM NaCl, 1 mM DTT, 0.02% NaN_3, 10% D_2O. $\Delta\delta = [(\Delta\delta_H)^2 + (\Delta\delta_N/5)^2]^{1/2}$; $\Delta\delta_{average}$ = 0.034 ppm, σ = 0.037 ppm; p300 residues that shifted significantly ($\Delta\delta > 0.9\ \Delta\delta_{average} + \sigma$) are labelled.

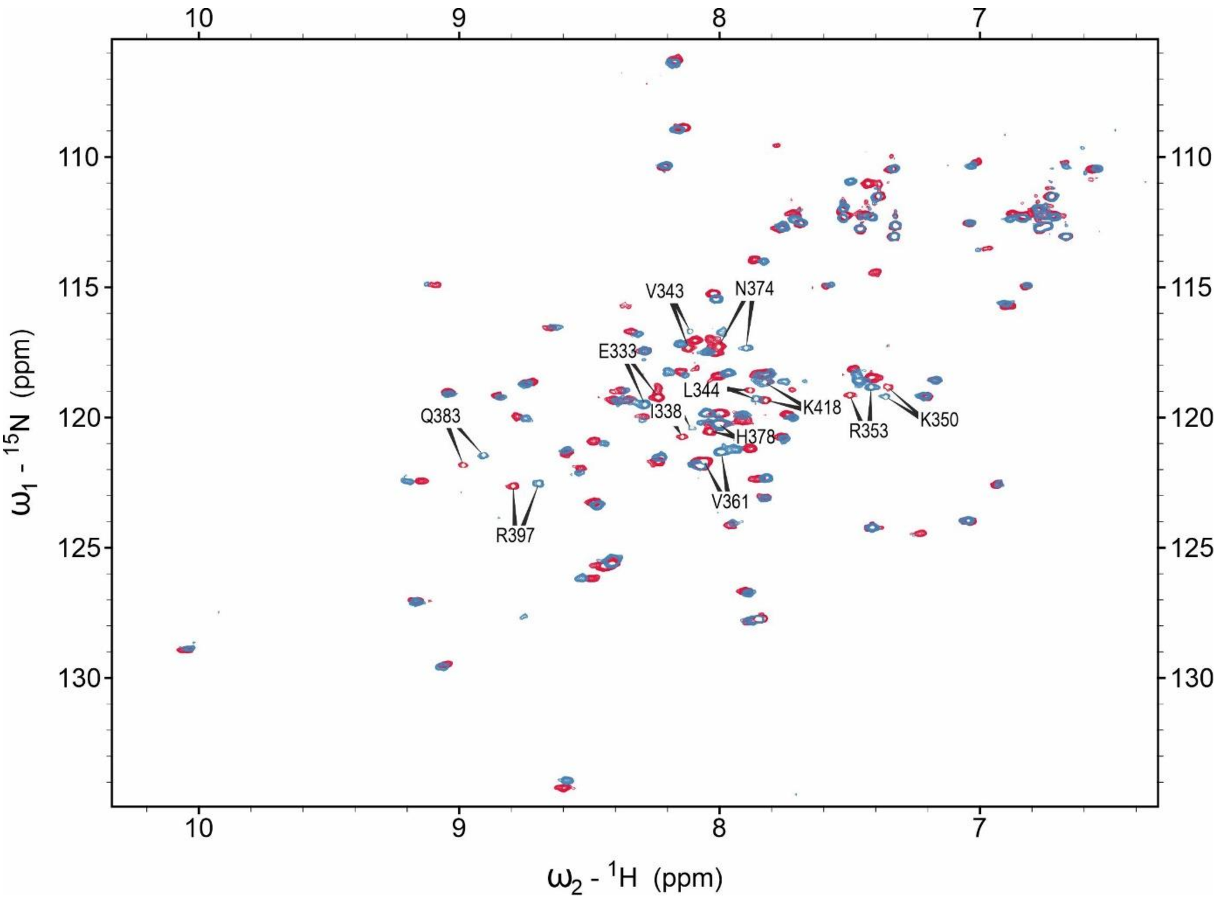

Fig. S16 **^1H-^{15}N HSQC spectra of CITED2$_{218\text{-}256}$ and 3c in complex with p300.**

Overlayed ^1H-^{15}N HSQC spectra of **CITED2$_{218\text{-}256}$** (red) and **3c** (blue) in complex with p300. Spectra were recorded at 298K in 10mM Tris pH 6.9, 50 mM NaCl, 1 mM DTT, 0.02% NaN_3, 10% D_2O. $\Delta\delta = [(\Delta\delta_H)^2 + (\Delta\delta_N/5)^2]^{1/2}$; $\Delta\delta_{average} = 0.031$ ppm, $\sigma = 0.032$ ppm; p300 residues that shifted significantly ($\Delta\delta > 0.9\ \Delta\delta_{average} + \sigma$) are labelled.

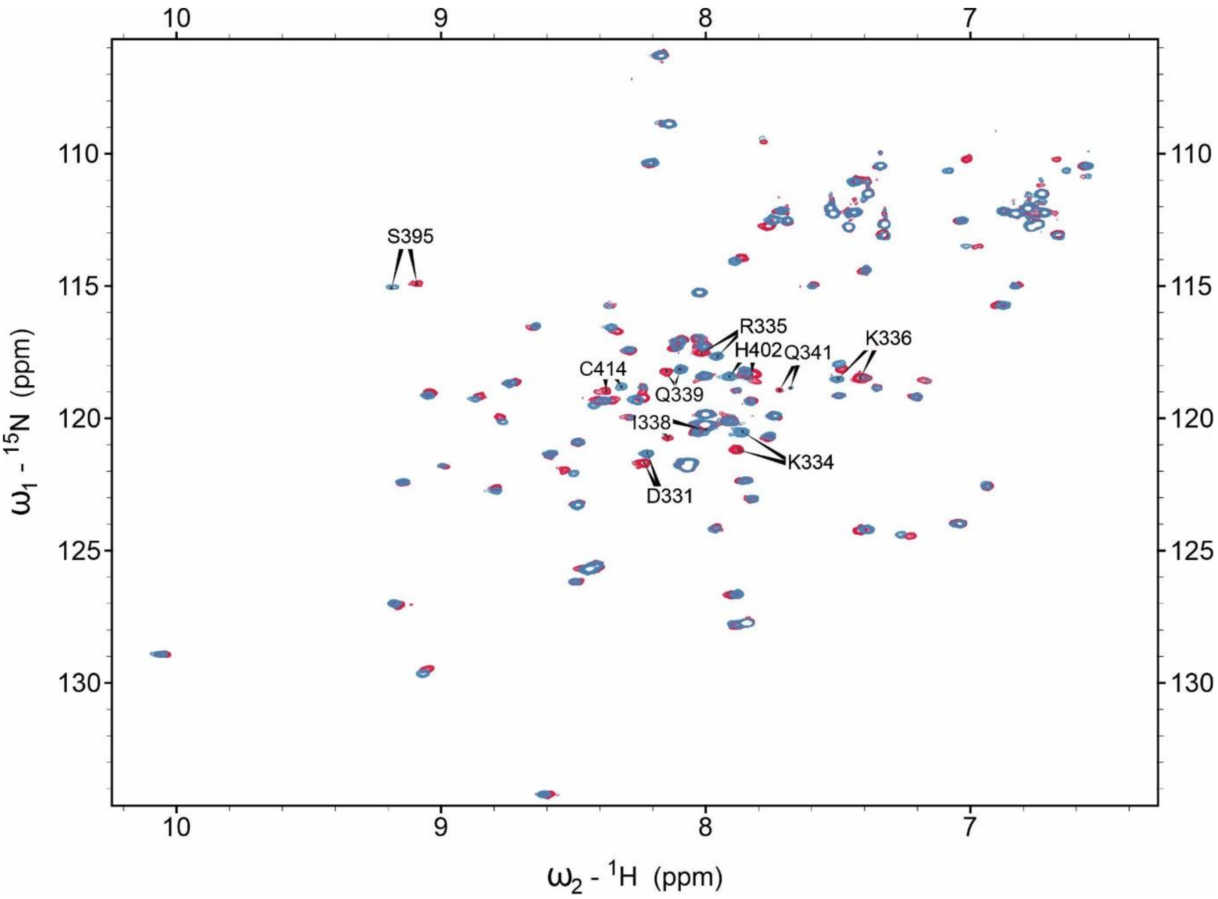

Fig. S17 **^{1}H-^{15}N HSQC spectra of CITED2$_{218-256}$ and 4 in complex with p300.**

Overlayed ^{1}H-^{15}N HSQC spectra of **CITED2$_{218-256}$** (red) and **4** (blue) in complex with p300. Spectra were recorded at 298K in 10mM Tris pH 6.9, 50 mM NaCl, 1 mM DTT, 0.02% NaN_3, 10% D_2O. $\Delta\delta = [(\Delta\delta_H)^2 + (\Delta\delta_N/5)^2]^{1/2}$; $\Delta\delta_{average}$ = 0.02 ppm, σ = 0.029 ppm; p300 residues that shifted significantly ($\Delta\delta > 0.9\ \Delta\delta_{average} + \sigma$) are labelled.

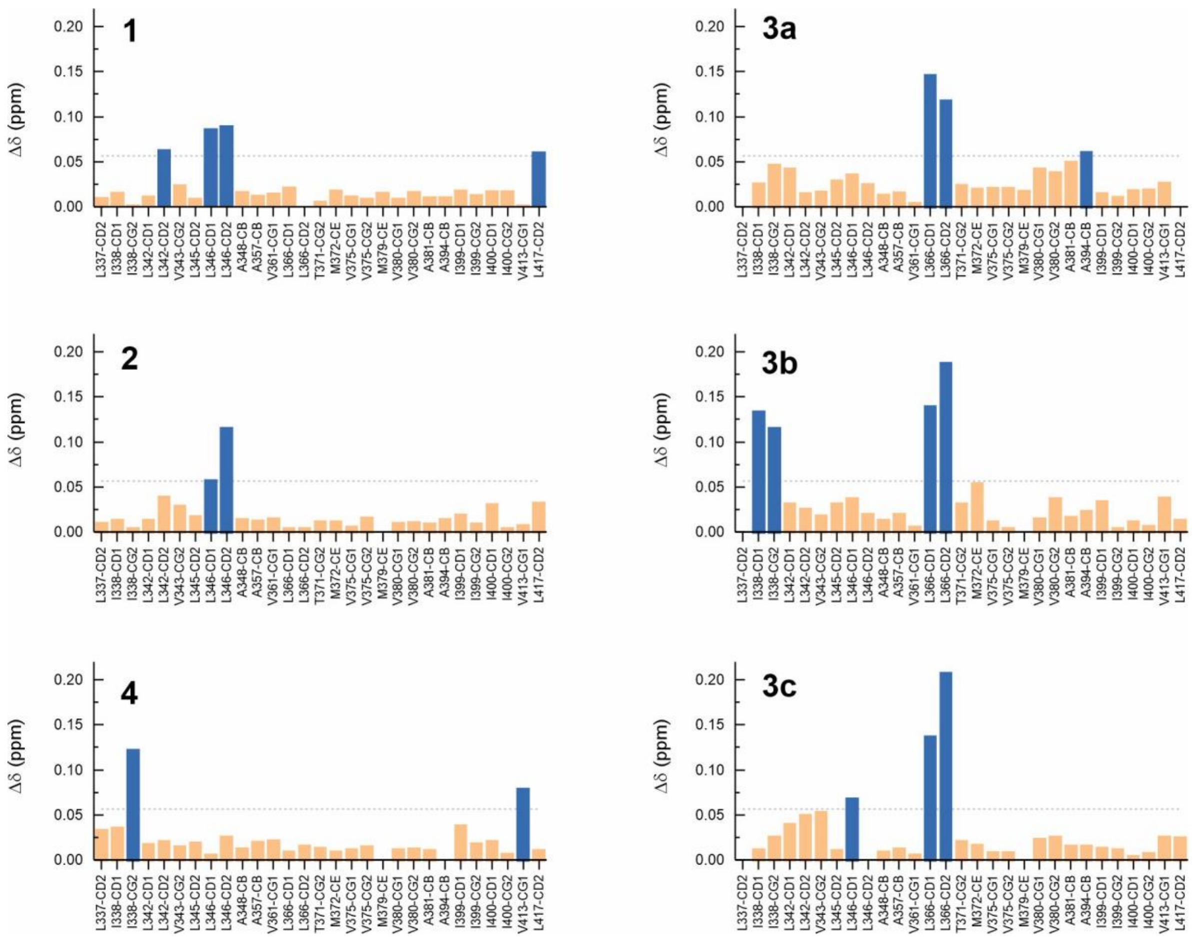

Fig. S18 Weighted average chemical shift differences of p300 CH_3 resonances

Weighted average chemical shift differences of the assigned CH_3 resonances for p300-bound **1-4** relative to the p300–CITED2$_{218\text{-}256}$ complex. $\Delta\delta = [(\Delta\delta_H)^2 + (\Delta\delta_C/5)^2]^{1/2}$ Resonances that shifted significantly ($\Delta\delta > 0.9\ \Delta\delta_{average} + \sigma$) highlighted blue.

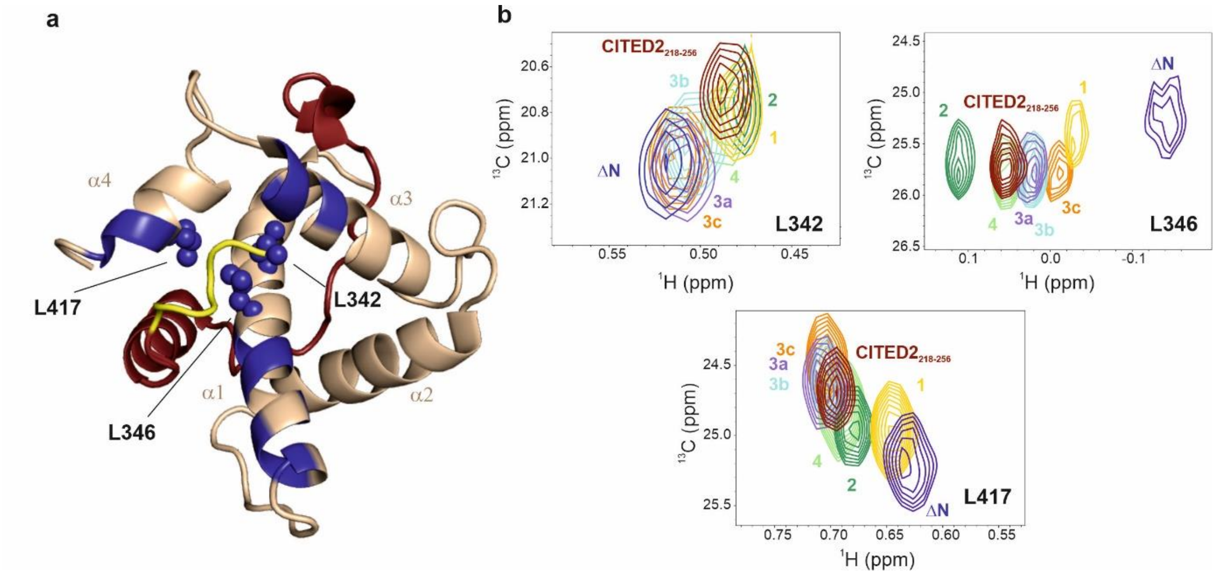

Fig. S19 **p300 CH_3 chemical shifts affected by CITED2 modifications**

a p300 (wheat) in complex with CITED2 (red) with leucine residues located at the α1/α4 interface. **b** Methyl resonance regions of the ^{1}H-^{13}C HSQC spectra of p300 for L342, L417 and L346 in complex with the different CITED2 variants; $CITED2_{224\text{-}256}$ is indicated by “ΔN”.

Methods

Protein expression and purification

All incubation steps were carried out in an Excella E24R (New Brunswick) incubator shaker at 160 rpm. Centrifugation steps were carried out at 4°C using an Avanti JXN-26 centrifuge equipped with JLA 8.1 or JLA 25.50 rotor. Sonication was performed using Qsonica Q500 Sonicator equipped with a ½" probe. Size exclusion chromatography was carried out on an ÄKTApurifier (GE Healthcare) system controlled by Unicorn software. Proteins were concentrated using Amicon Ultra-15 10K concentrators at 4°C using a Hettich 320R centrifuge equipped with a swing-out rotor. Antibiotic concentrations used for protein expression: chloramphenicol: 50 µg/ml carbenicillin: 100 µg/ml.

The DNA sequence of human p300 (UniProtKB - Q09472) containing the TAZ1 domain (Zinc finger, CH1, TAZ type 1) from residues 330-420 (used for FA and ITC experiments) or 330-424 (used for NMR experiments, both referred to as p300) was cloned into pGEX-4T-1 plasmid using BamHI/XhoI restriction sites. Expression and purification of GST-p300 were performed as described previously [7]. Briefly, BL21 E.Coli pLysS transformed with pGEX-4T1-p300 were grown at 37 °C to OD600 0.6-0.8 and induced with 0.1 mM IPTG and 50 µM $ZnSO_4$ was added and incubated overnight at 18 °C. Cells were harvested, lysed by sonication, and centrifuged at 25.000 g for 15 minutes at 4 °C. The supernatant was applied to glutathione beads (Glutathione Sepharose 4B, GE Healthcare) and washed with 20 mM Tris, pH 8, 150 mM NaCl, 1 mM DTT, and 20 mM Tris, pH 8, 500 mM NaCl, 1 mM DTT. GST was cleaved overnight at 4 °C using Thrombin protease. The eluted fractions were concentrated and purified by size-exclusion chromatography on S75 26/60 pg column in 40 mM Na-phosphate, 100 mM NaCl, 1 mM DTT, pH 7.4 buffer (for ITC and FA measurements) or in 10 mM Tris, 50 mM NaCl, 1 mM DTT pH 6.9 (for NMR measurements). Pure protein was analyzed by SDS-PAGE and mass spectrometry (Fig. S19). Concentration was determined by UV-VIS spectroscopy using Nanodrop One (Thermo Scientific) using 5500 M^{-1} cm^{-1} extinction coefficient, determined by Expasy ProtParam [8].

For the expression of ^{15}N,^{13}C-labeled $p300_{330\text{-}424}$ in minimal (M9) media, a starter culture was grown from BL21 E.Coli pLysS at 37 °C in 5 mL LB supplemented with antibiotics for 6 hours. 100 µL of this LB growth was used to inoculate 12 mL M9 media (without isotopically labeled substances) supplemented with antibiotics and grown at 37°C overnight. The 12 ml M9 growth was used to inoculate 500 mL M9 supplemented with ^{15}N-NH_4Cl and ^{13}C-glucose (Cambridge Isotope Laboratories, Inc.) and grown at 37°C until OD600 = 0.6. Induction was performed using 0.2 mM IPTG and 50 µM $ZnSO_4$ at 18°C overnight. Bacteria were harvested and protein purified using the same method described above.

Peptide synthesis and purification

Peptides were synthesized at 0.1 mmol scale on Tentagel R RAM resin (resin loading 0.19 mmol/g, Iris Biotech) using CEM Liberty Blue synthesizer. Couplings were performed using 5 equivalent amino acid excesses for α-amino acids, and 3 equivalent excesses for β-amino acids. Coupling reagents were DIC

(Diisopropylcarbodiimide, Fluorochem) and Oxyma (Ethyl cyano(hydroxyimino)acetate, Fluorochem) dissolved in DMF (VWR). The deprotection solution contained 10% (w/v) piperazine (Molar Chemicals) dissolved in 10% absolute ethanol/NMP mixture. Required amounts were calculated using the built-in reagent calculator of Liberty Blue. All amino acids were coupled using high swelling (HS) Liberty Blue methods with cycle settings as described before, using double couplings for α-amino acids[9]. For β-amino acids, the following modified cycle was used: Step 1: Deprotection: 3mL, 75°C Initial Deprotection (Bubble 2s on, 3s off; 75°C 40W 30s 2°C ΔT); Step 2: Deprotection: 3mL, 75°C Deprotection (Bubble 2s on, 3s off; 75 °C 40W 180s 2°C ΔT); Step 3-6: Wash: 4mL, Step 7: Coupling: Amino acid 2.5 mL, Activator 1 mL, Activator base 0.5 mL, manifold wash volume 2 mL, Microwave method: 75°C Coupling (Bubbles 2s on, 3s off; 75°C 30W 600s 2°C ΔT); Step 8-9: Wash: 4 mL

Acetylation

Peptides were acetylated using 10 equivalents of acetic anhydride and DIPEA in DCM:DMF 1:1 in a fritted SPE tube for 20 minutes 2 times at room temperature.

Fluorescent labelling

Fmoc-aminohexane carboxylic acid (Ahx) was coupled manually to the N terminus of the peptide using 5 equivalent amino acid excess, HATU as the activator, and DIPEA as the base for 3 hours at room temperature. Fmoc deprotection was carried out using 2% DBU, 2% piperazine solution in DMF for 5 and then 15 minutes. The resin was washed with DMF and DCM. 5(6)-carboxyfluorescein (Flu) was coupled using 3 equivalent excess, HATU, and DIPEA as coupling reagents overnight at room temperature. The resin was washed with the deprotection solution before cleavage to remove non-specific carboxyfluorescein esters.

Cleavage

Peptides were cleaved using TFA:DTT:TIS:H_2O mixture (90:2.5:2.5:5) for 3 hours, after which TFA was evaporated, and the crude peptide was precipitated in ice-cold diethyl-ether. The precipitate was washed with ether, then redissolved in ACN:H_2O mixture and lyophilized.

Peptide purification

Preparative RP-HPLC was carried out on a JASCO PU-4180 system equipped with a diode array detector (MD-4015) and an automatic fraction collector (Advantec, CHF122SC). Fraction collection was monitored and programmed using ChromNAV software. Peptides were dissolved in DMSO and purified on a Phenomenex Luna C18 (250 x 10 mm or 250 x 20 mm) column using appropriate gradients. The purity of the peptides was characterized by HPLC-UV and HPLC-MS measurements using Dionex Ultimate 3000 HPLC system equipped with a diode array detector. It interfaced with an LTQ XL (Thermo Scientific) ion trap mass spectrometer. For analytical measurements Aeris Widepore C18 (5 mm, 100 Å, 250 x 4.6

mm) column and the following gradient: 0 min 5% B, 25 min 100% B, 1 ml/min flow rate or 25-75% B on Kinetex EVO C18 (250 x 4.6 mm). The following eluents were used for HPLC-UV methods: A: 0.1% TFA/H_2O; B: 0.1% TFA/ACN and for HPLC-MS: A: 0.1% HCOOH/H_2O; B: 0.1% HCOOH/ACN.

Circular dichroism

Measurements were carried out on a JASCO J-1100 CD spectrometer at room temperature using a 1mm cylindrical quartz cuvette. Samples were prepared in 10 mM Na-phosphate, 1 mM DTT, pH 7.4 buffer at 20 μM peptide concentration. Spectra were recorded from 180 – 250 nm at a scan speed of 100 nm min^{-1} using 5 accumulations. CD spectra were smoothed, and the buffer background was subtracted and normalized to mean residue ellipticity (MRE).

Isothermal titration calorimetry

Measurements

Before ITC measurement, p300 TAZ1 was run on a S75 column in the buffer used for titration (40 mM Na-phosphate, pH 7.5 100 mM NaCl, 1 mM DTT), and the same buffer was used to dissolve the pure peptides. Experiments were carried out using a Microcal VP ITC instrument. Direct titrations to p300 were carried out using 5 μM protein in the cell and 60 μM ligand in the syringe at 35°C, using 5 μl injections and 180 s spacing between injections, for 40-50 injections. For competition ITC measurements, the p300/HIF-1$\alpha_{776-826}$ complexes were prepared by titrating HIF-1$\alpha_{776-826}$ to p300 in the cell using 5 μM protein concentration, and the titration stopped when it reached close to saturation conditions (i.e., no significant difference in injection heats). This resulted in 1.2-1.5 excess of ligand compared to p300 in the cell. The additional volume was removed from the cell, and the complex was titrated with CITED2 or **1-4** using 60 μM concentration in the syringe, 5 -10 uL injections with 180-240 s spacing.

Data analysis

Raw data integration was performed using SVD analysis implemented in NITPIC[10] and fitted using SEDPHAT[11] to a competition model that includes cooperative interactions and ternary complex formation (Figure S20).[11,12]

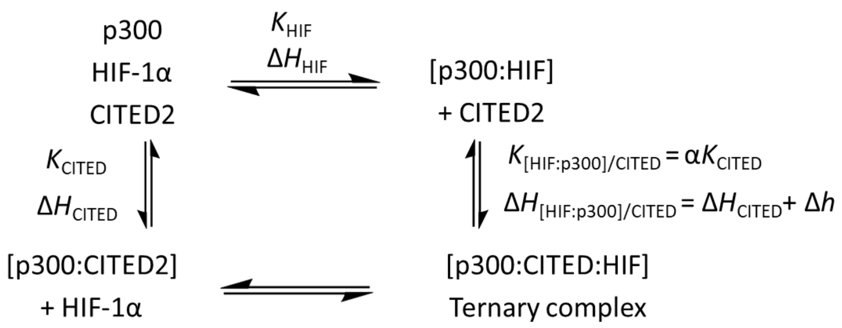

Fig. S20 Parametrized diagram of the model used to fit cooperative parameters for the CITED-HIF-1α-p300 system.

Fitted parameters were K_{CITED}, ΔH_{CITED}, K_{HIF}, ΔH_{HIF}, Δh and the cooperativity constant α ($K_{[p300:HIF]/CITED}/K_{CITED}$) which are defined as follows:

$$[\text{p300:HIF}] = K_{\text{HIF}}\,[\text{p300}][\text{HIF}]\ (1)$$

$$[\text{p300:CITED}] = K_{\text{CITED}}\,[\text{p300}][\text{CITED}]\ (2)$$

$$[\text{p300:HIF:CITED}] = K_{[\text{HIF:p300}]/\text{CITED}}\,[\text{p300:HIF}][\text{CITED}]\ (3)$$

$$[\text{p300:HIF:CITED}] = K_{[\text{CITED:p300}]/\text{HIF}}[\text{p300:CITED}][\text{HIF}]\ (4)$$

Cooperativity constant α for CITED2 binding to the preformed p300-HIF complex is defined by:

$$K_{[\text{HIF:p300}]/\text{CITED}} = \alpha K_{\text{CITED}}\ (5)$$

$$\alpha = \frac{K_{[HIF:p300]/CITED}}{K_{CITED}}\ (6)$$

From which the cooperative enhancement of binding CITED2 to the preformed p300-HIF complex was calculated as follows:

$$\Delta G_{[p300:HIF:CITED]} = -RTln(\alpha K_{CITED}\,K_{HIF}) = \Delta G_{HIF} + \Delta G_{CITED} + \Delta g\ (7)$$

$$\Delta H_{[p300:HIF:CITED]} = \Delta H_{HIF} + \Delta H_{CITED} + \Delta h\ (8)$$

$$-T\Delta s = \Delta g - \Delta h\ (9)$$

Where Δ*g* (and Δ*h*) is the additional contribution to binding free energy (and enthalpy) due to cooperative interactions when a ternary system is formed. In SEDPHAT the fitting model corresponds to the "*A+B+C <-> AB + C <-> AC + B <-> ABC triple complex*". Two titrations were fitted simultaneously: CITED2 (or variant) to p300 (parametrized as C to A) and CITED2 (or variant) to p300-HIF-1α complex (parametrized as C to AB) the concentration of the HIF-1α competitor was given as the molar ratio of HIF-1α:p300 (B/A). Restraints were applied for K_{HIF} and ΔH_{HIF} (B to A titration) based on average values of previous direct titrations (Fig. S2). Fitting included an incompetent fraction fit for p300 and baseline correction. Detailed descriptions of the fitting model and equations can be found in references 11,13,14. 68 % confidence intervals for the fitted values were determined using the automatic confidence interval search

implemented in SEDPHAT (propagated from integration error estimates). Figures were prepared using GUSSI.[15]

Fluorescence anisotropy

Fluorescence anisotropy was measured using a Clariostar Plus microplate reader in a 384-well black plate with excitation at 480 nM and emission at 535 nm at 35°C. The buffer used for the experiments was 40 mM Na-phosphate, 100 mM NaCl, 1 mM DTT, 0.01% Triton-X, pH 7.4. Three repeats were performed for each titration, and in parallel, a control experiment was prepared where the fluorescently labeled Flu-HIF-1$\alpha_{786\text{-}826}$ was replaced with buffer in the reaction mixture, which was subtracted from the raw data. The competitor peptides (HIF-1$\alpha_{776\text{-}826}$, CITED2, and **1-4**) were diluted by mixing 40 µL peptide solution with 20 µL buffer in the first well and then serially diluted over 24 points on the plate (2/3 dilution series) resulting in 5 µM as the highest concentration in the first well. To each well p300 at a final concentration of 50 nM and tracer (Flu-HIF-1$\alpha_{786\text{-}826}$) at a final concentration of 25 nM was added. Plates were incubated for 10 minutes at 35°C before reading.

Fitting model and data analysis

Fluorescence intensity and anisotropy were calculated according to the following equations as previously described.[9]

$$I = (2PG)+S \quad (10)$$

$$r = \ (S–PG) \quad (11)$$

r = anisotropy, I = total intensity, P = perpendicular intensity, S = parallel intensity, G = an instrument factor set to 1.

Apparent competition K_D ($K_{D,app}$) values for the competitor peptides were fitted using the method described in references 16,17 using the following equations in Origin Pro.

$$r = \frac{(\gamma r_{P:HIF} \ + \ r_{HIF})}{1 + \ \gamma} \quad (12)$$

r = fluorescence anisotropy; *HIF* = Flu-Ahx-HIF-1$\alpha_{786\text{-}826}$, *P* = $p300_{330\text{-}420}$. $r_{P:HIF}$ is the anisotropy of the p300-bound Flu-HIF-1α, r_{HIF} is the anisotropy of the free Flu-HIF-1α, where:

$$\gamma = \frac{[P{:}HIF]}{[HIF]_t - [P{:}HIF]} \quad (13)$$

[P:HIF] = concentration of the p300-bound Flu-HIF-1α; $[HIF]_t$ = total concentration of Flu-HIF-1α. *[P:HIF]* is given by:

$$[P:HIF] = \frac{\left([HIF]_t \left\{2\sqrt{a^2-3b}\cos\left(\frac{\theta}{3}\right)-a\right\}\right)}{3K_{HIF}+\left\{2\sqrt{a^2-3b}\cos\left(\frac{\theta}{3}\right)-a\right\}} \quad (14)$$

where:

$$\theta = \arccos\frac{-2a^3+9ab-27c}{2\sqrt{(a^2-3b)^3}} \quad (15)$$

$$a = K_{HIF} + K_{CIT} + [HIF]_t + [CIT]_t - [P]_t \quad (16)$$

$$b = K_{HIF}K_{CIT} + K_{HIF}([CIT]_t - [P]_t) + K_{CIT}([HIF]_t - [P]_t) \quad (17)$$

$$c = -[P]_t K_{HIF} K_{CIT} \quad (18)$$

K_{HIF} = dissociation constant for Flu-HIF-1α, K_{CIT} = dissociation constant for the competitor CITED2 or variant, $[CIT]_t$ = total concentration of the competitor CITED2 or variant, $[P]_t$ = total concentration of protein.

Fitted parameters were: K_{HIF}, K_{CIT}, $[HIF]_t$, $[P]_t$, $r_{P:HIF}$, r_{HIF} from which K_{HIF}, $r_{P:HIF}$, r_{HIF}, $[HIF]_t$ were kept constant during fitting. K_{HIF}, $r_{P:HIF}$, r_{HIF} was determined in a separate experiment using HIF-1α$_{776-826}$ as a competitor (Fig. S3) $[HIF]_t$ was determined using fluorescence intensity measurements relative to a fluorescein calibration curve. $[P]_t$ was kept as a floating parameter to account for incompetent protein fraction. Anisotropy values were normalized and represented as ligand-bound (L_b) values (Eq. 19.)

$$L_b = \frac{(r - r_{HIF})}{(r_{P:HIF} - r_{HIF})} * [HIF] \quad (19)$$

NMR measurements

Samples were prepared in 10 mM Tris pH 6.9, 1 mM DTT, 50 mM NaCl, 10% D_2O, 0.02% NaN_3 buffer. ^{15}N-^{13}C-p300$_{330-424}$ (100 μM) was mixed with 1.2-1.5 equivalent CITED2 peptide (added by volume from a 100 μM stock). The complex was concentrated using 10K Amicon Ultra filters (Merck) to its final volume resulting in 90-100 μM protein concentration. To prepare samples containing equimolar amounts of both HIF-1α and CITED2 variants, first ^{15}N-^{13}C-p300$_{330-424}$ was mixed with HIF-1α$_{776-826}$ (1.2 eq) then divided into three samples: to the first CITED2$_{224-256}$ was added, to the second CITED2$_{218-256}$ was added in 1.2 equivalent relative to p300 concentration, and to the third, same volume of buffer was added, and concentrated down to final sample volume. Concentrations were determined based on UV absorbance at 280 nm for p300 and CITED2 variants and at 205 nm for HIF-1α.

NMR experiments were carried out on a Bruker Avance III 600 MHz spectrometer equipped with a 5 mm CP-TCI triple-resonance cryoprobe. Measurements were carried out at 298 K. For p300–CITED2$_{218-256}$ and p300–CITED2$_{224-256}$ complex, the following spectra were recorded: ^{1}H-^{15}N-HSQC, ^{1}H-^{13}C-HSQC,

HNCO, HNCA, HN(CO)CA, CBCA(CO)NH and HCCH-TOCSY using pulse schemes implemented in Topspin 3.5 (Bruker), with excitation sculpting water suppression. Processing was carried out using Topsin 3.5, and data were analyzed using NMRFAM-Sparky[18]. Only ^{1}H-^{15}N-HSQC, ^{1}H-^{13}C-HSQC and HNCO were recorded for compounds **1-4**. Backbone and methyl resonance assignments were based on the previously published p300 TAZ1/CITED2 complex (BMRB 5788) and p300 TAZ1/HIF-1α complex (BMRB 5306).

Characterization data

a GSA$_{330}$DPEKRKLIQQQLVLLLHAHKCQRREQANGEVRQCNLPHCRTMKNVLNHMTHCQSGKSCQVAHCASSRQIISHWKNCTRHDCPVCLPLKNA$_{420}$

GSA$_{330}$DPEKRKLIQQQLVLLLHAHKCQRREQANGEVRQCNLPHCRTMKNVLNHMTHCQSGKSCQVAHCASSRQIISHWKNCTRHDCPVCLPLKNAGDKR$_{424}$

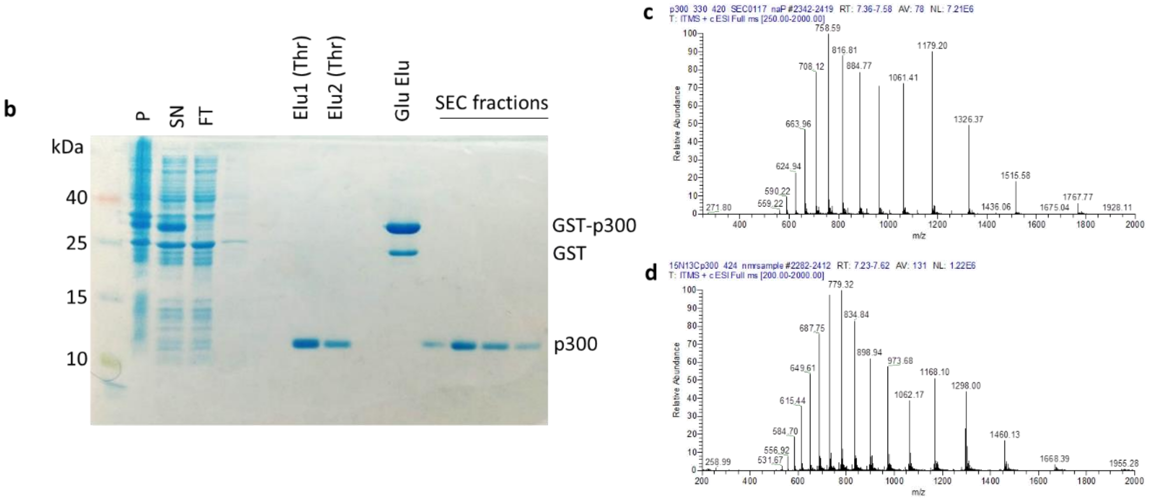

Fig. S21 p300 protein expression

a Sequences of the p300 TAZ1 domains used. **b** Representative SDS acrylamide gel of protein purification (P = pellet, SN = supernatant after sonication, FT = flowthrough, Elu1-2 (Thr) = elution fractions after on-column digestion with Thrombin, Glu Elu = elution with 20 mM glutathione, and size-exclusion chromatography (SEC) fractions of pure protein) **c** ESI Mass spectrum of pure p300$_{330-420}$ **d** ESI Mass spectrum of ^{15}N, ^{13}C-labeled p300$_{330-424}$. expected m/z for p300$_{300-420}$: 10603.30, measured m/z: 10603.41 and for ^{15}N-^{13}C-p300$_{300-424}$: 11681.8 (in case of 100% ^{13}C ^{15}N labeling), measured m/z: 11672.4.

HPLC chromatograms and mass spectra of the synthesized peptides

For all peptides an LC-UV (or LC-MS) and MS spectra is provided, including selective ion zoom scans for the highest intensity ions.

Ac-HIF-1α$_{776-826}$

Ac-SDLACRLLGQSMDESGLPQLTSYDCEVNAPIQGSRNLLQGEELLRALDQVN-NH$_2$

EM: 5599.72 MW: 5603.24. Measured: [M+3H]$^{3+}$: 1868.68; [M+4H]$^{4+}$: 1401.86; [M+5H]$^{5+}$: 1121.62;

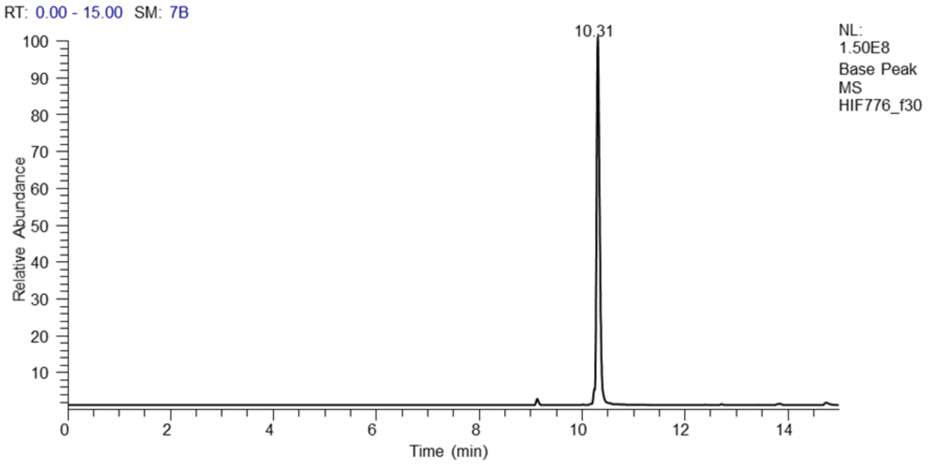

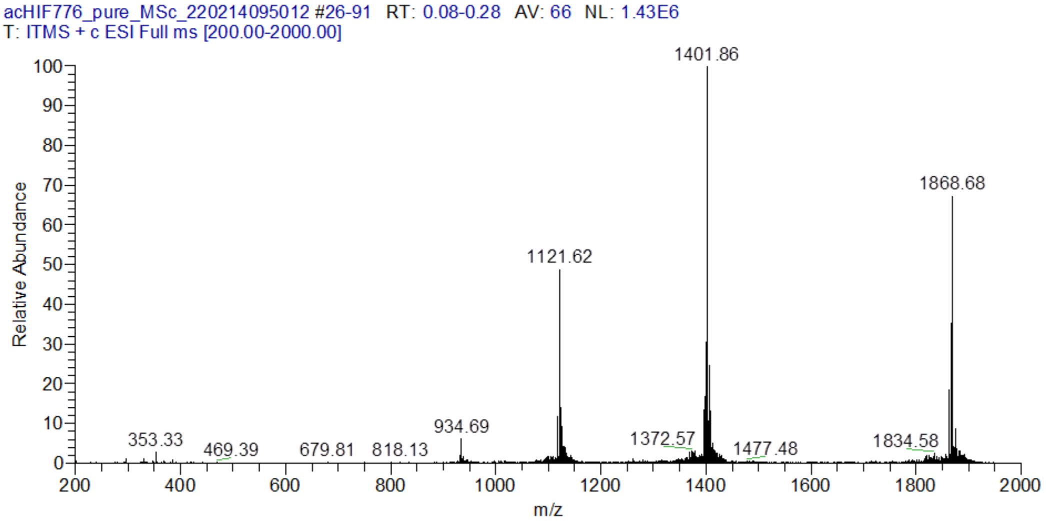

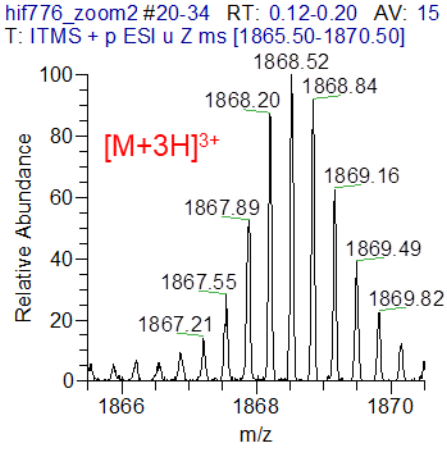

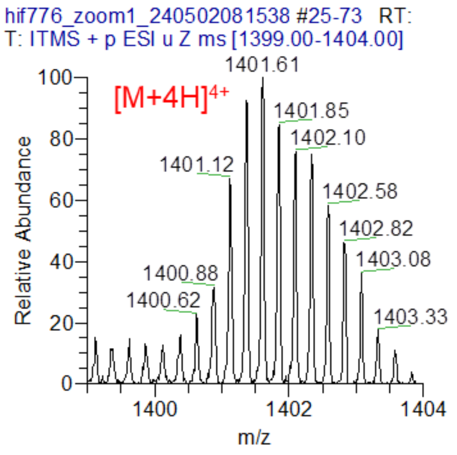

Flu-Ahx-HIF$_{786\text{-}826}$

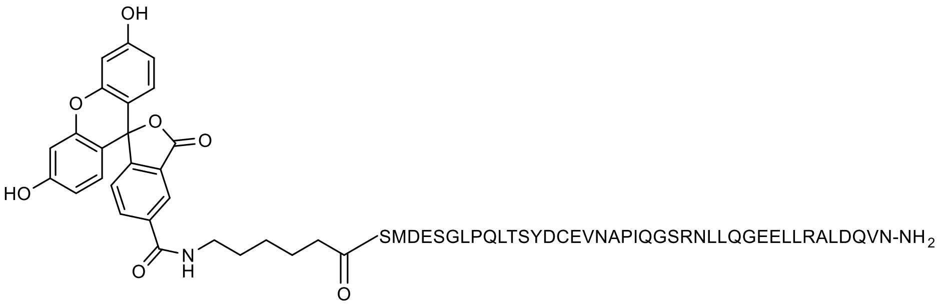

Chemical Formula: $C_{27}H_{22}NO_7^{\bullet}$
Exact Mass: 472.14
Molecular Weight: 472.47

EM: 4972.32, MW: 4975.43

Measured: $[M+3H]^{3+}$: 1659.25; $[M+4H]^{4+}$: 1244.67; $[M+5H]^{5+}$: 996.08

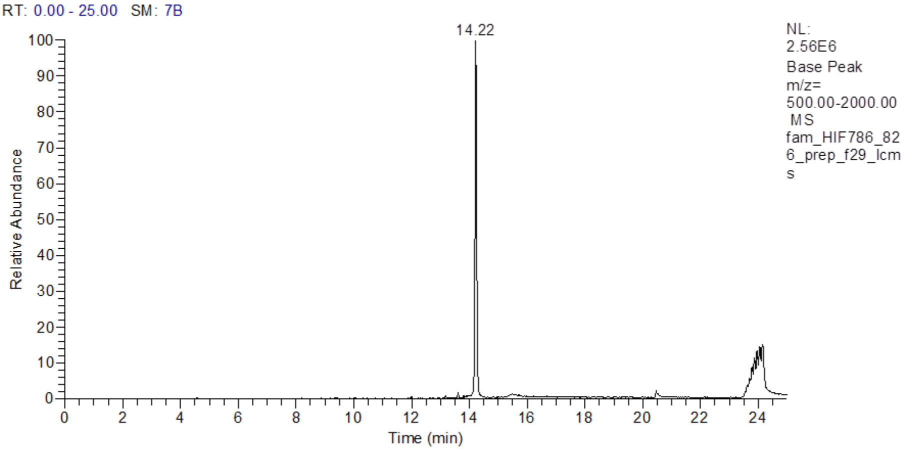

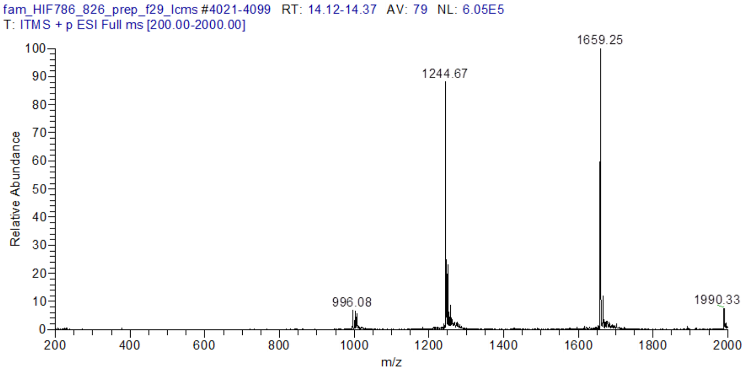

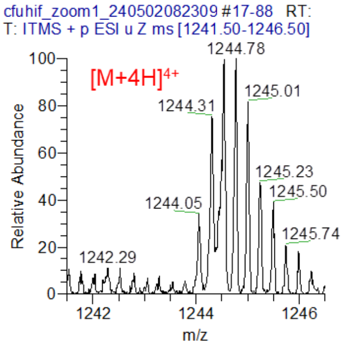

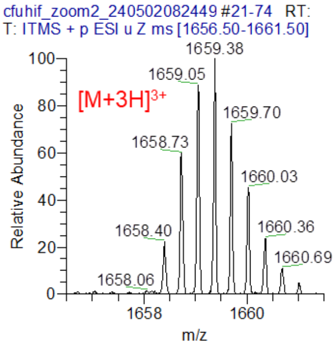

CITED2$_{224\text{-}256}$ (CITEDΔN)

Ac-DEEVLMSLVIEMGLDRIKELPELWLGQNEFDFM-NH_2

EM: 3978.935; MW: 3981.593, measured: $[M+2H]^{2+}$: 1991.62; $[M+3H]^{3+}$: 1328.12; $[M+4H]^{4+}$: 996.36

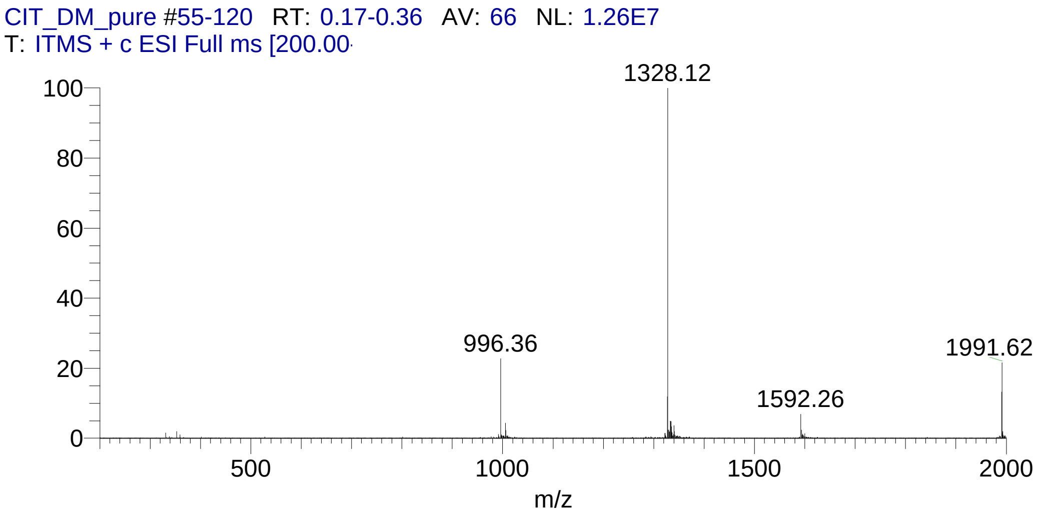

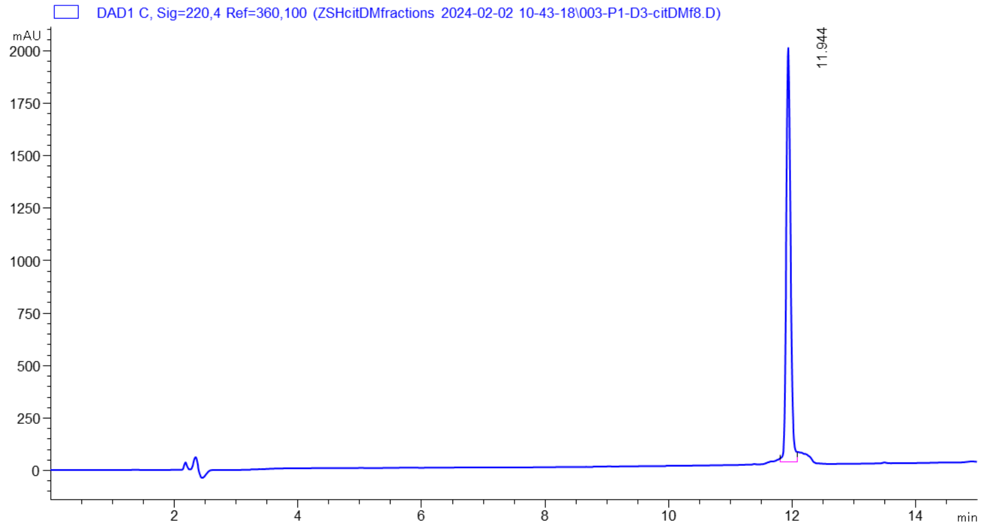

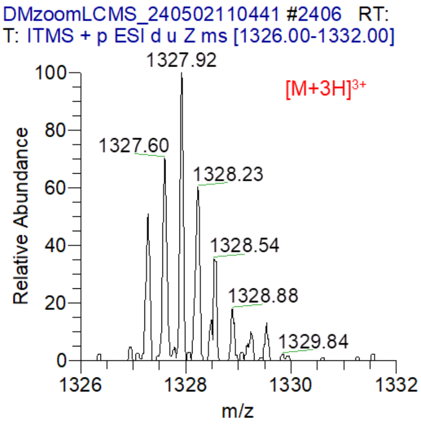

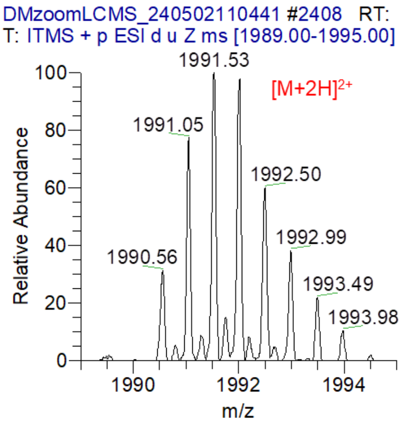

CITED2$_{224\text{-}259}$

Ac-DEEVLMSLVIEMGLDRIKELPELWLGQNEFDFMTDF-NH_2

EM: 4342.08; MW: 4344.96

$[2M+5H]^{5+}$: 1738.50; $[M+3H]^{3+}$: 1449.56; $[M+4H]^{4+}$: 1087.24

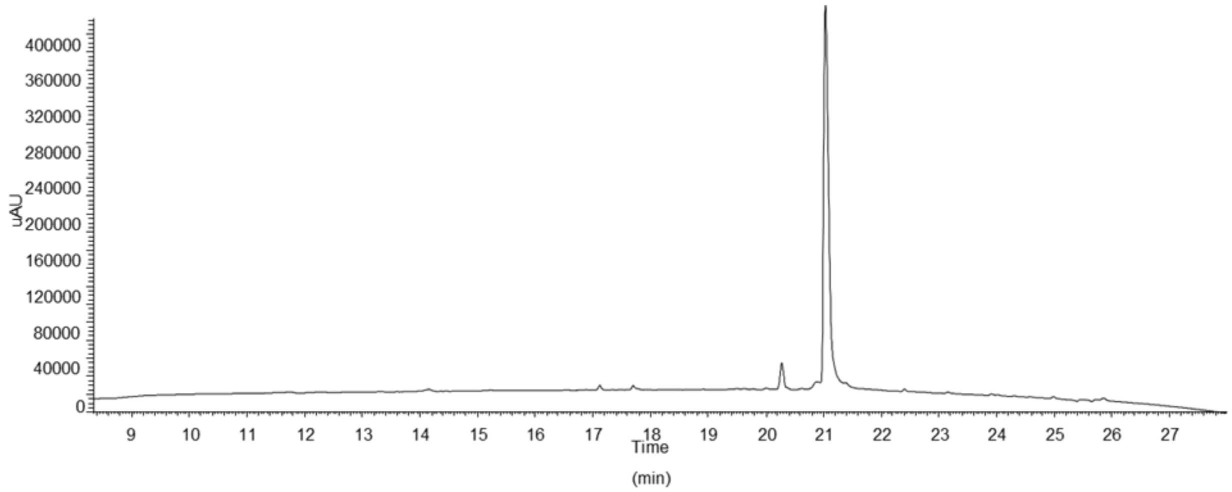

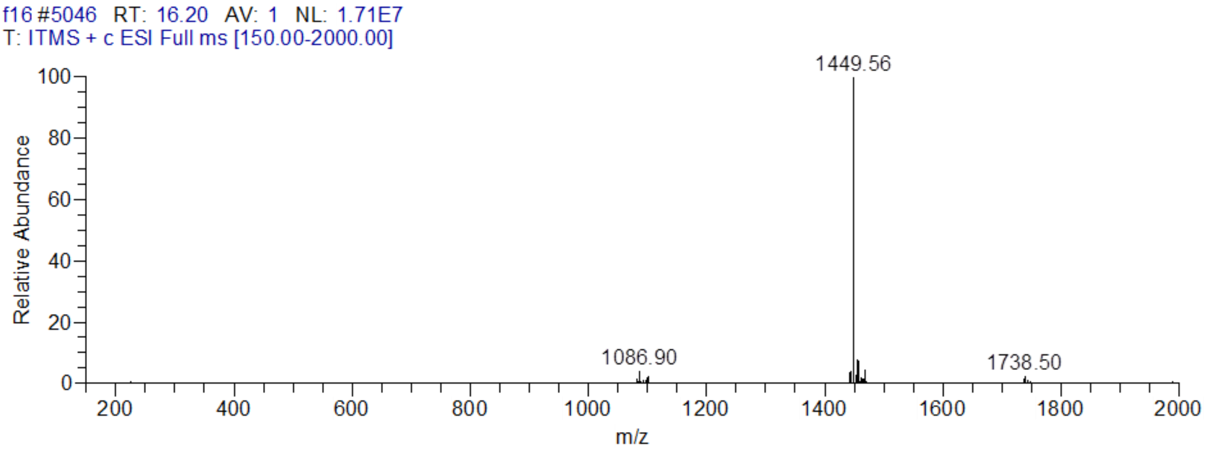

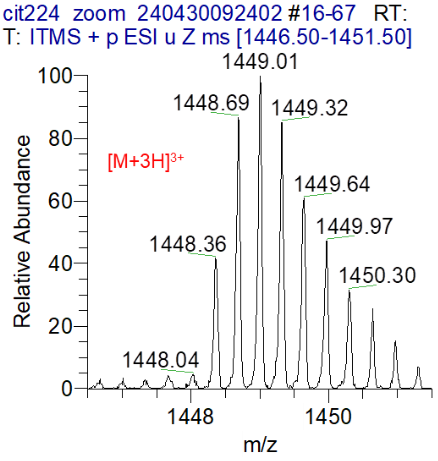

CITED2218-256

Ac-IDTDFIDEEVLMSLVIEMGLDRIKELPELWLGQNEFDFM-NH_2

EM: 4683.27; MW: 4686.37; measured: $[2M+5H]^{5+}$: 1874.83; $[M+3H]^{3+}$: 1563.17, $[M+4H]^{4+}$: 1172.58

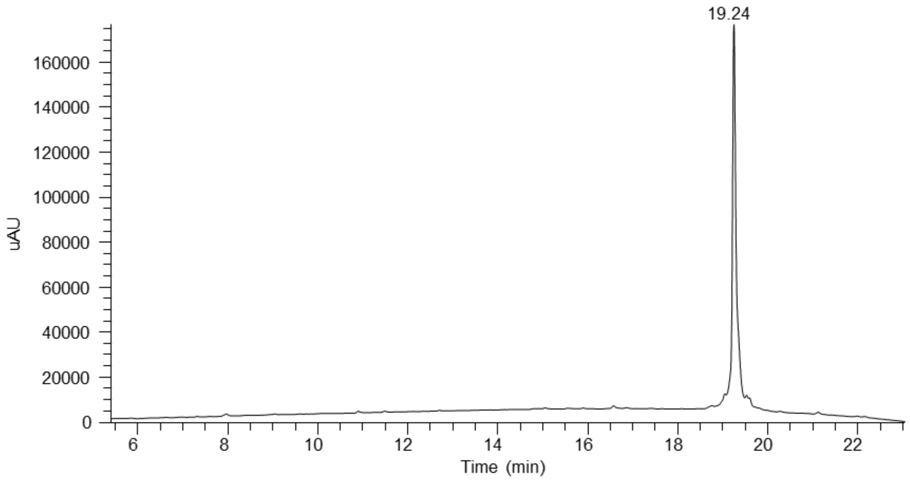

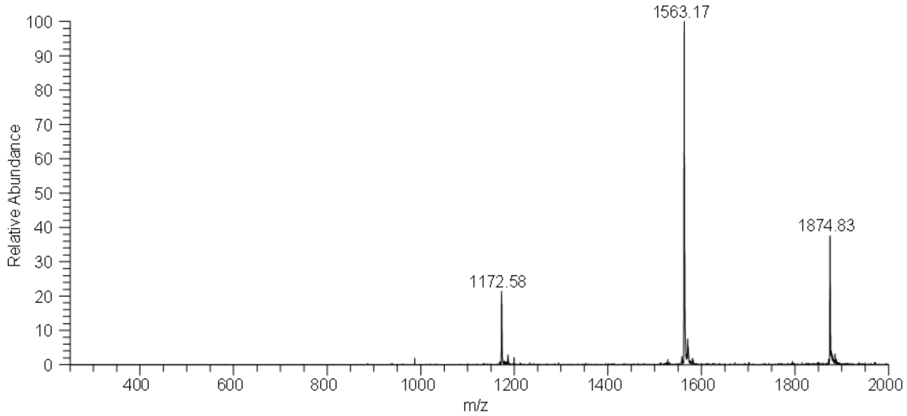

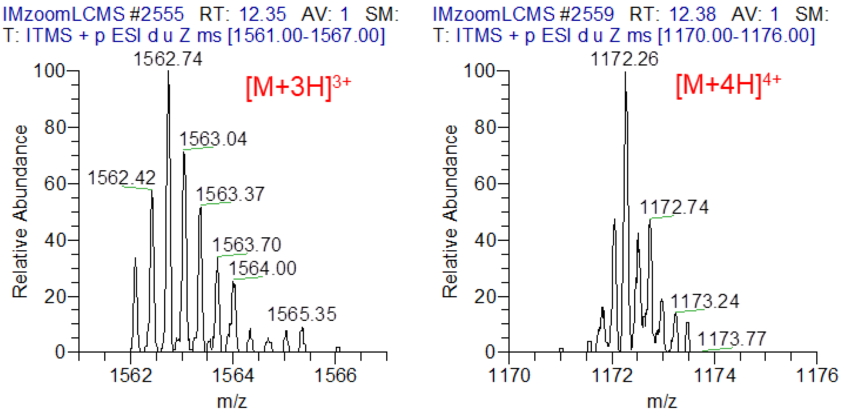

1

Ac-I**β^3D**T**β^3D**FI**β^3D**EEVLMSLVIEMGLDRIKELPELWLGQNEFDFM-NH_2

EM: 4725.27; MW: 4728.45, Measured: $[2M+5H]^{5+}$: 1891.94; $[M+3H]^{3+}$: 1577.30; $[M+4H]^{4+}$: 1183.33

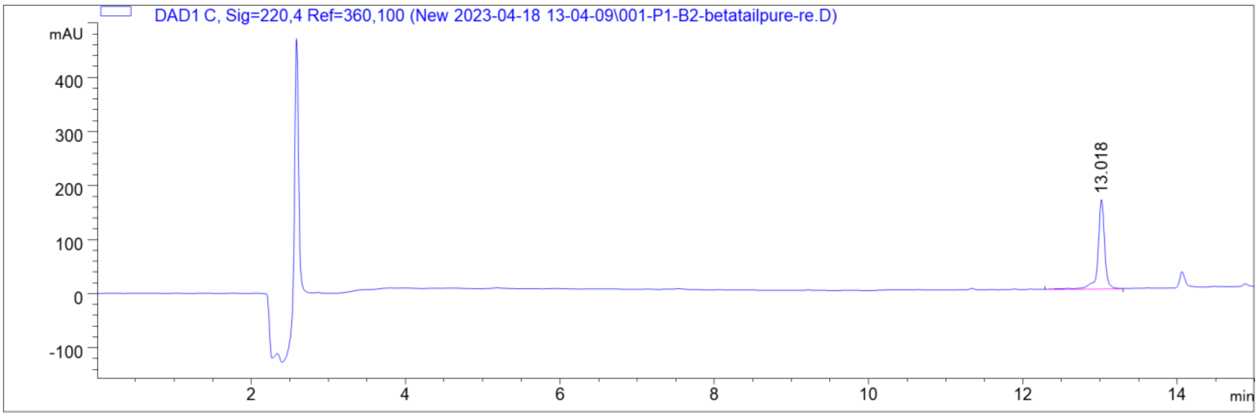

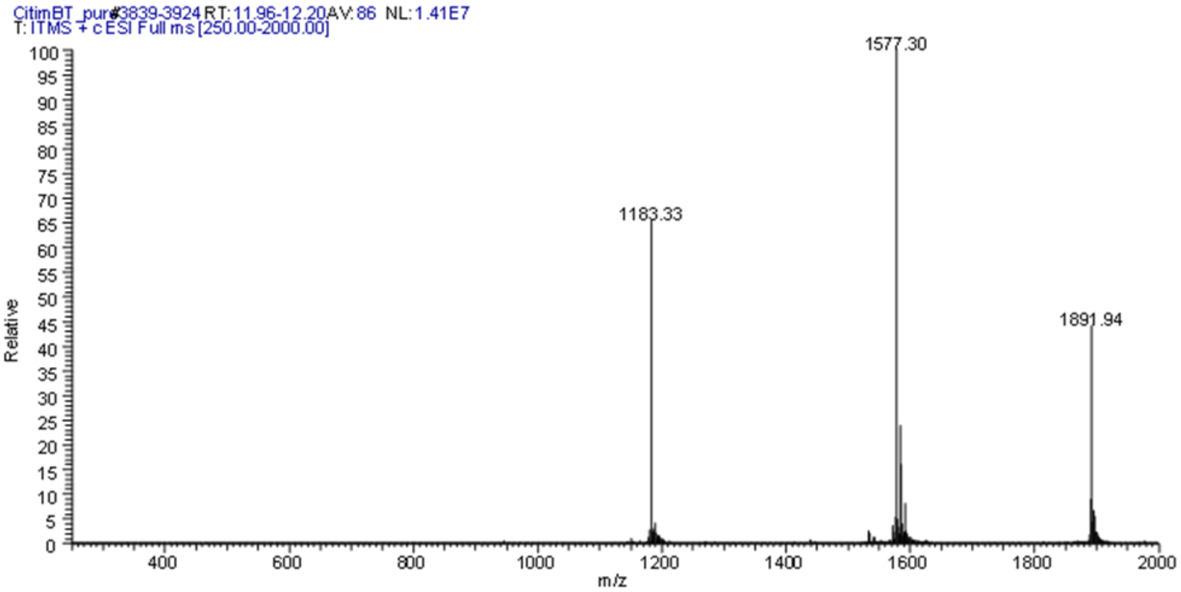

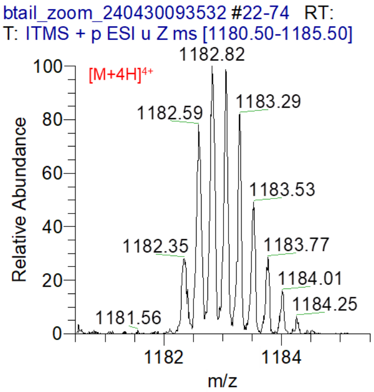

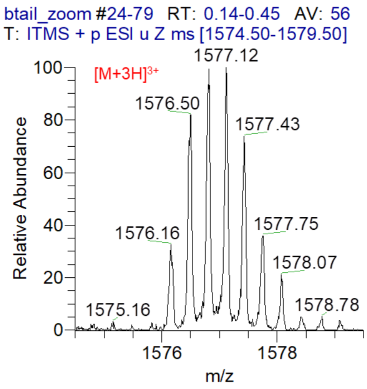

2

Ac-IDTDFIDE**β^3E**VLM**β^3S**LVI**β^3E**MGLDRIKELPELWLGQNEFDFM-NH_2

EM: 4725.27; MW: 4728.45

$[2M+5H]^{5+}$: 1892.10; $[M+3H]^{3+}$: 1577.69; $[M+4H]^{4+}$: 1184.09

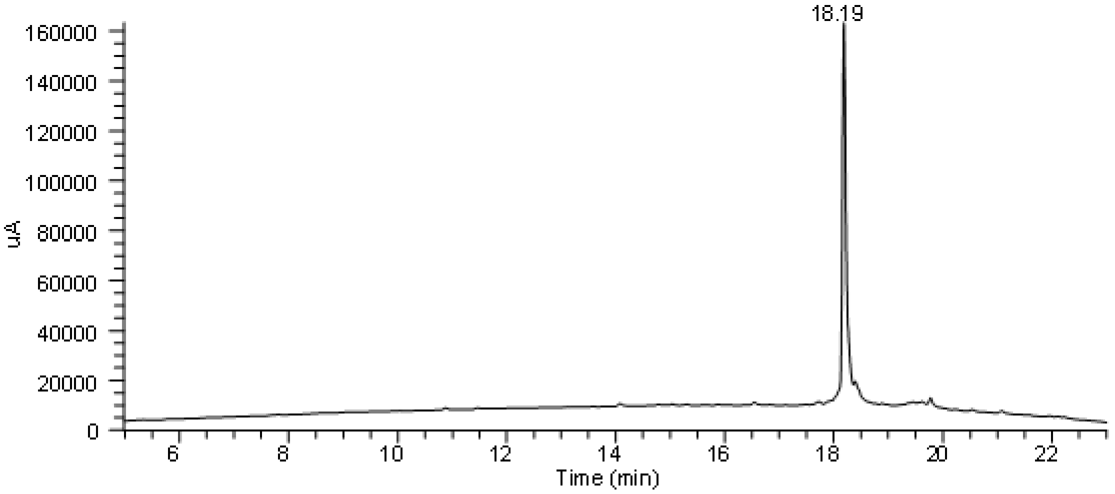

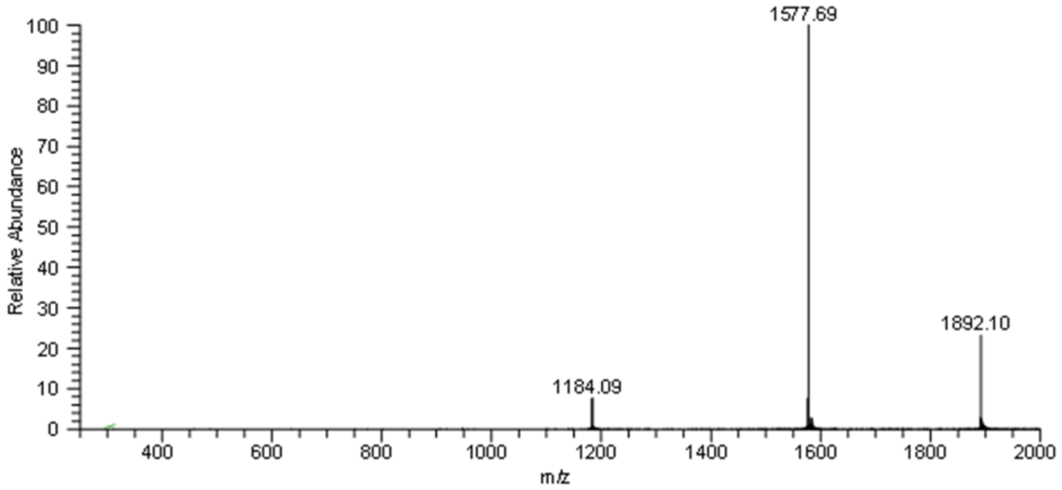

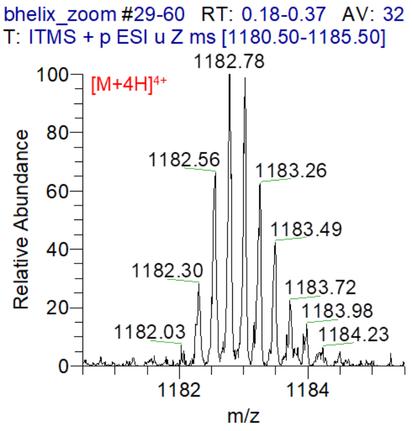

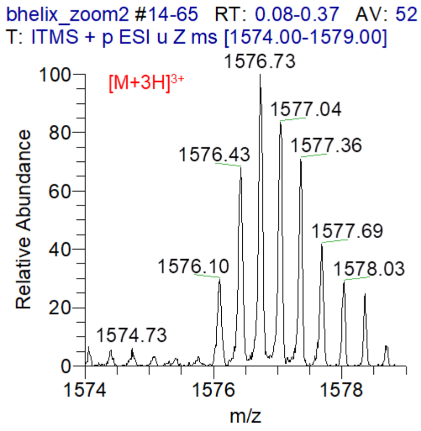

3a

Ac-IDTDFIDEEVLMSLVIEMGL**β[3]D**RIK**β[3]E**LP**β[3]E**LWLGQNEFDFM-NH_2

EM: 4725.27, MW: 4728.45

Measured: $[M+3H]^{3+}$: 1577.14; $[M+4H]^{4+}$: 1183.20

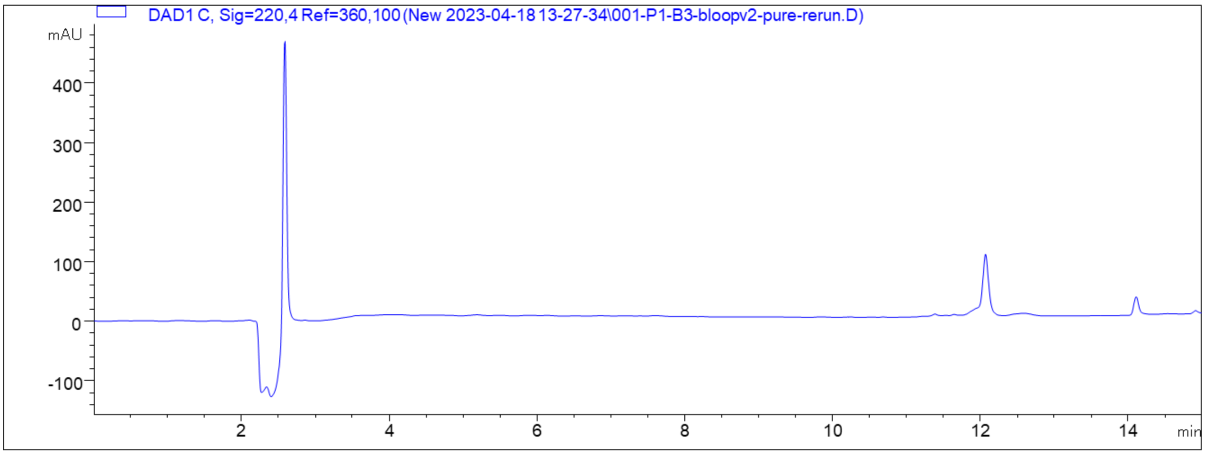

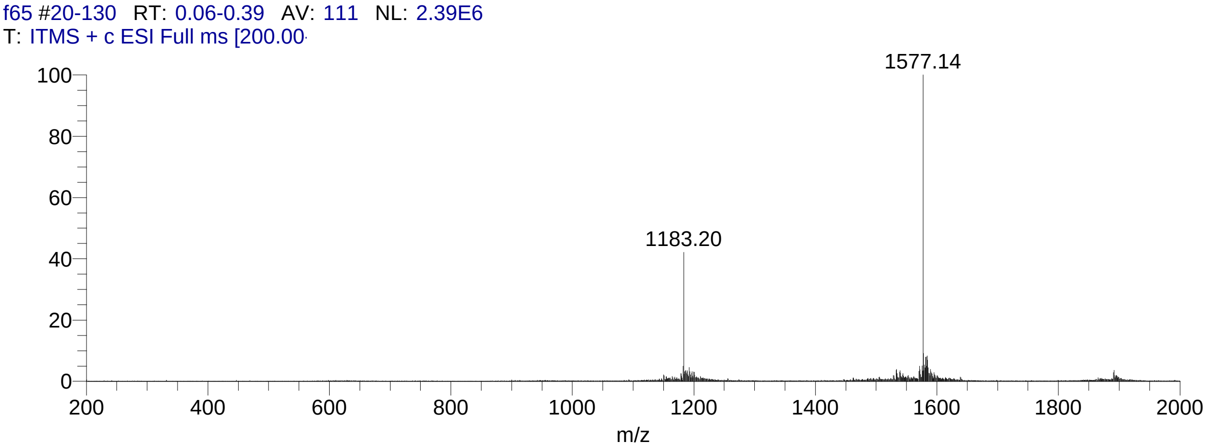

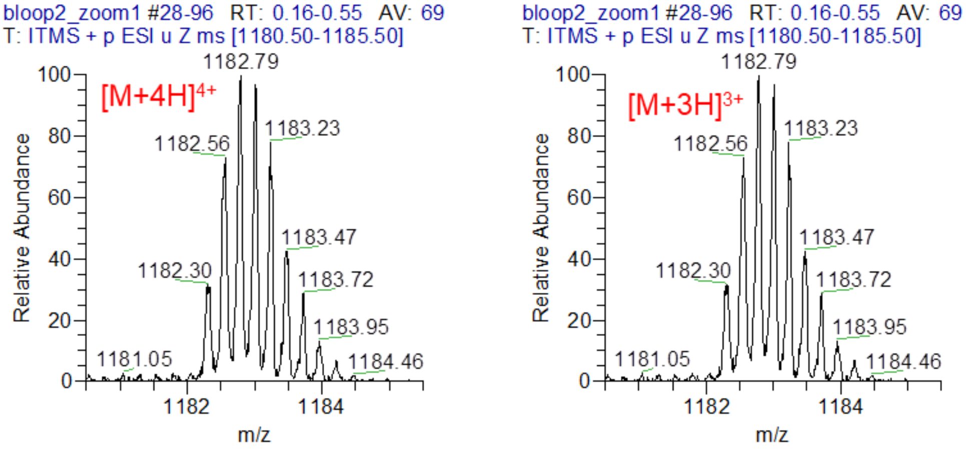

3b

Ac-IDTDFIDEEVLMSLVIEMGL**β^3D**RI**β^3E**LPELW**β^3L**GQNEFDFM-NH_2

EM: 4595.23, MW: 4600.28

Measured: $[M+3H]^{3+}$: 1534.87; $[M+4H]^{4+}$: 1151.49

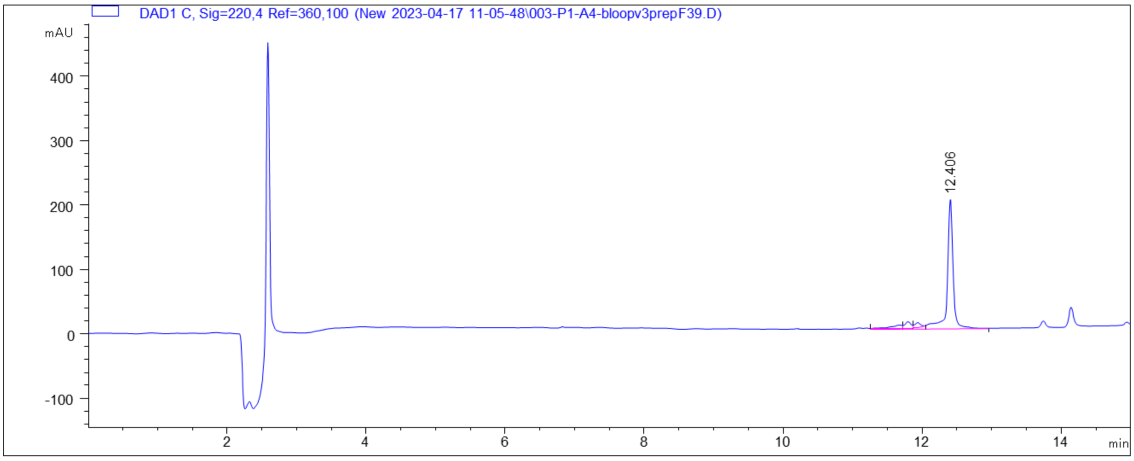

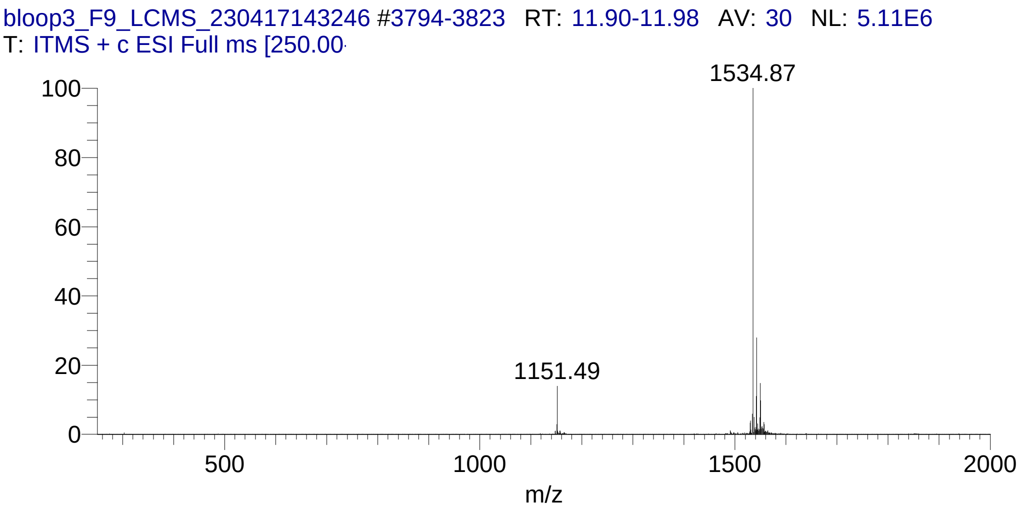

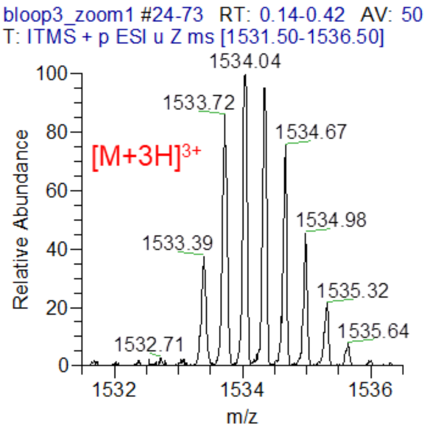

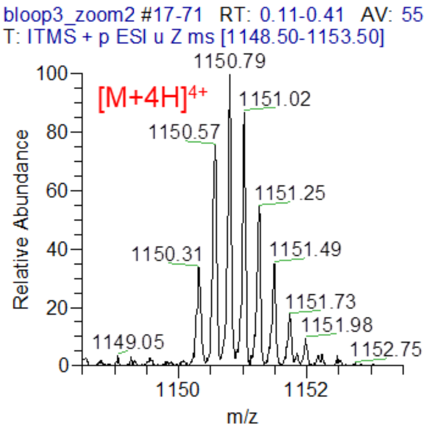

3c

Ac-IDTDFIDEEVLMSLVIEMGL**β^3D**I**β^3E**LPELWLGQNEFDFM-NH_2

EM: 4427.08; MW: 4430.06, Measured: $[M+3H]^{3+}$: 1477.54

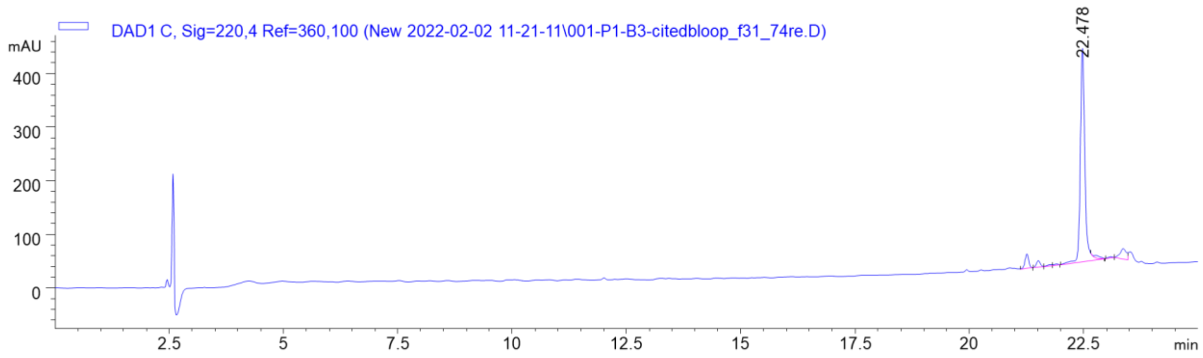

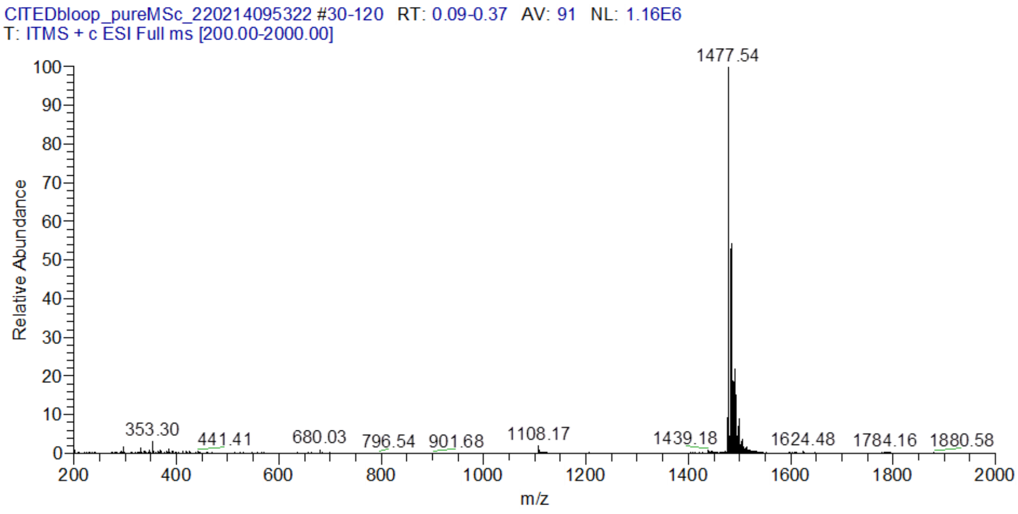

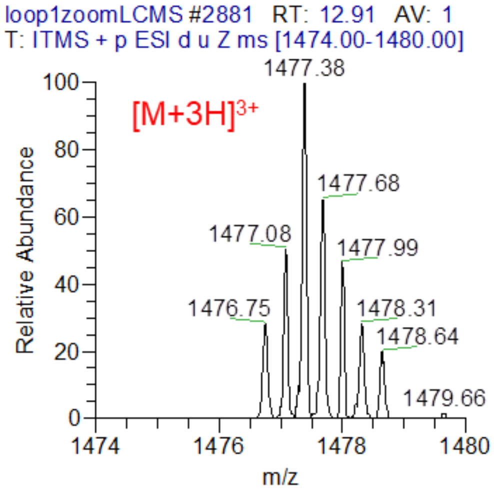

4

Ac-IDTDFIDEEVLMSLVIEMGLDRIKELPELWLG**β^3Q**N**β^3E**F**β^3D**FM-NH_2

EM: 4725.27

MW: 4728.45

$[2M+5H]^{5+}$: 1891.54; $[M+3H]^{3+}$: 1577.39; $[M+4H]^{4+}$: 1183.44

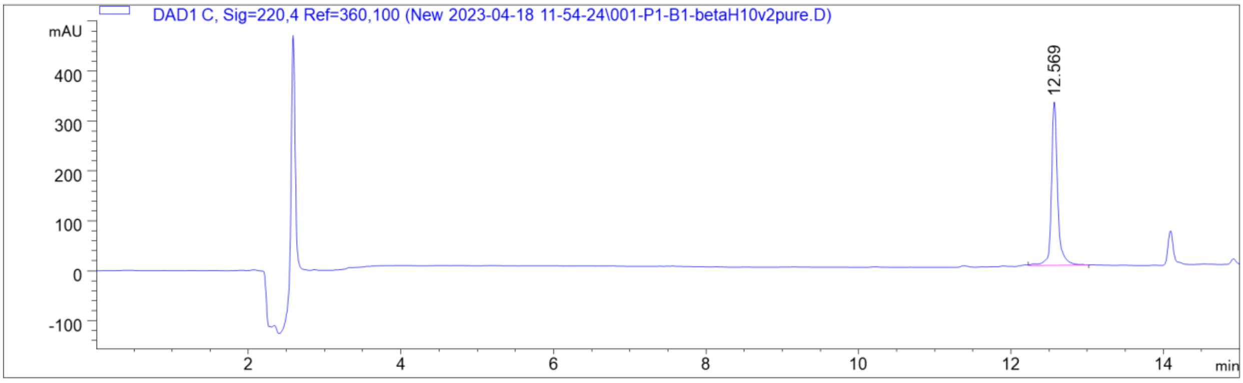

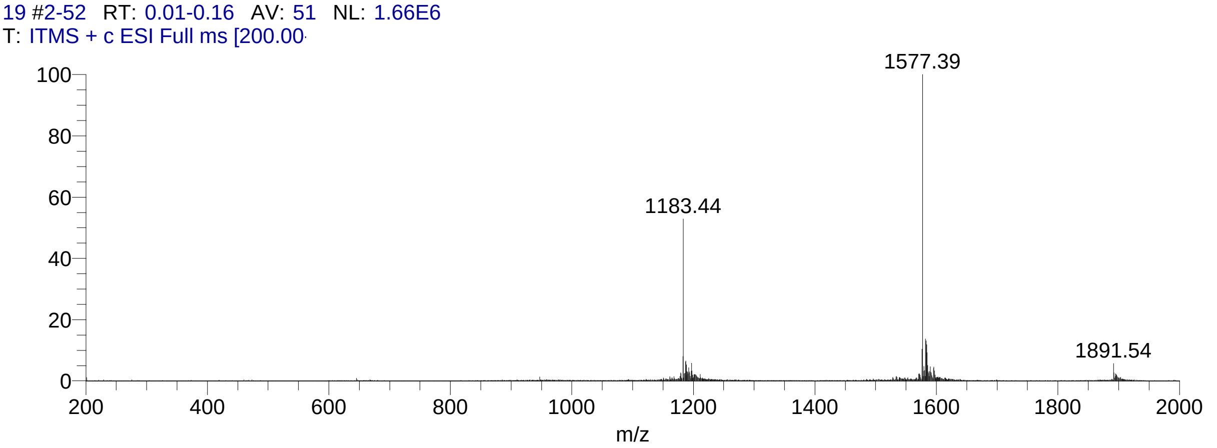

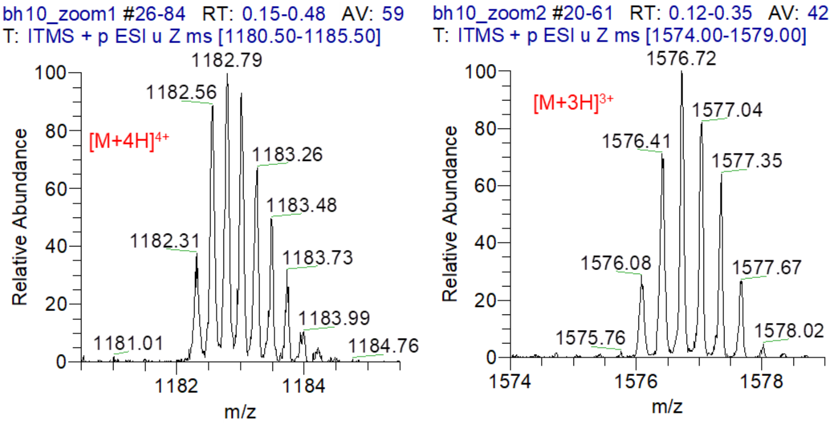

Table S3. Amide proton chemical shifts of p300 in complex with CITED2 variants

Residue		CITED2$_{218-256}$		CITED2ΔN		CITED2$_{224-259}$		1		2		3a		3b		3c		4	
		δ_N (ppm)	δ_H (ppm)	δ_N (ppm)	δ_H (ppm)	δ_N (ppm)	δ_H (ppm)	δ_N (ppm)	δ_H (ppm)	δ_N (ppm)	δ_H (ppm)	δ_N (ppm)	δ_H (ppm)	δ_N (ppm)	δ_H (ppm)	δ_N (ppm)	δ_H (ppm)	δ_N (ppm)	δ_H (ppm)
G		n.a.	n.a.	n.a.	n.a.	n.a.	n.a.	n.a.	n.a.	n.a.	n.a.	n.a.	n.a.	n.a.	n.a.	n.a.	n.a.	n.a.	n.a.
S		n.a.	n.a.	n.a.	n.a.	n.a.	n.a.	n.a.	n.a.	n.a.	n.a.	n.a.	n.a.	n.a.	n.a.	n.a.	n.a.	n.a.	n.a.
A	330	125.61	8.42	125.59	8.41	125.61	8.42	125.58	8.41	125.58	8.41	125.50	8.41	125.48	8.40	125.48	8.41	125.58	8.41
D	331	121.68	8.23	121.70	8.23	121.72	8.22	121.71	8.23	121.69	8.23	121.54	8.25	121.54	8.32	121.54	8.22	121.33	8.22
P	332	n.a.	n.a.	n.a.	n.a.	n.a.	n.a.	n.a.	n.a.	n.a.	n.a.	n.a.	n.a.	n.a.	n.a.	n.a.	n.a.	n.a.	n.a.
E	333	119.21	8.24	119.24	8.22	119.21	8.24	119.21	8.23	119.32	8.24	119.00	8.25	119.44	8.27	119.49	8.29	119.32	8.26
K	334	121.21	7.88	121.21	7.86	120.90	7.86	121.21	7.88	121.15	7.87	120.85	7.85	120.72	7.87	121.24	7.94	120.52	7.86
R	335	117.51	8.02	117.59	8.01	117.49	7.99	117.53	8.02	117.52	8.01	117.56	7.96	117.68	7.98	117.52	8.05	117.65	7.96
K	336	118.46	7.42	118.43	7.40	118.47	7.41	118.41	7.41	118.57	7.42	118.97	7.45	118.58	7.46	118.59	7.46	118.53	7.50
L	337	118.15	7.49	118.17	7.47	118.09	7.51	118.17	7.48	118.11	7.50	118.55	7.45	118.10	7.46	118.35	7.47	117.93	7.50
I	338	120.78	8.14	120.68	8.06	120.68	8.06	120.75	8.14	120.75	8.14	120.74	8.14	120.44	8.00	120.39	8.11	120.39	7.99
Q	339	118.26	8.15	118.21	8.16	118.15	8.12	118.15	8.14	118.30	8.15	117.79	8.16	118.13	8.14	118.37	8.13	118.16	8.09
Q	340	116.71	8.33	116.77	8.31	116.71	8.34	116.71	8.33	116.96	8.39	116.75	8.26	116.68	8.31	116.79	8.31	116.59	8.36
Q	341	118.94	7.73	118.64	7.72	118.61	7.76	118.89	7.72	118.85	7.70	118.48	7.71	118.76	7.69	118.60	7.67	118.90	7.67
L	342	119.33	8.41	119.44	8.46	119.39	8.44	119.27	8.41	119.37	8.43	118.95	8.37	119.21	8.39	119.37	8.39	119.49	8.42
V	343	117.29	8.12	117.50	8.15	117.44	8.14	117.33	8.13	117.10	8.17	117.88	8.12	117.13	8.11	116.69	8.11	117.29	8.11
L	344	118.96	7.88	118.45	7.83	118.79	7.81	118.92	7.84	119.18	7.87	118.95	7.88	118.78	7.88	119.28	7.86	118.92	7.88
L	345	120.90	8.48	120.86	8.51	120.85	8.51	120.86	8.47	120.87	8.46	120.97	8.39	120.93	8.46	121.02	8.44	120.91	8.48
L	346	118.11	8.08	118.11	8.09	118.11	8.09	118.40	8.14	118.40	8.12	118.11	8.09	118.23	8.09	117.91	8.09	118.11	8.09
H	347	116.56	8.65	118.01	8.68	117.88	8.68	116.94	8.63	116.16	8.52	116.57	8.65	116.55	8.64	116.56	8.63	116.53	8.64
A	348	n.a.	n.a.	n.a.	n.a.	n.a.	n.a.	n.a.	n.a.	n.a.	n.a.	n.a.	n.a.	n.a.	n.a.	n.a.	n.a.	n.a.	n.a.
H	349	116.99	8.03	116.27	7.93	116.40	7.96	116.62	8.03	116.67	8.02	117.00	8.04	116.87	8.04	116.74	8.00	116.97	8.02
K	350	118.84	7.36	118.31	7.35	118.39	7.36	118.35	7.33	118.26	7.44	118.83	7.36	119.12	7.42	119.19	7.36	118.87	7.35
C	351	n.a.	n.a.	n.a.	n.a.	n.a.	n.a.	n.a.	n.a.	n.a.	n.a.	n.a.	n.a.	n.a.	n.a.	n.a.	n.a.	n.a.	n.a.
Q	352	118.88	8.23	119.25	8.32	119.21	8.27	119.09	8.27	118.83	8.28	118.40	8.19	118.54	8.21	118.25	8.20	118.85	8.24
R	353	119.15	7.50	118.47	7.46	118.50	7.46	119.16	7.50	119.09	7.45	119.17	7.45	118.89	7.46	118.84	7.42	119.14	7.50
R	354	119.91	7.74	120.32	7.81	120.41	7.84	120.04	7.81	119.74	7.70	12n.a.	7.73	120.04	7.73	12n.a.	7.72	119.90	7.74
E	355	120.12	7.91	120.61	8.01	120.51	8.00	120.37	7.96	120.07	7.89	119.95	7.90	119.92	7.92	119.91	7.91	120.11	7.91
Q	356	118.39	8.01	118.74	8.04	118.84	8.06	118.62	8.05	118.26	8.00	118.32	7.98	118.26	7.97	118.29	7.97	118.41	8.01
A	357	122.38	7.85	122.74	7.93	122.78	7.96	122.50	7.90	122.27	7.83	122.35	7.82	122.34	7.81	122.33	7.82	122.36	7.85
N	358	115.24	8.02	115.45	8.03	115.47	8.05	115.26	8.03	115.14	8.02	115.45	8.02	115.48	8.02	115.45	8.01	115.26	8.02
G	359	108.88	8.14	108.84	8.16	108.83	8.17	108.84	8.14	108.88	8.13	108.93	8.16	108.94	8.17	108.95	8.16	108.88	8.14
E	360	119.85	8.00	119.85	8.00	119.89	8.01	119.81	7.99	119.79	7.99	119.87	8.04	119.87	8.05	119.86	8.05	119.86	8.00
V	361	121.75	8.06	121.47	8.03	121.48	8.04	121.68	8.06	121.71	8.06	121.38	8.01	121.31	7.99	121.30	7.99	121.68	8.06
R	362	125.69	8.44	125.73	8.43	125.73	8.43	125.74	8.44	125.81	8.46	125.50	8.41	125.65	8.41	125.54	8.42	125.68	8.44
Q	363	123.27	8.48	123.06	8.46	123.04	8.46	123.07	8.48	123.41	8.49	123.29	8.47	123.34	8.47	123.36	8.47	123.27	8.48
C	364	126.18	8.49	125.87	8.50	125.83	8.50	126.04	8.49	126.10	8.46	126.10	8.53	126.04	8.52	126.18	8.53	126.18	8.49
N	365	n.a.	n.a.	n.a.	n.a.	n.a.	n.a.	n.a.	n.a.	n.a.	n.a.	n.a.	n.a.	n.a.	n.a.	n.a.	n.a.	n.a.	n.a.
L	366	n.a.	n.a.	n.a.	n.a.	n.a.	n.a.	n.a.	n.a.	n.a.	n.a.	n.a.	n.a.	n.a.	n.a.	n.a.	n.a.	n.a.	n.a.
P	367	n.a.	n.a.	n.a.	n.a.	n.a.	n.a.	n.a.	n.a.	n.a.	n.a.	n.a.	n.a.	n.a.	n.a.	n.a.	n.a.	n.a.	n.a.
H	368	n.a.	n.a.	n.a.	n.a.	n.a.	n.a.	n.a.	n.a.	n.a.	n.a.	n.a.	n.a.	n.a.	n.a.	n.a.	n.a.	n.a.	n.a.

Residue	CITED2$_{218-256}$		CITED2ΔN		CITED2$_{224-259}$		1		2		3a		3b		3c		4	
	δ_N (ppm)	δ_H (ppm)	δ_N (ppm)	δ_H (ppm)	δ_N (ppm)	δ_H (ppm)	δ_N (ppm)	δ_H (ppm)	δ_N (ppm)	δ_H (ppm)	δ_N (ppm)	δ_H (ppm)	δ_N (ppm)	δ_H (ppm)	δ_N (ppm)	δ_H (ppm)	δ_N (ppm)	δ_H (ppm)
C 369	n.a.	n.a.	n.a.	n.a.	n.a.	n.a.	n.a.	n.a.	n.a.	n.a.	n.a.	n.a.	n.a.	n.a.	n.a.	n.a.	n.a.	n.a.
R 370	n.a.	n.a.	n.a.	n.a.	n.a.	n.a.	n.a.	n.a.	n.a.	n.a.	n.a.	n.a.	n.a.	n.a.	n.a.	n.a.	n.a.	n.a.
T 371	114.42	7.40	114.42	7.39	114.42	7.39	114.43	7.36	114.46	7.39	114.42	7.40	114.59	7.58	114.42	7.40	114.44	7.39
M 372	n.a.	n.a.	n.a.	n.a.	n.a.	n.a.	n.a.	n.a.	n.a.	n.a.	n.a.	n.a.	n.a.	n.a.	n.a.	n.a.	n.a.	n.a.
K 373	122.44	9.15	122.20	9.15	122.21	9.16	122.35	9.15	122.35	9.15	122.43	9.16	122.44	9.17	122.45	9.20	122.39	9.14
N 374	117.28	8.00	117.48	7.98	117.49	7.99	117.33	8.01	117.29	8.02	117.11	7.99	117.37	7.91	117.33	7.90	117.29	8.01
V 375	124.14	7.96	124.20	7.95	124.16	7.95	124.18	7.96	124.22	7.96	123.83	7.94	124.11	7.94	124.07	7.95	124.19	7.96
L 376	121.36	8.59	121.36	8.61	121.34	8.62	121.46	8.60	121.47	8.59	121.26	8.54	121.26	8.57	121.27	8.59	121.33	8.59
N 377	117.03	8.09	117.19	8.08	117.19	8.09	116.98	8.09	117.03	8.08	116.97	8.15	117.13	8.13	117.20	8.15	117.12	8.10
H 378	120.54	8.04	120.52	8.04	120.52	8.06	120.52	8.01	120.64	8.04	120.15	7.95	120.46	8.02	120.34	8.00	120.52	8.03
M 379	119.04	9.04	119.07	9.03	119.12	9.04	119.09	9.03	119.04	9.05	118.91	8.97	119.13	9.04	119.08	9.04	119.13	9.05
T 380	111.01	7.43	110.87	7.42	111.04	7.43	110.98	7.43	110.88	7.42	110.94	7.44	110.91	7.49	110.91	7.50	111.05	7.44
H 381	115.70	6.89	115.63	6.90	115.74	6.88	115.62	6.90	115.66	6.91	115.61	6.92	115.62	6.89	115.62	6.90	115.74	6.88
C 382	123.96	7.04	123.96	7.02	123.98	7.04	123.97	7.04	123.94	7.04	123.84	7.01	123.95	7.05	123.96	7.05	124.00	7.05
Q 383	121.83	8.98	121.92	8.94	121.91	8.96	122.19	9.02	121.81	9.06	121.53	8.83	121.46	8.84	121.48	8.91	121.79	9.00
S 384	118.65	8.73	118.63	8.71	118.64	8.73	118.64	8.72	118.64	8.72	118.74	8.73	118.73	8.76	118.70	8.74	118.66	8.74
G 385	110.38	8.21	110.39	8.20	110.40	8.20	110.44	8.22	110.38	8.21	110.28	8.22	110.28	8.20	110.31	8.20	110.34	8.20
K 386	n.a.	n.a.	n.a.	n.a.	n.a.	n.a.	n.a.	n.a.	n.a.	n.a.	n.a.	n.a.	n.a.	n.a.	n.a.	n.a.	n.a.	n.a.
S 387	112.73	7.76	112.71	7.75	112.70	7.76	112.71	7.76	112.75	7.76	112.49	7.74	112.68	7.75	112.69	7.76	112.51	7.74
C 388	124.24	7.42	124.22	7.40	124.24	7.39	124.24	7.41	124.22	7.42	124.26	7.40	124.16	7.40	124.21	7.41	124.20	7.39
Q 389	129.48	9.05	129.53	9.05	129.53	9.05	129.54	9.06	129.56	9.06	128.99	9.00	129.87	9.10	129.55	9.07	129.67	9.07
V 390	127.04	9.16	127.05	9.16	127.01	9.18	127.04	9.17	127.00	9.17	127.60	9.12	126.96	9.17	127.08	9.17	126.99	9.18
A 391	134.21	8.60	134.16	8.59	134.10	8.58	134.18	8.60	134.25	8.61	133.98	8.50	134.06	8.64	133.93	8.59	134.20	8.61
H 392	n.a.	n.a.	n.a.	n.a.	n.a.	n.a.	n.a.	n.a.	n.a.	n.a.	n.a.	n.a.	n.a.	n.a.	n.a.	n.a.	n.a.	n.a.
C 393	126.68	7.90	126.67	7.88	126.66	7.88	126.68	7.90	126.65	7.90	126.85	7.96	126.53	7.86	126.75	7.89	126.68	7.88
A 394	119.16	8.85	119.09	8.82	119.18	8.81	119.15	8.86	119.21	8.87	118.82	8.64	119.26	8.85	119.24	8.84	119.28	8.87
S 395	114.91	9.09	114.83	9.09	114.74	9.15	114.92	9.11	114.90	9.09	114.24	8.92	115.14	9.17	114.86	9.13	115.04	9.18
S 396	120.76	7.76	120.77	7.76	120.75	7.76	120.75	7.76	120.73	7.75	120.14	7.80	120.80	7.72	120.80	7.75	120.67	7.76
R 397	122.63	8.79	122.55	8.74	122.66	8.76	122.62	8.77	122.57	8.78	122.44	8.76	122.55	8.72	122.52	8.69	122.71	8.79
Q 398	118.34	7.84	118.14	7.78	118.08	7.76	118.34	7.84	118.35	7.87	117.13	7.87	118.51	7.81	118.34	7.81	118.21	7.85
I 399	121.97	8.53	121.88	8.56	121.80	8.53	121.90	8.56	121.99	8.54	121.76	8.38	122.06	8.49	122.14	8.54	122.09	8.50
I 400	119.97	8.78	120.04	8.75	120.04	8.76	119.98	8.76	120.01	8.77	120.13	8.75	120.07	8.80	120.03	8.75	120.13	8.77
S 401	113.94	7.87	114.07	7.76	114.21	7.79	113.87	7.84	113.85	7.86	114.14	7.94	114.15	7.87	114.00	7.83	114.04	7.89
H 402	118.35	7.82	118.46	7.79	118.42	7.85	118.36	7.83	118.42	7.84	118.40	7.76	118.48	7.91	118.53	7.85	118.42	7.91
W 403	119.36	8.35	119.23	8.22	119.22	8.29	119.30	8.29	119.29	8.35	119.24	8.33	119.48	8.38	119.37	8.34	119.34	8.38
K 404	115.71	8.36	115.18	8.28	115.20	8.32	115.39	8.30	115.58	8.32	115.70	8.38	115.75	8.38	115.71	8.37	115.76	8.37
N 405	113.52	6.97	113.79	7.05	113.80	7.07	113.64	7.03	113.51	7.02	113.53	7.00	113.51	7.02	113.57	7.01	113.44	7.00
C 406	124.46	7.22	124.52	7.29	124.39	7.32	124.46	7.28	124.47	7.27	124.44	7.24	124.47	7.27	124.52	7.25	124.40	7.27
T 407	n.a.	n.a.	n.a.	n.a.	n.a.	n.a.	n.a.	n.a.	n.a.	n.a.	n.a.	n.a.	n.a.	n.a.	n.a.	n.a.	n.a.	n.a.
R 408	n.a.	n.a.	n.a.	n.a.	n.a.	n.a.	n.a.	n.a.	n.a.	n.a.	n.a.	n.a.	n.a.	n.a.	n.a.	n.a.	n.a.	n.a.
H 409	n.a.	n.a.	n.a.	n.a.	n.a.	n.a.	n.a.	n.a.	n.a.	n.a.	n.a.	n.a.	n.a.	n.a.	n.a.	n.a.	n.a.	n.a.
D 410	n.a.	n.a.	n.a.	n.a.	n.a.	n.a.	n.a.	n.a.	n.a.	n.a.	n.a.	n.a.	n.a.	n.a.	n.a.	n.a.	n.a.	n.a.
C 411	122.57	6.94	122.70	6.95	122.55	6.93	122.64	6.94	122.58	6.94	122.51	6.93	122.49	6.93	122.56	6.93	122.52	6.94

Residue		CITED2$_{218-256}$		CITED2ΔN		CITED2$_{224-259}$		1		2		3a		3b		3c		4	
		δ_N (ppm)	δ_H (ppm)	δ_N (ppm)	δ_H (ppm)	δ_N (ppm)	δ_H (ppm)	δ_N (ppm)	δ_H (ppm)	δ_N (ppm)	δ_H (ppm)	δ_N (ppm)	δ_H (ppm)	δ_N (ppm)	δ_H (ppm)	δ_N (ppm)	δ_H (ppm)	δ_N (ppm)	δ_H (ppm)
P	412	n.a.	n.a.	n.a.	n.a.	n.a.	n.a.	n.a.	n.a.	n.a.	n.a.	n.a.	n.a.	n.a.	n.a.	n.a.	n.a.	n.a.	n.a.
V	413	119.95	8.29	120.03	8.25	120.01	8.27	119.95	8.28	119.88	8.30	119.88	8.28	120.09	8.31	120.12	8.30	119.94	8.29
C	414	118.95	8.38	118.75	8.36	118.66	8.37	118.92	8.40	118.93	8.40	118.95	8.37	118.91	8.38	118.96	8.36	118.78	8.32
L	415	n.a.	n.a.	n.a.	n.a.	n.a.	n.a.	n.a.	n.a.	n.a.	n.a.	n.a.	n.a.	n.a.	n.a.	n.a.	n.a.	n.a.	n.a.
P	416	n.a.	n.a.	n.a.	n.a.	n.a.	n.a.	n.a.	n.a.	n.a.	n.a.	n.a.	n.a.	n.a.	n.a.	n.a.	n.a.	n.a.	n.a.
L	417	n.a.	n.a.	n.a.	n.a.	n.a.	n.a.	n.a.	n.a.	n.a.	n.a.	n.a.	n.a.	n.a.	n.a.	n.a.	n.a.	n.a.	n.a.
K	418	119.34	7.82	119.18	7.70	119.16	7.72	118.68	7.80	119.18	7.87	119.20	7.84	119.34	7.85	118.65	7.82	119.36	7.83
N	419	117.44	8.29	117.88	8.32	117.86	8.31	117.80	8.26	117.34	8.27	117.56	8.28	117.48	8.29	117.48	8.28	117.42	8.29
A	420	123.06	7.83	123.44	8.05	123.49	8.04	123.48	7.88	123.10	7.80	123.15	7.84	123.08	7.83	123.12	7.83	123.05	7.83
G	421	106.29	8.17	106.57	8.19	106.60	8.20	106.51	8.23	106.24	8.15	106.41	8.17	106.32	8.17	106.36	8.17	106.28	8.17
D	422	120.24	8.00	120.13	8.08	120.15	8.08	120.20	8.00	120.23	8.00	120.21	8.01	120.23	8.00	120.23	8.00	120.25	8.00
K	423	121.83	8.07	121.72	8.10	121.73	8.11	121.75	8.06	121.92	8.08	121.82	8.07	121.85	8.07	121.85	8.08	121.84	8.07
R	424	127.73	7.84	127.48	7.85	127.50	7.86	127.75	7.87	127.86	7.86	127.74	7.86	127.72	7.85	127.75	7.85	127.73	7.84

Table S4. Assigned CH_3 chemical shifts of p300 in complex with the different CITED2 variants

	$CITED2_{218-256}$		CITED2ΔN		$CITED2_{224-259}$		1		2		3a		3b		3c		4	
	δ_C	δ_H	δ_C	δ_H	δ_C	δ_H	δ_C	δ_H	δ_C	δ_H	δ_C	δ_H	δ_C	δ_H	δ_C	δ_H	δ_C	δ_H
L337 CD2-HD21	22.29	0.90	22.43	0.87	22.29	0.90	22.39	0.90	22.38	0.90	22.29	0.90	22.29	0.90	22.29	0.90	22.61	0.89
I338 CD1-HD11	13.87	0.52	13.99	0.51	13.79	0.55	14.00	0.53	13.99	0.53	13.91	0.50	13.71	0.39	13.90	0.51	14.02	0.55
I338 CG2-HG21	17.66	0.87	17.61	0.83	17.52	0.81	17.67	0.87	17.69	0.87	18.03	0.84	17.83	0.75	17.85	0.85	17.76	0.75
L342 CD1-HD11	20.70	0.49	21.02	0.51	20.89	0.54	20.80	0.48	20.78	0.48	21.04	0.51	20.95	0.51	20.99	0.51	20.86	0.49
L342 CD2-HD21	26.61	0.85	26.77	0.80	26.61	0.83	26.57	0.79	26.60	0.81	26.75	0.85	26.79	0.87	26.96	0.88	26.78	0.84
V343 CG2-HG21	24.29	1.03	24.53	1.01	24.35	1.03	24.53	1.02	24.54	1.04	24.16	1.04	24.12	1.03	23.79	1.04	24.41	1.02
L345 CD2-HD21	23.55	1.00	23.48	0.98	23.34	1.01	23.57	1.00	23.72	0.99	23.53	0.97	23.30	0.98	23.54	0.99	23.71	0.99
L346 CD1-HD11	25.76	0.06	25.36	-0.16	25.19	-0.11	25.55	-0.03	25.82	0.11	25.79	0.02	25.89	0.02	25.79	-0.01	25.81	0.05
L346 CD2-HD21	21.29	0.10	21.07	0.11	21.33	0.11	21.18	0.19	21.82	0.20	21.49	0.09	21.44	0.11	21.29	0.10	21.49	0.11
A348 CB-HB1	18.98	1.51	19.01	1.49	18.84	1.53	19.12	1.52	19.09	1.52	19.10	1.51	19.11	1.51	19.06	1.51	19.08	1.50
A357 CB-HB1	18.99	1.47	19.23	1.44	19.10	1.47	19.10	1.48	19.05	1.48	19.13	1.47	19.12	1.49	19.10	1.48	19.12	1.46
V361 CG1-HG11	20.64	0.96	20.76	0.92	20.65	0.95	20.80	0.95	20.78	0.95	20.67	0.95	20.69	0.96	20.69	0.95	20.79	0.94
L366 CD1-HD11	24.29	0.37	24.48	0.39	24.32	0.42	24.22	0.35	24.32	0.37	24.67	0.51	24.71	0.50	24.69	0.50	24.37	0.37
L366 CD2-HD21	23.60	0.41	23.82	0.42	23.59	0.46	23.60	0.41	23.62	0.41	23.64	0.53	23.46	0.60	23.25	0.61	23.74	0.40
T371 CG2-HG21	21.93	1.26	22.08	1.23	21.90	1.26	21.99	1.26	22.04	1.26	22.03	1.28	21.84	1.23	21.89	1.24	22.02	1.25
M372 CE-HE1	18.12	1.65	18.36	1.62	18.20	1.65	18.30	1.65	18.21	1.65	18.01	1.67	17.90	1.60	17.99	1.64	18.18	1.65
V375 CG1-HG11	21.05	1.01	21.15	0.99	20.99	1.02	21.14	1.02	21.10	1.01	21.16	1.03	21.10	1.00	21.12	1.01	21.12	1.00
V375 CG2-HG21	23.58	1.24	23.67	1.21	23.46	1.24	23.67	1.24	23.73	1.24	23.61	1.26	23.59	1.24	23.51	1.24	23.70	1.24
M379 CE-HE1	16.46	2.02	16.52	1.99	16.37	2.01	16.60	2.02	16.46	2.02	16.63	2.03	16.46	2.02	16.46	2.02	16.46	2.02
V380 CG1-HG11	20.02	0.47	20.12	0.43	19.94	0.46	20.11	0.47	20.10	0.47	20.15	0.43	20.09	0.46	20.10	0.45	20.08	0.46
V380 CG2-HG21	22.58	0.67	22.74	0.65	22.56	0.67	22.71	0.68	22.68	0.67	22.67	0.63	22.73	0.70	22.72	0.69	22.69	0.67
A381 CB-HB1	17.52	0.91	17.61	0.88	17.53	0.91	17.62	0.91	17.61	0.91	17.72	0.95	17.50	0.89	17.65	0.90	17.60	0.90
A394 CB-HB1	17.48	1.48	17.64	1.45	17.39	1.40	17.58	1.48	17.60	1.49	17.93	1.52	17.71	1.48	17.61	1.47	17.48	1.48
I399 CD1-HD11	14.00	0.83	14.16	0.80	13.99	0.82	14.18	0.83	14.19	0.83	14.09	0.84	14.08	0.80	14.11	0.84	14.15	0.80
I399 CG2-HG21	18.92	0.91	19.27	0.89	19.02	0.91	19.05	0.91	19.00	0.91	18.88	0.92	18.93	0.91	19.01	0.92	19.00	0.90
I400 CD1-HD11	12.59	0.76	12.34	0.76	12.28	0.76	12.77	0.76	12.87	0.77	12.54	0.74	12.70	0.76	12.61	0.76	12.79	0.76
I400 CG2-HG21	17.74	0.97	17.77	0.97	17.65	0.96	17.89	0.96	17.76	0.97	17.72	0.95	17.79	0.97	17.74	0.96	17.80	0.97
V413 CG1-HG11	21.64	0.99	21.71	0.96	21.45	0.94	21.64	0.99	21.68	1.00	21.85	0.98	21.79	0.96	21.89	0.99	21.53	0.91
L417 CD2-HD21	24.70	0.70	25.20	0.62	25.00	0.64	24.99	0.64	24.97	0.68	24.70	0.70	24.60	0.70	24.47	0.71	24.78	0.69

References

(1) Berlow, R. B.; Dyson, H. J.; Wright, P. E. Hypersensitive Termination of the Hypoxic Response by a Disordered Protein Switch. *Nature* **2017**, *543* (7645), 447–451. https://doi.org/10.1038/nature21705.

(2) Berlow, R. B.; Dyson, H. J.; Wright, P. E. Multivalency Enables Unidirectional Switch-like Competition between Intrinsically Disordered Proteins. *Proc. Natl. Acad. Sci.* **2022**, *119* (3), e2117338119. https://doi.org/10.1073/pnas.2117338119.

(3) Hó bor, F.; Zsó fia Hegedü, ab; Avila Ibarra, A.; Vencel Petrovicz, de L.; Bartlett, G. J.; Richard Sessions, def B.; Andrew Wilson, de J.; Edwards, T. A. Understanding P300-Transcription Factor Interactions Using Sequence Variation and Hybridization †. **2022**, *3*, 546–550. https://doi.org/10.1039/d2cb00026a.

(4) Usui-Ouchi, A.; Aguilar, E.; Murinello, S.; Prins, M.; Gantner, M. L.; Wright, P. E.; Berlow, R. B.; Friedlander, M. An Allosteric Peptide Inhibitor of HIF-1α Regulates Hypoxia-Induced Retinal Neovascularization. *Proc. Natl. Acad. Sci.* **2020**, *117* (45), 28297–28306. https://doi.org/10.1073/pnas.2017234117.

(5) Wood, C. W.; Ibarra, A. A.; Bartlett, G. J.; Wilson, A. J.; Woolfson, D. N.; Sessions, R. B. BAlaS: Fast, Interactive and Accessible Computational Alanine-Scanning Using BudeAlaScan. *Bioinformatics* **2020**, *36* (9), 2917–2919. https://doi.org/10.1093/BIOINFORMATICS/BTAA026.

(6) *Role of Backbone Dynamics in Modulating the Interactions of Disordered Ligands with the TAZ1 Domain of the CREB-Binding Protein | Biochemistry*. https://pubs.acs.org/doi/10.1021/acs.biochem.8b01290 (accessed 2023-03-28).

(7) Kyle, H. F.; Wickson, K. F.; Stott, J.; Burslem, G. M.; Breeze, A. L.; Tiede, C.; Tomlinson, D. C.; Warriner, S. L.; Nelson, A.; Wilson, A. J.; Edwards, T. A. Exploration of the HIF-1α/P300 Interface Using Peptide and Adhiron Phage Display Technologies. *Mol. Biosyst.* **2015**, *11* (10), 2738–2749. https://doi.org/10.1039/c5mb00284b.

(8) Gasteiger, E.; Hoogland, C.; Gattiker, A.; Duvaud, S.; Wilkins, M. R.; Appel, R. D.; Bairoch, A. Protein Identification and Analysis Tools on the ExPASy Server. In *The Proteomics Protocols Handbook*; Walker, J. M., Ed.; Humana Press: Totowa, NJ, 2005; pp 571–607. https://doi.org/10.1385/1-59259-890-0:571.

(9) Hobor, F.; Hegedüs, Z.; Ibarra, A. A.; Petrovicz, V.; Bartlett, G.; Sessions, R. B.; Wilson, A.; Edwards, T. A. Understanding P300-Transcription Factor Interactions Using Sequence Variation and Hybridization. *RSC Chem. Biol.* **2022**, *3*, 546–550. https://doi.org/10.1039/d2cb00026a.

(10) Keller, S.; Vargas, C.; Zhao, H.; Piszczek, G.; Brautigam, C. A.; Schuck, P. High-Precision Isothermal Titration Calorimetry with Automated Peak-Shape Analysis. *Anal. Chem.* **2012**, *84* (11), 5066–5073. https://doi.org/10.1021/AC3007522.

(11) Zhao, H.; Piszczek, G.; Schuck, P. SEDPHAT – A Platform for Global ITC Analysis and Global Multi-Method Analysis of Molecular Interactions. *Methods* **2015**, *76*, 137–148. https://doi.org/10.1016/J.YMETH.2014.11.012.

(12) Houtman, J. C. D.; Brown, P. H.; Bowden, B.; Yamaguchi, H.; Appella, E.; Samelson, L. E.; Schuck, P. Studying Multisite Binary and Ternary Protein Interactions by Global Analysis of Isothermal Titration Calorimetry Data in SEDPHAT: Application to Adaptor Protein Complexes in Cell Signaling. *Protein Sci. Publ. Protein Soc.* **2007**, *16* (1), 30. https://doi.org/10.1110/PS.062558507.

(13) Krainer, G.; Broecker, J.; Vargas, C.; Keller, S. Quantifying High-Affinity Binding of Hydrophobic Ligands by Isothermal Titration Calorimetry. *Anal. Chem.* **2012**, *84*, 10715–10722.

(14) Sigurskjold, B. W. Exact Analysis of Competition Ligand Binding by Displacement Isothermal Titration Calorimetry. *Anal. Biochem.* **2000**, *277*, 260–266. https://doi.org/10.1006/abio.1999.4402.

(15) Brautigam, C. A. Calculations and Publication-Quality Illustrations for Analytical Ultracentrifugation Data. *Methods Enzymol.* **2015**, *562*, 109–133. https://doi.org/10.1016/BS.MIE.2015.05.001.

(16) Lee, C. W.; Ferreon, J. C.; Ferreon, A. C. M.; Arai, M.; Wright, P. E. Graded Enhancement of P53 Binding to CREB-Binding Protein (CBP) by Multisite Phosphorylation. *Proc. Natl. Acad. Sci. U. S. A.* **2010**, *107* (45), 19290–19295. https://doi.org/10.1073/pnas.1013078107.

(17) Roehrl, M. H. A.; Wang, J. Y.; Wagner, G. A General Framework for Development and Data Analysis of Competitive High-Throughput Screens for Small-Molecule Inhibitors of Protein-Protein Interactions by Fluorescence Polarization. *Biochemistry* **2004**, *43* (51), 16056–16066. https://doi.org/10.1021/bi048233g.

(18) Lee, W.; Tonelli, M.; Markley, J. L. NMRFAM-SPARKY: Enhanced Software for Biomolecular NMR Spectroscopy. *Bioinformatics* **2015**, *31* (8), 1325–1327. https://doi.org/10.1093/bioinformatics/btu830.
